# Transcriptomic and Proteomic Analysis of *Oenococcus oeni* Adaptation to Wine Stress Conditions

**DOI:** 10.3389/fmicb.2016.01554

**Published:** 2016-09-30

**Authors:** Mar Margalef-Català, Isabel Araque, Albert Bordons, Cristina Reguant, Joaquín Bautista-Gallego

**Affiliations:** Departament de Bioquímica i Biotecnologia, Facultat d'Enologia, Universitat Rovira i VirgiliTarragona, Spain

**Keywords:** *Oenococcus oeni*, malolactic fermentation, transcriptomic, proteomic, stress, wine

## Abstract

*Oenococcus oeni*, the main lactic acid bacteria responsible for malolactic fermentation in wine, has to adapt to stressful conditions, such as low pH and high ethanol content. In this study, the changes in the transcriptome and the proteome of *O. oeni* PSU-1 during the adaptation period before MLF start have been studied. DNA microarrays were used for the transcriptomic analysis and two complementary proteomic techniques, 2-D DIGE and iTRAQ labeling were used to analyze the proteomic response. One of the most influenced functions in PSU-1 due to inoculation into wine-like medium (WLM) was translation, showing the over-expression of certain ribosomal genes and the corresponding proteins. Amino acid metabolism and transport was also altered and several peptidases were up regulated both at gene and protein level. Certain proteins involved in glutamine and glutamate metabolism showed an increased abundance revealing the key role of nitrogen uptake under stressful conditions. A strong transcriptional inhibition of carbohydrate metabolism related genes was observed. On the other hand, the transcriptional up-regulation of malate transport and citrate consumption was indicative of the use of L-malate and citrate associated to stress response and as an alternative energy source to sugar metabolism. Regarding the stress mechanisms, our results support the relevance of the thioredoxin and glutathione systems in the adaptation of *O. oeni* to wine related stress. Genes and proteins related to cell wall showed also significant changes indicating the relevance of the cell envelop as protective barrier to environmental stress. The differences found between transcriptomic and proteomic data suggested the relevance of post-transcriptional mechanisms and the complexity of the stress response in *O. oeni* adaptation. Further research should deepen into the metabolisms mostly altered due to wine conditions to elucidate the role of each mechanism in the *O. oeni* ability to develop MLF.

## Introduction

Malolactic fermentation (MLF) occurs in wine spontaneously and, alternatively, can be induced inoculating selected strains of lactic acid bacterium (LAB), mainly *Oenococcus oeni*, usually after alcoholic fermentation (Betteridge et al., 2015). The wide range of physiological characteristics and the ability to cope with several environmental stresses make *O. oeni* the main responsible for MLF. This process consists in the conversion of L-malate to L-(+)-lactate and CO_2_ and is required in wine, mainly from red grape varieties, because it confers positive sensory traits and improves wine's microbiological stability (Lonvaud-Funel, 1999; Mills et al., 2005).

Wine is a harsh environment for *O. oeni* due to its physicochemical characteristics, such as low pH, ethanol and SO_2_ content, which can negatively affect bacterial survival and consequently MLF development. In contrast to the diversity of stress response mechanisms described in *Bacillus subtilis* (Hecker et al., 1996; Hecker and Völker, 1998), the model organism for Gram-positive bacteria, no gene encoding an alternative sigma factor or any other known regulator of stress response, such as HrcA, could be identified in *O. oeni*. Grandvalet et al. (2005) described in *O. oeni* the CtsR as the regulator for most of molecular chaperone genes. Different studies have characterized some of the stress response genes in *O. oeni*, such as *clp, grpE, groES, hsp18, hdc, ftsH, omrA, cfa, atpB*, and *trxA*, among others (Jobin et al., 1997; Guzzo et al., 2000; Bourdineaud et al., 2003, 2004; Beltramo et al., 2004, 2006; Bourdineaud, 2006; Spano and Massa, 2006; Olguín et al., 2009, 2010). These studies revealed that *O. oeni* has developed cellular mechanisms that make it more resistant to adverse conditions than other LAB species (Beltramo et al., 2006). The knowledge of the stress response machinery of this bacterium is key to understand the ability of adaptation to wine environment of each strain and select the best starter culture.

Thanks to the publication over the recent years of the genomes of different *O. oeni* strains in the National Center for Biotechnology Information (NCBI), nowadays it is possible the global study of stress response by “omics” technologies. There are two published studies of *O. oeni* combining transcriptomic and proteomic analysis, applying DNA microarrays and 2DE or 2D-DIGE: Olguín et al. (2015) studied the effect of ethanol addition during growth (after 1 h), and Costantini et al. (2015) studied wine like media adaptation during 24 h. The first proteomic study was from Silveira et al. (2004) and showed that both ethanol stress and adaptation significantly changed the protein profiles of *O. oeni* cells. Later, Cecconi et al. (2009) performed a proteomic study using 2DE examining *O. oeni* adaptation to wine conditions. Other authors have studied oenological starters to determine their proteomic profile (Cafaro et al., 2014; Napoli et al., 2014).

In this work, we combined a transcriptomic and proteomic approach to elucidate the changes involved in the adaptation of *O. oeni* PSU-1 to wine-like conditions, evaluating the period between the inoculation and the beginning of MLF. The transcriptomic analysis was developed using DNA microarrays designed for PSU-1 strain and the results obtained were validated by real-time qPCR. For the proteomic study two complementary techniques were employed: 2D-DIGE and iTRAQ labeling. 2D-DIGE technique (Unlü et al., 1997) relies on a pre-electrophoretic labeling, allowing sample multiplexing in the same gel. This gel-dependent technique has been used in several studies with other LAB species (Mehmeti et al., 2011; Koponen et al., 2012; Genovese et al., 2013). However, the variable reproducibility of this technique along with the difficult automation and detection of low abundance and membrane hydrophobic proteins have led to a wide variety of off-gel methodologies for protein quantification. In order to complement DIGE analysis, in this work it was used a tandem mass spectrometry (MS/MS) coupled with isobaric tags for relative and absolute quantification (iTRAQ) (Ross et al., 2004) labeling to enable the identification and quantification of differentially expressed proteins in specific times. The combination of liquid chromatography (LC) and electrospray ionization MS/MS analysis is an emerging powerful methodology enabling quantification and comparison of protein levels directly from samples with greater efficiency and accuracy. This is the first proteomic analysis using this gel-free technique with *O. oeni*.

## Materials and methods

### Growth conditions and MLF monitoring

The strain used in this study was *O. oeni* PSU-1, the only in its species with the genome fully annotated (Mills et al., 2005). Stock cultures (kept frozen at −80°C) were grown in MRS broth medium (De Man et al., 1960) supplemented with 4 g/L L-malic acid and 5 g/L fructose at pH 5.0 at 28°C in a 10% CO_2_ atmosphere. Cells were collected at the end of the exponential phase (OD_600nm_ = 1.4 − 1.6) and inoculated into the medium. Then, cells were harvested at the end of the exponential phase and inoculated (2% v/v) into 5 L screw-cap bottles of wine-like medium (WLM). WLM was prepared following (Bordas et al., 2015) containing 12% ethanol (v/v) at pH 3.4. The bottles were incubated at 20°C. The assays were run in triplicate. Measurements of L-malic acid consumption were performed with the multianalyser Miura One (I.S.E. S.r.l, Guidonia, Italy) and the enzymatic kit ready to use (TDI SL, Barcelona, Spain) in order to determine the beginning and evolution of MLF.

### RNA and protein extraction

After the inoculation into WLM, samples at different times (0, 0.5, 1, 2, 4, 6, and 8 h) were taken for RNA and protein extraction. For RNA extraction, 20 mL were collected from WLM, or 0.3 mL from MRS culture used for inoculation (0 h). Samples were centrifuged at 10,000 × *g* for 5 min at 4°C, supernatant was removed and pellet was washed with 10 mM Tris-HCl prepared with diethylpyrocarbonate-treated water (DEPC), and then frozen in liquid nitrogen and kept at −80°C until RNA extraction. High Pure RNA Isolation Kit (Roche, Mannheim, Germany) was used for RNA extraction following the instructions of the manufacturer with some modifications, such as lysis with lysozyme dissolved in 10 mM Tris-HCl buffer DEPC, at 50 mg/mL during 30 min at 37°C. RNA was treated with Turbo DNA-free (Life Technologies, USA). Total acid nucleic concentrations were calculated using a Nanodrop 1000 spectrophotometer (Thermo Fisher Scientific, Bremen, Germany).

For protein extract preparation, 800 mL of WLM, or 35 mL from MRS culture used for inoculation (0 h), were centrifuged at 5000 × *g* rpm for 15 min. Supernatant was removed and pellet was washed twice with 10 mM Tris-HCl buffer at pH 8, frozen in liquid nitrogen and kept at −80°C until protein extraction. Cell pellets were then resuspended to a final OD_600_ = 30 in a solution of 0.1 M Tris-HCl at pH 7.5, mixed with protease inhibitor cocktail from Roche. Cells were disrupted using One-shot disruptor (Constant Systems Ltd.) at 5°C, applying twice a 2.7 kbar pressure. Protein suspension was centrifuged at 4500 × *g* for 15 min at 4°C to remove cell debris and the supernatant was frozen in liquid nitrogen until protein analysis.

### Transcriptomic analysis

#### DNA microarray description, labeling, and hybridization

Microarrays (090324_*Oenococcus oeni* expression 4-plex array), based on PSU-1 genome, were developed by Roche NimbleGen (Madison, WI, USA) and samples were analyzed at the Functional Genomics Core of the Institute for Research in Biomedicine (IRB, Barcelona, Spain) as described by Olguín et al. (2015). The results were submitted to GEO (Gene Expression Omnibus Database, NCBI) under accession number GSE85137.

#### Microarray results validation by real-time qPCR

Nucleotide sequences of *O. oeni* strain PSU-1 (NC_008528) were obtained from the NCBI. Several genes were selected for real-time qPCR validation of the microarray data. The primers used for these analyses are shown in Table 1. Some genes were selected due to their involvement in stress response according to previous studies (Jobin et al., 1999b; Beltramo et al., 2006; Olguín et al., 2009; Bordas et al., 2013; Margalef-Català et al., 2017) and others were randomly selected with the sole objective of validating the methodology. Reverse transcription, real-time qPCR and primer design were performed according to Olguín et al. (2009). The Primer Express® Software was used to select primer sequences and analyze secondary structures and dimer formation. The absence of chromosomal DNA contamination was confirmed by qPCR. For the normalization of qPCR data (Vandesompele et al., 2002; Sumby et al., 2012; Cafaro et al., 2014) four genes (*ldhD, dpoIII, gyrA*, and *gyrB*) were evaluated as internal controls, using the primers described in Table 1. Of these, *ldhD* and *gyrA* genes showed the lowest variation under the experimental conditions used (data not shown) and were chosen as internal controls. The amplification efficiency was calculated as in Olguín et al. (2015).

**Table 1:** **Gene descriptions and the corresponding primer sequences used for validation of microarray results by real-time qPCR**.

**Gene symbol and old tag (OEOE_)**	**Sequence (5′–3′)**	**Amplicon length (bp)**	**References**	**Microarray^a^**	**qPCR^b^**
**1 h**					
RS04745/0988 diacylglycerol kinase	Fw-TTGGGTCGGCATTTACTTTC Rv-CCAACCGTAACCCATAACCA	57	This work	−1.05	0.71
RS06455/1342 PTS sugar transporter subunit IIA	Fw-TGGTCGGAAATCAAGAAAGC Rv-TCGGAAACTCCGTAATCGAC	104	This work	−1.61	−0.40
RS02005/0417 citrate lyase	Fw-GCACGTGAACTGCTGAAAAA Rv-TGAGTGTTCCGATTCCACAA	94	This work	1	3.28
RS02030/0422 Citrate lyase (*citE*)	Fw-CCGCACGATGATGTTTGTTCC Rv-GCTCAAAGAAACGGCATCTTCC	108	Olguín et al., 2009	1.49	3.69
RS01385/0289 heat-shock protein Hsp20 (*hsp18)*	Fw-CGGTATCAGGAGTTTTGAGTTC Rv-CGTAGTAACTGCGGGAGTAATTC	102	Beltramo et al., 2006	−0.42	1.13
RS02715/0570 ATP dependent Clp protease proteolytic subunit (*clpp*)	Fw-CGGTACCAAAGGCAAGCGTTTTAT Rv-CTCTTCCGAGTCTTCAAAAGTTGAT	131	Beltramo et al., 2006	0.41	0.15
RS05660/1176 cyclopropane-fatty-acyl-phospholipid synthase (*cfa*)	Fw-TGGTATTACATTGAGCGAGGAG Rv-CGTCTTTGAGATCACGATAATCC	113	Beltramo et al., 2006	−1.11	−0.17
**2 h**					
RS01480/0310 phosphoglycerate mutase	Fw-CCGAAACCGCACAAAAGTAT Rv-CTTCGTGACCCAAAAGTGGT	87	This work	1.22	3.66
RS04220/0881 Acyl carrier protein phosphodiesterase	Fw-GATCTCCCGAAGGATCAACA Rv-AAAATTCATCCAGCCATTCG	61	This work	1.51	3.90
RS06410/1332 2-dehydro-3-deoxyphosphooctonate aldolase	Fw-CCAAAATCGACCCAATTACG Rv-TCCCTCATCTCGATCAGACC	106	This work	1.05	4.03
**4 h**					
RS04710/0981 peptidase	Fw-GAATTGGCTCCCGACACTAA Rv-TGACGATCCTTTGGAGCAAT	71	This work	1.15	3.72
**8 h**					
RS00900/0189 disulfide bond formation protein	Fw-GCTGTTGGTGTTTCGGTTTT Rv-GCTCCAGGCAAAGTTTGAAG	83	This work	1.00	3.60
RS01290/0269 phosphonate ABC transporter ATP-binding protein	Fw-TTTTCAGGATCCGAAGATGG Rv-GCAACAAATTTTCGGCAACT	59	This work	2.65	4.38
RS02980/0624 cobalt ABC transporter	Fw-ACTTTGGCTCCTCTGGTTGA Rv-CAGCATTCATCGGTTTGCTA	100	This work	1.24	2.77
RS04600/0959 Xaa-Pro aminopeptidase	Fw-GTGGAAGTGGTGAAGGGATG Rv-GGTCGACTCCATTTGGAAGA	108	This work	1.93	3.82
RS05245/1092 oligoendopeptidase F	Fw-CGGCAAATACTGGCAAAGAT Rv-TGGACCCCATATGGAAATGT	55	This work	1.90	3.82
RS06245/1296 branched-chain amino acid aminotransferase	Fw-TTTCCCAGAAGACCGTTTTG Rv-AAGTTGCACCGGAACCATAC	89	This work	3.02	5.71
RS07835/1625 Thiol-disulfide isomerase *trxA2*	Fw-TGGCAGTCTTTGAAACCTGA Rv-CCAAGGGTCGCAATTTAATG	105	Margalef-Català et al., 2017	−1.07	0.44
RS08215/1702 Thioredoxin *trxA3*	Fw-GCCACTTGGTGTACCCCTTGT Rv-TCCATTTGCCGTTTCCTGGTTT	120	Margalef-Català et al., 2017	−0.81	0.92
RS02695/0566 Thioredoxin reductase *trxB*	Fw-ATGCCAGCTCAACTCGTTTT Rv-GTCGCTCCGCTAGCAACTAT	139	Margalef-Català et al., 2017	1.23	3.45
RS00770/0163 Ferredoxin-NADP reductase *fdr*	Fw-AGCGAAGTTGCCGATAAAGA Rv-TATCACGCCGATGAATCAAA	115	Margalef-Català et al., 2017	1.35	3.54
RS05740/1191 glutathione reductase *(gshR*)	Fw-GGCATTATCACCGAGCTGTT Rv-TCCCGAAGAAGCAAAGAAGA	106	Bordas et al., 2013	−0.98	−0.76
**qPCR CONTROL GENES**					
RS01985/0413 D-lactate dehydrogenase (*ldhD*)	Fw-GCCGCAGTAAAGAACTTGATG Rv-TGCCGACAACACCAACTGTTT	102	Desroche et al., 2005		
RS04805/1000 DNA polymerase III subunit alpha *(dpoIII*)	Fw-AATTCGCACGGATTGTTTTC Rv-GCGAACCAGCATAGGTCAAT	103	Stefanelli, 2014		
RS04780/0995 DNA primase (*dna G*)	Fw-TGTGGACGGAGTGGCAATGT Rv-CAGTATTTTCTGTATATTTACTATCG	127	Desroche et al., 2005 Margalef-Català et al., 2017		
RS00030/0006 DNA gyrase subunitA (*gyrA*)	Fw-CGCCCGACAAACCGCATAAA Rv-CAAGGACTCATAGATTGCCGAA	95	Desroche et al., 2005		
RS00025/0005 DNA gyrase subunitB (*gyrB*)	Fw-GAGGATGTCCGAGAAGGAATTA Rv-ACCTGCTGGGCATCTGTATTG	107	Desroche et al., 2005 Margalef-Català et al., 2017		

*RNA samples were taken at signaled times, where maximum over- or under- expression had been observed in microarray assay*.

a *Microarray*.

b *RT-qPCR fold changes between: t = 0 h and t in which there is the maximum expression or inhibition after the inoculation*.

### Proteomic analysis

#### 2D-DIGE

Protein extracts were analyzed in the Center for Omic Sciences from *Servei de Recursos Científics i Tècnics* of University Rovira i Virgili (Reus, Spain). Proteins were precipitated using TCA/Acetone, and the pellet was resuspended in 200 μL of rehydration buffer (7 M urea, 2 M thiourea, 4% CHAPS, 30 mM Tris-base) at final pH 8.5. The samples were quantified using Bradford and stored at −20°C. Fifty microgram of each protein sample was minimally labeled with 400 pmol of either Cy3 or Cy5 (N-hydroxy succinimidyl ester-derivatives of the cyanine dyes). To facilitate image matching and cross-gel normalization, an internal standard was made pooling all samples and labeling with Cy2 at the same ratio (50 μg:400 pmols). Hence, two samples and the internal standard could be run in the same gel and quantified on multiple 2-DE. Labeling reactions were performed on ice and darkness during 30 min and quenched using an excess of free L-lysine.

Isoelectrofocusing (IEF) was carried out using 24 cm Immobiline Dry-strips (pH interval 4–7, nonlinear, GE Healthcare), and sample was loaded by two rehydration steps (passively for 5 h at 20°C, and actively at 50 V during 12 h in an Ettan IPGphor 3 system from GE Healthcare). IEF migration program started focusing 500 V for 7 h, ramping until 1 KV during 4 h and ramping again until 10 KV during 3 h, and finally a step maintained at 10 KV to reach 70 KVh. Strips were then equilibrated for 15 min in a 50 mM Tris–HCl (pH 8.8) 6 M urea, 30% glycerol and 2% SDS buffer, adding first 1% DTT, and in a second time supplemented with 4% iodoacetamine (Görg et al., 2004).

##### Imaging and data processing

Gels were scanned using a PharosFX™ Plus Molecular Imager and analyzed using Progenesis Same Spots Analysis Software v4.5 (Totallab). Spots displaying a ≥1 average-fold increase or decrease in abundance with a *p* < 0.05 were selected for protein identification. Features detected from non-protein sources (e.g., dust particles and dirty backgrounds) were filtered out. Picking analysis was carried loading 720 μg of the internal standard mix without labeling in a 2DE gel as described above. Gels were stained with Coomassie blue G250 and imaged using Pharos FXTM Plus Molecular Imager from BioRad using Quantity One version 4.6.9 software.

##### In gel-trypsin digestion and MS-Based protein identification

Spots of interest were automatically excised from 2-DE gels using the ExquestTM Spot Cutter with the PDQuestTM Advanced 2D Analysis Software V8.0.1 both from Bio-Rad. Excised spots were de-hydrated by extensive washings with 25 mM ammonium bicarbonate and acetonitrile. All gel pieces were incubated with 15 ng/μL sequencing-grade trypsin in 50 mM ammonium bicarbonate at pH 7.9 overnight at 37°C. Following digestion, the peptides were desalted using C18 zip-tip (Millipore), and eluted with 75% ACN + 0.1% Trifluoroacetic acid.

Peptides were spotted onto an HTP BigAnchor 384 (Bruker) target using α-cyano-4-hydroxy-cinnamic acid as matrix and were analyzed on a MALDI TOF/TOF (Ultraflextreme, Bruker Daltonics, Bremen, Germany) instrument operated in the positive ion mode. All mass spectra were calibrated externally with the Peptide Calibration Standard I from Bruker. The analyzed mass range was 600–3500 Da. MS and MS/MS analyses were performed automatically. For MS analysis, 3100 satisfactory shots were accumulated by recording 100-shot steps at 20 random positions using fuzzy control laser attenuation between 40 and 100% at initial and maximal power respectively. For MS/MS 3100 satisfactory shots were accumulated by recording 100-shot steps and 4000 for the fragment ion spectra.

MS and tandem MS/MS spectra were searched by Protein Scape v: 3.0.0 446 using MASCOT (Matrix Science Inc., MA, 2.4.0) against NCBInr database (46742655 entries) restricted to Bacteria (Eubacteria). The search parameters were set to: MS accuracy 20 ppm, MS/MS accuracy 0.5 Da, two missed cleavage by trypsin allowed, carbamidomethylation of cysteine as fixed modification and oxidation of methionine and N-terminal amino acid conversion of glu and gln to pyroglutamic acid as variable modification. Significant protein hits (for peptide mass fingerprinting *p* ≤ 0.05 and for MS/MS at least two peptides with *p* ≤ 0.05, and protein scores greater than 30) were considered significant.

#### iTRAQ labeling

##### Protein digestion and iTRAQ labeling

On a SDS-PAGE gel (12% resolving gel and 4% stacking gel) at 20 mA/gel 40 μg total protein per sample were run. The electrophoresis was stopped when the front dye had barely passed from the stacking gel into the resolving gel, and a unique concentrated band was obtained for every sample, which was stained using Coomassie Brilliant Blue G-250, excised, cut into small pieces and stored at 4°C in ultrapure water.

Protein digestion was performed according to Shevchenko et al. (1996) with minor variations as described before. Proteins were reduced using 5 mM tris(2-carboxyethyl)phosphine (TCEP) in 50 mM triethylammonium bicarbonate pH 7.9 during 1 h at 60°C and alkylated with 10 mM methyl methanethiosulfate (MMTS) in the same buffer during 30 min at room temperature. To digest the samples, they were incubated with 15.4 ng/μL sequencing-grade trypsin in 50 mM triethylammonium bicarbonate at pH 7.9 overnight at 37°C. After digestion, the peptides were extracted from gel by elution in a mixture of 50% acetonitrile 5% formic acid. Tryptic peptides were dried by SpeedVac and re-suspended in 30 μL TEAB 0.5 M at pH 8.5.

iTRAQ-8plex labeling reagents (AB SCIEX) were added to each peptide samples according manufacturer's instructions and incubated at room temperature for 120 min. Mixtures of labeled samples were washed from unreacted reagents using SCX column (Strata® SCX 55 μm, 70 Å, Phenomenex) in 10 mM phosphoric acid, 25% acetonitrile, pH 3 as binding buffer and 5% ammonium hydroxide, 25% acetonitrile for the elution. After the elution, samples were vacuum dried and re-suspended in water for the next step.

##### Sample fractionation and mass spectrometry analysis (LC-MS/MS)

Pooled peptides were separated in an Agilent 3100 OFFGEL Fractionator (Agilent Technologies, Santa Clara, CA) through 24-well IPG strips (linear gradient from pH 3–10) according to the supplier's protocol. After separation, fractions were desalted and concentrated through C18 Sep-Pak column (Waters, Bedford, MA) previously to LC-MS/MS detection.

A nano LC II coupled to an LTQ-Orbitrap Velos Pro mass analyzer, both from Thermo Scientific (Bremen, Germany), was used for peptide analysis. The chromatographic separation was achieved using a nanoLC C_18_ trap column (100 μm I.D.; 2 cm length; 5 μm particle diameter, Thermo Fisher Scientific) coupled to a nanoLC C_18_ analytical column (75 μm I.D.; 15 cm length; 3 μm particle diameter, Nikkyo Technos Co. LTD, Japan) under gradient elution conditions. Ultrapure water with 0.1% HCOOH (solvent A) and acetonitrile with 0.1% HCOOH (solvent B) was the mobile phase and the gradient consisted of 0–5% B during 5 min, 5–35% B 30 min, 35–80% B 15 min and 80–100% B 12 min, and finally is maintained at 100% B during 10 min. A flow rate of 300 nL/min was used to elute peptides for real time ionization on a nanoFlex electrospray ion source from Thermo Fisher Scientific.

MS measurements detected intact peptides in a full scan (m/z 350–2000), with the Orbitrap at FT-resolution spectrum (R = 30,000 FHMW), followed by data dependent MS/MS scan from most intense 10 parent ions with a charge state rejection of one. The signal threshold for triggering an MS/MS event was set to 10,000 counts. The low mass cutoff was set to 100 m/z. Dynamic exclusion of 30 s and activation time of 0.1 s was used. For efficient fragmentation and detection of iTRAQ reporter ions, HCD normalized collision energy of 45 was used. All fragment ions were detected in the Orbitrap (*R* = 7500 FHMW). Internal calibration was performed using the ion signal of (Si(CH3)2O)6H+ at m/z 445.120025 as a lock mass. Maximal ion accumulation time allowed on the LTQ Orbitrap was 1 s for all scan modes; automatic gain control was used to prevent over-filling of the ion trap.

##### Database searches and quantitative proteome analysis

Tandem mass spectra were extracted and charge state deconvoluted by Proteome Discoverer v1.4.0.288 (Thermo Fisher Scientific). All MS/MS samples were analyzed using Mascot (v 2.4.1.0) as search engine node. Mascot was set up to search in NCBInr database (46,742,655 entries), following the application of the restriction for Firmicutes taxonomy. Two missed cleavages were allowed for trypsin digestion, and an error of 0.80 Da for fragment ion mass and 10.0 ppm for a parent ion were tolerated. Oxidation of methionine, acetylation of N-termini and ITRAQ 8-plex modifications were specified as variable modifications, whereas methylation of cysteines was set as static modification. The false discovery rate (FDR) and protein probabilities were calculated by Target Decoy PSM Validator working between 0.01 and 0.05 for strict and relaxed, respectively. For proteins identified with only one single peptide meeting these criteria, we required the Mascot score to be at least 30, and visual verification of fragmentation spectra was done. Identified proteins were grouped by the software to minimize redundancy. For quantitative analysis centroided iTRAQ reporter ion signals were computed and only unique peptides were used for relative protein quantification. iTRAQ reporter ion intensities were normalized to sample 113, that were replicated in the two ITRAQ mixtures.

The statistical analysis was performed on Mass Profile Professional software v. 12.6 from Agilent Technologies. To find differential proteins, a paired 1-way ANOVA test was used selecting a *p* < 0.05 and fold change >1.5 as cut-off values. Principal component analysis (PCA) was used to evaluate variations in the mean quantity of spots.

#### Bioinformatic tools

On-line databases like NCBI information of each gene, Computational Biology at Oak Ridge National Laboratory (ORNL; http://compbio.ornl.gov/public/section/), DAVID database (https://david.ncifcrf.gov/), KOBAS 2.0 (KEGG Orthology Based Annotation System; http://kobas.cbi.pku.edu.cn/) (Xie et al., 2011) were used to assess all the Clusters of Orthologous Groups (COGs) described for *O. oeni* genes and proteins and metabolic pathways. We analyzed the expression data of arrays with MEV (Multi Experiment View) cluster software using Quality Threshold Clustering (QTC) tool (Heyer et al., 1999). Pearson correlation and a minimum cluster population of the number of genes representative of 10% were used and a maximum cluster diameter of 0.9. For construction of Venn diagrams, Venny (an interactive tool for comparing lists with Venn's diagrams, http://bioinfogp.cnb.csic.es/tools/venny/index.html) was used.

## Results and discussion

Functional analysis using comparative transcriptomics and proteomics can provide deeper insight into the molecular mechanisms of adaptation of *O. oeni* to wine stress conditions. The aim of this work was to evaluate which genes and proteins were affected during the adaptation period occurring before the start of MLF. The study was performed with the reference strain PSU-1 using wine-like medium (WLM) at pH 3.4 and with 12% of ethanol (v/v). Under these conditions, PSU-1 showed an adaptation period of 8 h from inoculation until the beginning of MLF. Once L-malate consumption started, MLF was successfully finished in 72 h. The viability of PSU-1 did not decrease during the adaptation period, indicating that there was no detectable cell death of the inoculated population (6.87·10^7^ ± 1.70·10^7^ CFU/mL).

### Global analysis of functions affected during acclimation to WLM

In the transcriptomic analysis 1611 expressed sequence tag (EST) were detected. Among the EST that were differently expressed during adaptation, 27 were classified as discontinued or pseudo genes in NCBI, and they were not included in the analysis. Among the analyzed genes showing significant changes along the assay, 314 were over-expressed, whereas 308 genes were down-regulated. They can be consulted in Table S1 in the Supplementary Data. It is worth noting that 52 over-expressed genes and 51 down-regulated genes were annotated as hypothetical proteins (Table S2 in the Supplementary Data).

**Figure 1:**
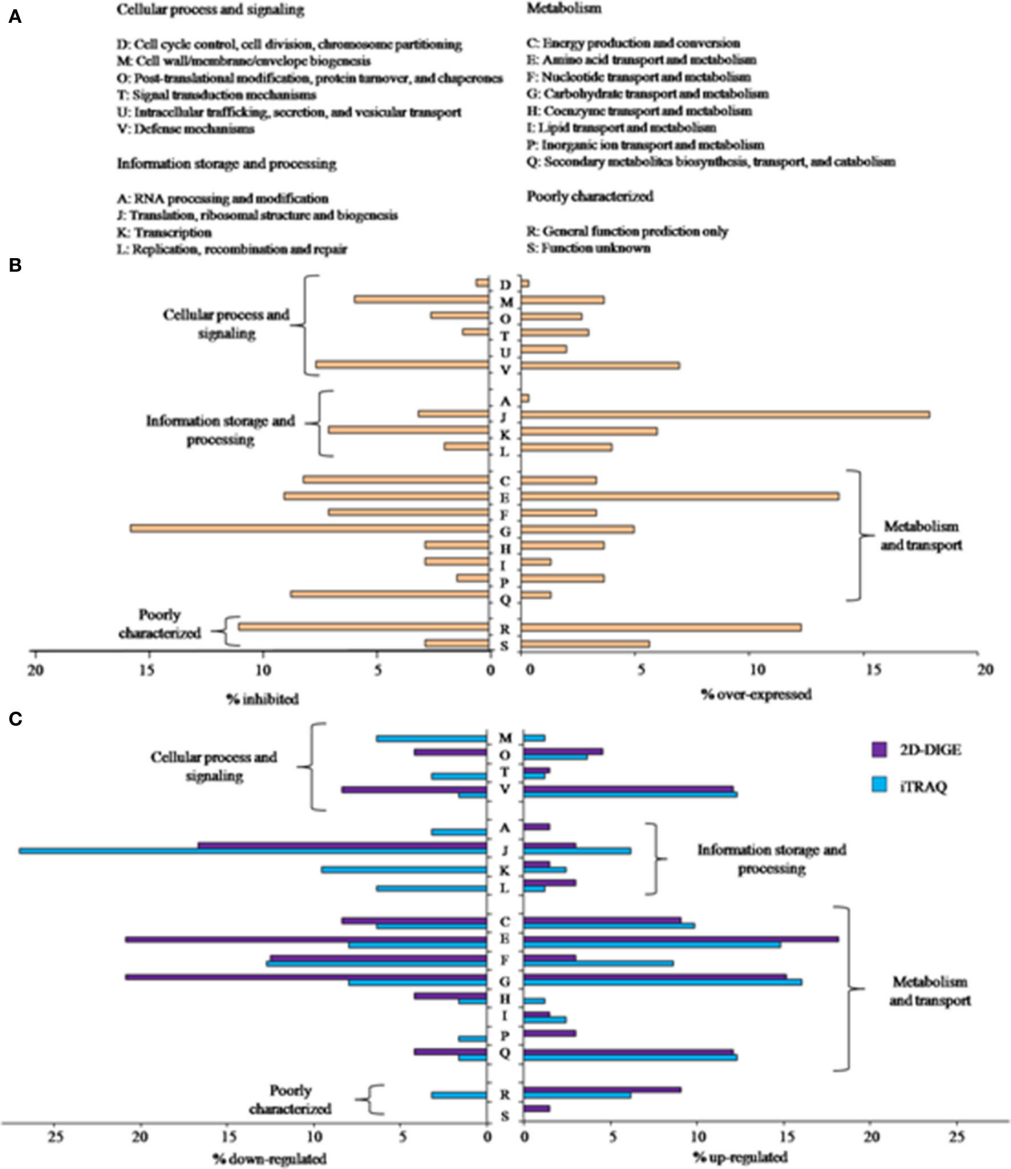
**(A)** Clusters of Orthologous Groups (COGs) definitions. **(B)** Percentage of genes of each representative COG significantly over or under-expressed according to transcriptomic analysis. **(C)** Percentage of proteins of each representative COG showing significant abundance changes, detected by 2D-DIGE or iTRAQ.

**Figure 2:**
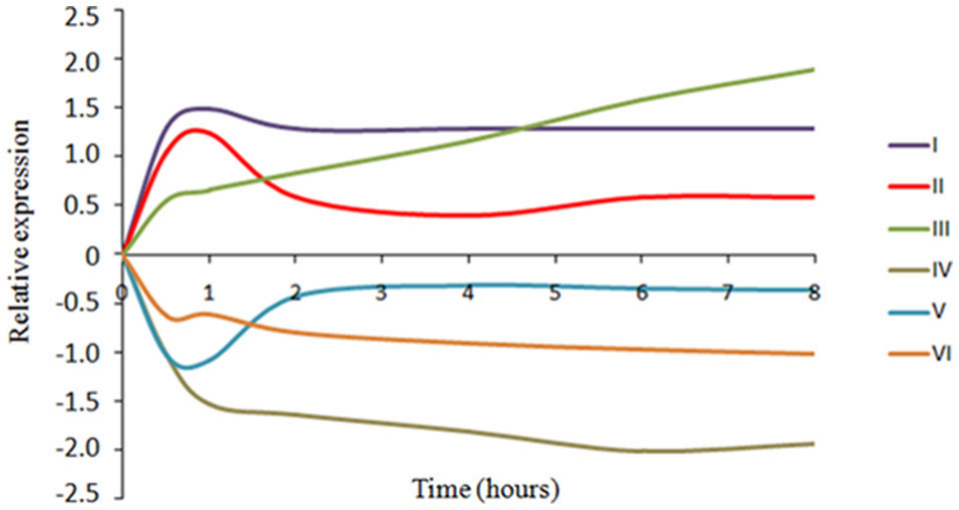
**Representative gene expression profiles according to Quality Threshold Clustering (QTC) based on transcriptomic data**. An example of each profile is shown. Profile I: OEOE_RS07930 (UDP-N-acetylmuramoyl-tripeptide-D-alanyl-D-alanine ligase); Profile II: OEOE_RS03595 (amino acid ABC transporter substrate-binding protein); Profile III: OEOE_RS05245 (oligoendopeptidase F); Profile IV: OEOE_RS07040 (glycerol-3-phosphate ABC transporter permease); Profile V: OEOE_RS03155 (F0F1 ATP synthase subunit A); Profile VI: OEOE_RS01045 (PTS sugar transporter subunit IIA).

These 622 genes differently expressed were classified in Clusters Orthologous Groups (COGs) in order to identify the main biological processes influenced by adaptation to wine conditions (Figures 1A,B). Besides, the QTC analysis grouped the differentially expressed genes into six transcriptional profiles (Figure 2). The main functions transcriptionally activated due to inoculation in WLM in *O. oeni* PSU-1 were translation, ribosomal structure and biogenesis (J) and amino acid transport and metabolism (E) (Figure 1B). The QTC analysis (Figure 2) showed two expression profiles, I and III (65.9 and 13.6% of over-expressed genes, respectively), in which translation (J) was the most represented function. These two profiles are indicative of an adaptive response since gene transcription increases progressively along adaptation process. The profile II (17.5% of over-expressed genes) included mostly genes of amino acid metabolism showing an increase in their transcription level between 0.5 and 1 h which was later decreased. This behavior is indicative of an early-response to WLM stress conditions. Regarding the genes showing an inhibited transcription, carbohydrate metabolism (G) was the main negatively regulated function in PSU-1 during adaptation to WLM (Figure 1B). The genes related to this metabolism mostly showed a constant down-regulation and were present in profile IV (Figure 2), representing 62.2% of total down-regulated genes. Fewer genes were clustered into profile V (15.3%), showing transcriptional repression only at the beginning of the assay (0.5–1 h). Finally, the profile VI grouped 19.8% of down-regulated genes which had a progressive repression during the adaptation period.

Proteomic analysis of *O. oeni* PSU-1 adaptation to WLM conditions was conducted using two techniques: 2D-DIGE and iTRAQ. The PCA analyses to evaluate the variability among samples clearly indicated the presence of three different protein populations 0, 1, and 6 h (data not shown). The 2D-DIGE results showed between 27 and 62 protein spots along the assay exhibiting differential abundance with statistical significance (*p* ≤ 0.05). The maximum differences were observed comparing the samples from 1 and 6 h after inoculation vs. 0 h. For this reason, the protein identification was carried out only for these samples and also these samples were analyzed using iTRAQ labeling in order to complement 2D-DIGE results. Using 2D-DIGE, 33 different proteins could be determined which were not found with iTRAQ. On the other hand, the off-gel technique detected 71 proteins exclusively. At 1 h there were detected more proteins up regulated than at 6 h, revealing a fast stress proteomic response of the cell against the new environment. All proteomic identification and COG classification from 2D-DIGE and iTRAQ analysis can be consulted in Table S3 (Supplementary Data).

A high percentage of the significantly down-regulated proteins (Figure 1C) belonged to the COG associated to translation, ribosomal structure and biogenesis (J). This is in accordance to previous studies that described the lower abundance of proteins involved in protein synthesis during acid stress in *Lactobacillus* species (Koponen et al., 2012; Heunis et al., 2014). On the other hand, some ribosomal proteins of 50S and 30S subunits showed an increased abundance in *O. oeni* PSU-1 after inoculation into WLM. This increase in protein concentration was coincident with an up-regulated gene transcription (Tables 2, 3). Huang et al. (2011) and Koponen et al. (2012) also described the increased abundance of 50S and/or 30S ribosomal proteins in *Lactobacillus* species as a mechanism of response to acidic stress. Therefore, certain ribosomal proteins involved in the regulation of translation may play a role in stress response as suggested by Dressaire et al. (2010).

**Table 2:** **Selection of genes related with relevant metabolisms or functions, differently regulated after inoculation into WLM from the microarray analysis**.

**Related metabolism**	**Gene annotation**	**Gene symbol**	**Relative expression**					
			**0.5 h**	**1 h**	**2 h**	**4 h**	**6 h**	**8 h**
Malate metabolism	Malate dehydrogenase	OEOE_RS02010	2.20	**2.51**	2.25	1.77	1.68	1.61
	Malate permease	OEOE_RS02015	2.25	**2.45**	2.08	1.74	1.71	1.67
	Malate transporter	OEOE_RS06985	3.28	4.03	**4.19**	3.84	3.68	3.69
Citrate metabolism	Citrate lyase	OEOE_RS02005	0.82	**1.01**	0.76	0.63	0.55	0.49
	[citrate [pro-3S]-lyase] ligase	OEOE_RS02020	1.85	**2.22**	1.77	1.23	1.20	1.17
	Citrate lyase ACP	OEOE_RS02025	1.68	**1.96**	1.88	1.44	1.24	1.45
	Citrate lyase	OEOE_RS02030	1.25	**1.50**	1.25	0.95	0.91	1.07
	Citrate lyase subunit alpha	OEOE_RS02035	0.73	**1.13**	0.93	0.59	0.54	0.64
	Acetoin reductase	OEOE_RS03325	−0.96	−1.32	−1.53	−1.80	−**1.82**	−2.01
	Diacetyl reductase	OEOE_RS07730	−0.63	−1.01	−1.25	−1.35	−**1.37**	−1.43
ATPase activity	F0F1 ATP synthase subunit A	OEOE_RS03155	−1.03	−**1.08**	−0.42	−0.31	−0.35	−0.37
	ATP synthase subunit delta	OEOE_RS03170	−0.92	−**1.16**	−0.92	−0.90	−0.91	−0.91
	ATP synthase subunit gamma	OEOE_RS03180	−**1.28**	−1.25	−0.97	−0.93	−0.84	−0.83
	F0F1 ATP synthase subunit epsilon	OEOE_RS03190	−1.35	−1.26	−1.45	−1.49	−1.56	−**1.69**
Amino acid transport and metabolism	4-aminobutyrate aminotransferase	OEOE_RS01860	2.98	**3.30**	3.27	3.20	3.21	3.30
	Amino Acid Permease	OEOE_RS01865	2.81	3.11	**3.12**	3.08	3.01	3.05
	Peptide ABC transporter permease	OEOE_RS02110	4.02	4.40	**4.48**	4.19	4.00	4.07
	Spermidine/putrescine import ATP-binding protein PotA	OEOE_RS03010	0.69	0.61	0.99	**1.27**	1.21	1.20
	Spermidine/putrescine ABC transporter permease	OEOE_RS03015	0.53	0.65	0.84	**1.14**	1.08	1.03
	Spermidine/purescine ABC transporter permease	OEOE_RS03020	0.68	0.78	0.97	**1.32**	1.28	1.31
	Carboxypeptidase	OEOE_RS04315	1.15	**1.45**	1.14	1.14	1.14	1.14
	Glutamine synthetase	OEOE_RS04565	1.88	2.08	**2.20**	2.01	1.83	1.81
	Xaa-Pro aminopeptidase	OEOE_RS04600	0.58	0.47	1.16	1.75	1.90	**1.93**
	Oligoendopeptidase F	OEOE_RS05245	0.56	0.66	0.83	1.17	1.59	**1.90**
	Spermidine/putrescine ABC transporter ATP-binding protein	OEOE_RS07070	**1.69**	1.40	1.21	1.06	0.97	0.99
	Spermidine/putrescine ABC transporter ATP-binding protein	OEOE_RS07075	**1.66**	1.16	1.01	0.92	0.91	0.86
	Spermidine/putrescine ABC transporter permease	OEOE_RS07080	**1.27**	0.75	0.71	0.51	0.41	0.49
	Amino acid permease	OEOE_RS07900	2.93	3.16	**3.18**	2.98	2.90	2.81
	Peptidase M20	OEOE_RS08295	0.89	1.14	1.08	1.35	1.49	**1.53**
	Aspartate carbamoyltransferase	OEOE_RS01235	−1.14	−1.75	−2.00	−2.27	−**2.56**	−2.50
Carbohydrate transport and metabolism	Mannose-6-phosphate isomerase	OEOE_RS00125	−2.56	−2.56	−2.56	−2.56	−2.58	−2.81
	Phosphoglyceromutase	OEOE_RS00565	−1.37	−**1.60**	−1.51	−1.40	−1.37	−1.42
	Sugar phosphate isomerase	OEOE_RS00595	−1.71	−1.98	−**2.05**	−1.72	−1.58	−1.71
	6-phospho-beta-glucosidase	OEOE_RS01060	−1.04	−1.23	−1.58	−1.81	−**2.02**	−1.94
	PTS fructose transporter subunit IIA	OEOE_RS01110	−1.85	−2.20	−2.60	−**2.90**	−2.69	−2.79
	PTS mannose transporter subunit IIAB	OEOE_RS02230	−0.73	−1.02	−1.31	−**1.38**	−1.06	−1.00
	PTS mannose transporter subunit IID	OEOE_RS02240	−0.76	−1.08	−**1.43**	−1.43	−1.23	−0.97
	Phosphocarrier protein hpr	OEOE_RS03075	−2.19	−2.16	−2.20	−**2.33**	−2.25	−2.32
	Lactate dehydrogenase	OEOE_RS05695	−1.20	−0.93	−1.10	−1.22	−1.14	−**1.37**
	PTS sugar transporter	OEOE_RS05805	−2.37	−2.45	−2.68	−2.86	−2.87	−**2.90**
	Glycerol-3-phosphate ABC transporter ATP-binding protein	OEOE_RS07030	−1.42	−1.92	−2.10	−2.34	−2.51	−**2.54**
	Glycerol-3-phosphate ABC transporter permease	OEOE_RS07035	−0.90	−1.36	−1.53	−1.65	−1.72	−**1.76**
	Glycerol-3-phosphate ABC transporter permease	OEOE_RS07040	−1.03	−1.54	−1.64	−1.81	−**2.01**	−1.93
	Glycerol-3-phosphate ABC transporter substrate-binding protein	OEOE_RS07045	−1.16	−1.47	−1.86	−2.13	−2.20	−**2.22**
	UDP-phosphate galactose phosphotransferase	OEOE_RS07255	−1.98	−1.96	−1.96	−**2.01**	−1.94	−1.96
	Ribokinase	OEOE_RS07775	−1.53	−1.87	−**2.32**	−2.18	−1.64	−1.57
	D-ribose pyranase	OEOE_RS07780	−1.27	−1.82	−**2.09**	−2.08	−1.59	−1.33
	Sugar:proton symporter	OEOE_RS07785	−1.28	−1.75	−**2.22**	−2.19	−1.73	−1.56
	Enolase	OEOE_RS07960	−0.76	−0.76	−0.75	−1.06	−1.07	−**1.12**
	Sugar phosphate isomerase	OEOE_RS08055	−1.35	−1.33	−1.79	−2.11	−2.02	−**2.24**
	Fructokinase	OEOE_RS0824	−1.42	−2.08	−2.15	−2.28	−2.40	−**2.46**
Lipid transport and metabolism	Tannase	OEOE_RS05040	1.88	2.26	**2.72**	2.22	1.89	1.90
	Cyclopropane-fatty-acyl-phospholipid synthase	OEOE_RS05660	−0.90	−**1.11**	−0.98	−0.90	−0.86	−0.83
	Glycerophosphoryl diester phosphodiesterase	OEOE_RS07050	−1.49	−1.64	−2.08	−2.51	−**2.57**	−2.56
Cell wall/membrane/envelope biogenesis	Glucosamine–fructose-6-phosphate aminotransferase	OEOE_RS03035	2.94	3.14	3.65	3.92	**3.99**	3.97
	D-alanyl-D-alanine carboxypeptidase	OEOE_RS03435	5.53	5.83	**6.01**	5.82	5.61	5.64
	Peptidoglycan interpeptide bridge formation protein	OEOE_RS06965	0.95	1.87	**2.52**	2.39	1.85	1.70
	Sortase	OEOE_RS06970	1.46	2.00	**2.48**	2.27	1.94	1.91
	Peptidoglycan interpeptide bridge formation protein	OEOE_RS06975	1.55	2.27	**2.77**	2.52	2.24	2.19
	Glycosyl transferase	OEOE_RS07820	1.78	1.90	2.86	**3.39**	3.35	3.08
	D-alanyl-D-alanine carboxypeptidase	OEOE_RS07530	−0.96	−1.45	−1.49	−1.60	−**1.75**	−1.70
Translation, ribosomal structure and biogenesis	Serine–tRNA ligase	OEOE_RS02120	1.44	1.93	**2.07**	1.93	1.77	1.72
	Elongation factor 3	OEOE_RS02460	1.48	1.97	**2.28**	1.95	1.83	1.82
	30S ribosomal protein S10	OEOE_RS02840	1.88	1.82	1.88	1.92	**1.93**	1.90
	30S ribosomal protein S8	OEOE_RS02910	2.07	2.04	2.35	**2.57**	2.45	2.42
	50S ribosomal protein L15	OEOE_RS02935	1.00	1.18	1.41	**1.70**	1.64	1.63
	50S ribosomal protein L17	OEOE_RS02970	0.56	0.93	1.41	1.75	**1.83**	1.75
	50S ribosomal protein L32	OEOE_RS03680	−0.14	0.59	0.78	0.87	0.88	**1.07**
	Acetyltransferase	OEOE_RS04010	2.37	2.70	2.99	**3.18**	3.07	2.77
	30S ribosomal protein S20	OEOE_RS06185	0.29	0.84	1.07	1.29	**1.32**	0.83
	50S ribosomal protein L7/L12	OEOE_RS06825	0.63	1.16	1.71	**2.00**	1.74	1.46
Stress response	Ferredoxin–NADP reductase	OEOE_RS00770	0.93	1.14	1.24	1.35	**1.39**	1.35
	Multidrug ABC transporter ATP-binding protein	OEOE_RS02115	3.69	4.43	**4.75**	4.45	4.26	4.08
	Thioredoxin reductase	OEOE_RS02695	1.06	**1.66**	1.60	1.23	1.20	1.23
	N-acetylmuramoyl-L-alanine amidase	OEOE_RS02805	1.85	2.06	**2.14**	2.01	1.79	1.71
	Acetyl esterase	OEOE_RS03440	3.23	3.72	**4.21**	4.03	3.69	3.61
	Multidrug ABC transporter atpase	OEOE_RS03445	1.72	1.69	2.02	**2.16**	1.93	2.02
	Multidrug ABC transporter permease	OEOE_RS03450	2.15	2.01	2.34	**2.48**	2.35	2.18
	Multidrug ABC transporter permease	OEOE_RS03640	0.88	**1.23**	1.10	1.03	1.06	1.17
	Multidrug MFS transporter	OEOE_RS04200	2.39	2.77	3.14	3.51	3.73	**3.75**
	Molecular chaperone dnaj	OEOE_RS06305	1.56	**1.76**	1.61	1.36	1.32	1.10
	Molecular chaperone dnak	OEOE_RS06310	1.03	1.35	**1.38**	1.04	1.03	0.95
	Protein GrpE	OEOE_RS06315	0.93	1.41	**1.59**	1.11	1.02	0.94
	Multidrug ABC transporter ATP-binding protein	OEOE_RS07890	**1.07**	0.93	0.72	0.69	0.59	0.65
	Peptidylprolyl isomerase	OEOE_RS07905	2.23	**2.74**	2.62	2.30	2.10	1.97
	Multidrug ABC transporter ATP-binding protein	OEOE_RS08260	1.48	1.54	1.68	**1.80**	1.47	1.05
	Multidrug ABC transporter ATP-binding protein	OEOE_RS08265	1.01	1.04	1.25	**1.40**	1.10	0.76
	General stress protein	OEOE_RS00325	−2.18	−2.10	−2.46	−2.54	−**2.60**	−2.58
	Glutaredoxin	OEOE_RS00645	−1.04	−0.44	−0.27	−0.64	−0.82	−0.86
	Heat-shock protein Hsp20	OEOE_RS01385	−0.92	−0.43	−0.55	−0.89	−0.88	−**1.13**
	Glutathione reductase	OEOE_RS05740	−0.63	−0.78	−0.83	−0.96	−**1.00**	−0.98
	Cold-shock protein	OEOE_RS06620	−1.75	−1.14	−1.30	−1.49	−1.73	−**1.81**
	Thiol-disulfide isomerase	OEOE_RS07835	−1.17	−**1.32**	−1.18	−1.03	−1.01	−1.07
	Thioredoxin	OEOE_RS08215	−**1.10**	−0.71	−0.71	−0.70	−0.68	−0.81
Nucleotide transport and metabolism	Adenylate kinase	OEOE_RS02945	1.21	1.08	1.22	1.36	**1.44**	1.22
	Deoxyadenosine kinase	OEOE_RS04085	−1.23	−**1.25**	−1.08	−1.07	−1.13	−1.24
Coenzyme transport and metabolism	Thiamine pyrophosphokinase	OEOE_RS03790	−0.93	−**1.07**	−1.05	−0.96	−0.93	−0.84
	Pyridoxal biosynthesis protein	OEOE_RS04980	−0.89	−1.10	−1.22	−1.19	−**1.23**	−1.15

*Genes which regulation is coincident with proteomic results are gray highlighted. For each gene, time sample with maximum over- or under-expression is bold highlighted*.

**Table 3:** **Selection of relevant proteins detected by 2D-DIGE and iTRAQ analysis differently regulated after WLM inoculation at 1 and 6 h**.

**Related metabolism**	**Protein annotation**	**Gene symbol**	**Fold change**				**Theoretical Mr (KDa)**	**Pi**
			**DIGE**		**iTRAQ**		**DIGE/iTRAQ**	**DIGE/iTRAQ**
			**1 h**	**6 h**	**1 h**	**6 h**		
Malate metabolism	Malate dehydrogenase	OEOE_RS02010	−	−	−1	−0.8	41.4	
Citrate metabolism	Acetoin reductase	OEOE_RS03325	−	−	−1.7	−0.9	27.4	
	Diacetyl reductase	OEOE_RS07730	ND	1.237	−	−	27.4	5.26
			ND	1.626	−	−	27.4	5
ATPase activity	F0F1 ATP synthase subunit alpha	OEOE_RS03175	−	−	−1.3	0.7	56.7	
Amino acid transport and metabolism	Aspartate carbamoyltransferase	OEOE_RS01235	−1.31	1.53 (2)	−	−	35/38.9	6.11
	Aminopeptidase C	OEOE_RS02220	ND	−1.1	−	−	50.5	5.2
	Dipeptidase	OEOE_RS02735	−	−	1.1	−0.8	41.2	
	Glutamine synthetase	OEOE_RS04565	−	−	0.6	1	49.9	
	Peptidase M20	OEOE_RS04760	1.3	1.36	1.3	0.7	44.1/42.1	4.4
	Glutamine amidotransferase	OEOE_RS04955	−	−	−1	0.7	27.4	
	Aminopeptidase N	OEOE_RS05080	ND	−2.14	0.9	−1.1	95.1	5.1
	Succinate-semialdehyde dehdyrogenase	OEOE_RS06260	−	−	1.6	1.1	51.5	
	S-ribosylhomocysteine lyase	OEOE_RS07535	1.51 (3)	1.57 (2)	1.8	1.5	17.7	5.3
	Peptidase C69	OEOE_RS08595	−	−	1.9	0.9	53.5	
Carbohydrate transport and metabolism	Phosphoglycerate mutase	OEOE_RS00565	−1.57	−1.53	−	−	27.1	5.4
	Aldehyde dehydrogenase	OEOE_RS01550	1.21 (3)	1.59 (2)	−	−	52.5	4.9
	Lactate dehydrogenase	OEOE_RS01985	−	−	1.5	1.1	36.5	
	PTS mannose transporter subunit IIAB	OEOE_RS02230	−0.73	−1.02	−1.31	−1.38	−1.06	−1.00
	PTS mannose transporter subunit EIIAB	OEOE_RS02230	−	−	−2.4	−0.7	35.6	
	HPr kinase/phosphorylase	OEOE_RS02680	1.6	1.9	−	−	35.4	5.3
	Phosphocarrier protein HPr	OEOE_RS03075	−	−	−0.7	2.3	9.0	
	Phosphoenol pyruvate-protein phosphotransferase	OEOE_RS03095	−1.2	ND	−	−	63.2	5
	Galactose mutarotase	OEOE_RS04920	−	−	0.7	−1.1	34.1	
	D-lactate dehydrogenase	OEOE_RS05695	−1.3	ND	−	−	36.5	5.74
	UDP-glucose 4-epimerase	OEOE_RS06755	−	−	1.1	0.7	36.9	
	Enolase	OEOE_RS07960	1.54	1.387	0.7	1.8	48.4/47.3	4.6
	Fructokinase	OEOE_RS08245	−1.2	ND	−	−	32.1	6.44
Lipid transport and metabolism	ACP S-malonyltransferase	OEOE_RS07670	−	−	−0.8	1.1	33.6	
	2-nitropropane dioxygenase	OEOE_RS07675	−	−	−1.5	0.7	33.5	
Cell wall/membrane/envelope biogenesis	Glucosamine–fructose-6-phosphate aminotransferase	OEOE_RS03035	−	−	1.7	1.1	66.2	
	Rod shape-determining protein MreB	OEOE_RS03200	−	−	−1.2	−1.1	40.1	
	UDP-N-acetylmuramate–L-alanine ligase	OEOE_RS06110	−	−	0.6	−1.6	48.1	
	Peptidoglycan interpeptide bridge formation protein	OEOE_RS06975	−	−	−1.1	−1	39.4	
	D-alanyl-D-alanine carboxypeptidase	OEOE_RS07530	−	−	−0.8	−1.5	31.0	
	UDP-N-acetylglucosamine 1-carboxyvinyltransferase	OEOE_RS08605	−	−	0.7	1	45.7	
Translation, ribosomal structure and biogenesis	Threonyl-tRNA synthase	OEOE_RS02215	−2.02	−3.3	−0.7	−1.1	76.3	
	30S ribosomal protein S8	OEOE_RS02910	−	−	−1.5	1	14.6	
	50S ribosomal protein L15	OEOE_RS02935	−	−	0.7	2.3	16.5	
	50S ribosomal protein L17	OEOE_RS02970	−	−	−0.7	1.2	14.9	
	50S ribosomal protein L32	OEOE_RS03680	−	−	0.7	1.2	6.7	
	Elongation factor Tu	OEOE_RS03795	−1.3	−1.6	−1.6	−0.9	43.6	4.9
	Elongation factor Ts	OEOE_RS04685	−	−	−2.3	−0.9	31.8	
	30S ribosomal protein S20	OEOE_RS06185	−	−	0.7	1	9.8	
	Elongation factor G (fusA)	OEOE_RS06335	−1.67	1.015	−	−	77.9	
	Valyl-tRNA synthase	OEOE_RS06700	−	−	0.8	−1.9	104.6	
	50S ribosomal protein L7/L12 (rplL)	OEOE_RS06825	ND	1.99	−	−	12.2	4.20
	Methionyl-tRNA synthetase	OEOE_RS08425	−	−	−0.7	−1.1	77.0	
Stress response	Glutathione reductase	OEOE_RS05740	−	−	2	1	48.6	
	Cold-shock protein	OEOE_RS06620	1.28	ND	−	−	7.4	4.7
	Molecular chaperone GroEL	OEOE_RS06725	1.1	1.35 (2)	−	−	57.5	5.02
	Co-chaperonin GroES (HSP10)	OEOE_RS06730	1.67	1.82 (2)	−	−	9.7	4.7
	Thiol reductase thioredoxin	OEOE_RS07835	−	−	−0.6	1.4	12.6	
	Thiol reductase thioredoxin	OEOE_RS08215	−	−	−0.7	1.3	11.5	
	DNA-binding ferritin-like protein	OEOE_RS08440	1.64	1.78	−	−	18.3	4.4
Nucleotide transport and metabolism	Adenylate kinase	OEOE_RS02945	−	−	−0.9	1.1	20.7	
	Deoxynucleoside kinase	OEOE_RS04085	−2.4	−2.7	−	−	25.8	5.6
Coenzyme transport and metabolism	Thiamine pyrophosphokinase	OEOE_RS03790	−2.1	ND	−	−	25.4	4.7
	Pyridoxal biosynthesis lyase PdxS	OEOE_RS04980	−	−	−2.5	1	31.4	

*Proteins which regulation is coincident with transcriptomic results are gray highlighted*.

A relevant number of proteins related to amino acid and carbohydrate metabolism (E, G) showed significant variations in abundance (Figure 1C) both increasing or decreasing, being some of them in accordance to the transcriptional response (Tables 2, 3). On the other hand, detected proteins related to defense mechanism (V) and secondary metabolites (Q) showed mostly an increased abundance (Figure 1C).

### Main metabolisms modified by wine-like conditions

#### Malate and citrate metabolism

Three out of the five malate related genes annotated in *O. oeni* PSU-1 genome were over-expressed: one of the permeases (*mleP*), the transporter OEOE_RS06985 -which had a 4-fold expression at 1 h after the inoculation-, and the malate dehydrogenase (*mae*) (Table 2). The observed transcriptional activation of malate transporters under wine-related conditions were in accordance with previous studies (Labarre et al., 1996) and were indicative of the induction of MLF as a part of stress response. In Augagneur et al. (2007) a significant increase was observed in the abundance of mRNA encoding MleP derived from cells incubated in presence of L-malate at pH 4.5 and 3.2. Similarly, *mleP* was over-expressed in the microarray performed by Costantini et al. (2015) due to adaptation (1 day) to ethanol 8 and 12%.

In this work it was detected the over-expression of the citrate lyase operon, observing the highest expression 1 h after the inoculation into WLM (Table 2). The transcriptional activation of *O. oeni* citrate lyase in response to ethanol stress has been previously reported by Olguín et al. (2009). The transcriptional up-regulation of malate transport and citrate consumption could be indicative of the use of L-malate and citrate associated to stress response and as an alternative energy source to sugar metabolism. Significant changes were also observed for genes involved in diacetyl utilization. Diacetyl is the main aromatic compound associated to MLF and is derived from citrate consumption. Diacetyl reductase showed transcriptional inhibition, while its protein abundance increased 6 h after inoculation into WLM. On the other hand, acetoin reductase was inhibited both at gene and protein level. Diacetyl and acetoin reductases are involved in two reactions of transformation of diacetyl, first into acetoin and then into 2,3-butanediol as the final product. These two reactions involve the oxidation of NAD(P)H and would participate in the maintenance of the cofactor redox balance. Diacetyl metabolism has been described as strain-dependent (Bartowsky and Henschke, 2004). In the studied conditions with PSU-1 strain, diacetyl and acetoin reductases would be initially inhibited, however proteomic data showed the increase in abundance of diacetyl reductase toward the beginning of MLF, which could be correlated to the activation of citrate consumption and the consequent production of diacetyl.

#### ATPase activity

ATPase activity has been associated to MLF (Salema et al., 1996). Cox and Henick-Kling (1989) proposed a chemiosmotic mechanism where energy is produced by the efflux of L-lactate from L-malate degradation. Fortier et al. (2003) described the increase of F_0_F_1_-ATPase β subunit mRNA in response to low pH. However, in this work several genes codifying for other ATPase subunits (α, δ, γ, and ε) were down-regulated before the beginning of MLF (Table 2). Although, in the proteomic study the subunit α was initially down-regulated (1 h), its abundance increased at 6 h. This could indicate that when cells are longer acclimated to WLM (6 h after inoculation), and closer to the start of L-malate consumption, ATPase activity is increased.

#### Amino acid transport and metabolism

It is worth to note the activation of several genes related with peptidase activity and amino acid transport (Table 2). Also, five peptidases were identified in the proteomic analysis, but their abundance varies depending on the protein and the analyzed time. Liu et al. (2010) reported that many of the peptidases seem to be essential for bacterial growth or survival as they are encoded in all LAB genomes, such as PepC, PepN, and PepM, and proline peptidases PepX and PepQ. Currently in PSU-1 genome there are annotated 29 peptidases. Also, related to nitrogen uptake, three permeases involved in nitrogen compounds transport are strongly over-expressed (Table 2) after the inoculation into WLM, reaching the maximum activation at 2 h. Since peptides account for the largest proportion of total nitrogen in wine (Feuillat et al., 1998), these results suggest the relevance of wine nitrogen composition and the ability of *O. oeni* to cope with its environment as reported by Manca de Nadra et al. (1999) and Ritt et al. (2008).

Some genes and proteins related to glutamine and glutamate synthesis, involved in the assimilation and re-distribution of nitrogen within the cell, were up-regulated revealing the key role of nitrogen uptake for *O. oeni* in a poor nutrient media, such as WLM. Glutamine synthetase was up-regulated both at gene and protein level. The 4-aminobutyrate aminotransferase gene (OEOE_RS01860), which transforms GABA into succinate semialdehyde and L-glutamate, was threefold over-expressed during the 8 h of *O. oeni* PSU-1 adaptation to WLM. GABA can be assimilated as a nitrogen and/or carbon source in bacteria such as *Escherichia coli* (Bartsch et al., 1990) and *Corynebacterium glutamicum* (Zhao et al., 2012), but no information is available about LAB in this respect.

Six genes involved in the transport of spermidine/putrescine were over-expressed (Table 2). The uptake of these two polyamines has been associated with an energy-producing state/membrane potential of the cell in *E. coli* (Kashiwagi et al., 1997). Both putrescine and spermidine protect against oxidative stress (Tkachenko et al., 2001). Olguín et al. (2015) reported that this protective mechanism may also be a target of ethanol damage in an ethanol shock, which would inhibit the uptake of these polyamines. In this case, the adaptation to conditions of WLM resulted in an over-expression of six out of the eight transporters of these polyamines annotated in PSU-1 genome.

#### Carbohydrate transport and metabolism

Microarray data revealed that sugar transport was repressed (Table 2) in response to WLM conditions. In particular, glycerol-3-phosphate ATP-binding cassette ABC transporters and mannose phosphotransferase transporters (PTS) were down-regulated. This inhibition is probably due to the lower availability of sugars in WLM with respect to rich growth medium in which inocula were prepared. A strong transcriptional inhibition of sugar metabolism and transport in response to ethanol was also observed by Olguín et al. (2015).

Enolase, among others, were strongly up-regulated at low pH compared with the optimal growth at pH 6.8 (Lee et al., 2008). In this assay, the enolase protein increased in abundance 1 h after inoculation. On the contrary, the transcriptomic data reported in this work and by Costantini et al. (2015) show the inhibition of enolase gene under wine-like conditions. This suggests that enolase, might be up-regulated at translational level in response to stress. This protein, besides being involved in sugar fermentation, has been related to host tissue adhesion in probiotic bacteria including *Lactobacillus plantarum* (Castaldo et al., 2009).

#### Lipid transport and metabolism

The regulation at gene and protein level of lipid metabolism was scarce and, in most of the cases, was down-regulated. For instance, the gene *cfa* (OEOE_RS05660) which is involved in the conversion of monounsaturated fatty acids to cyclopropane fatty acids (CFAs) was inhibited at 1 h. The increase in *cfa* transcription was observed in acid- and ethanol-grown cells (Grandvalet et al., 2008; Olguín et al., 2010) after a longer period of stress exposure than in this work, once MLF had started.

#### Cell wall/membrane/envelope biogenesis

Several genes and proteins related to cell envelope biogenesis were over-expressed. One of the genes annotated as D-alanyl-D-alanine carboxypeptidase (OEOE_RS03435) was 6-fold over-expressed at 2 h and its sharp over-expression started 0.5 h after the inoculation into WLM. This gene and OEOE_RS06975 (for peptidoglycan interpeptide bridge formation protein) were also over-expressed in transcriptomic analysis by Costantini et al. (2015), both after adaptation with 12% of ethanol. However, in the present study, another D-alanyl-D-alanine carboxypeptidase (OEOE_RS07530) showed an opposite behavior, being down-regulated both at gene and protein level. Peptidoglycan (PG) is an essential component of the bacterial cell envelope, required for cell shape and stability (Vollmer et al., 2008). A supply of D-amino acids is essential for peptidoglycan synthesis; moreover, D-Ala is the main constituent of wall teichoic acids and lipoteichoic acids, which are polyanionic polymers exclusively found in Gram-positive bacteria (Wecke et al., 2009). Another activated function, both at gene and protein level, was glucosamine:fructose-6-phosphate aminotransferase, which is also involved in cell wall biosynthesis. Cecconi et al. (2009) found a major concentration of glucosamine 6-phosphate aminotransferase in *O. oeni* cells acclimated to ethanol than in not acclimated cells. These results point out the specificity and relevance of some enzymes involved in cell envelope protection against stress challenge.

Three proteins related to cell wall biogenesis were down-regulated. Among them, the rod shape-determining protein (MreB), with an actin-like role, was less abundant at 1 and 6 h. These results correlate with the reported information for proteins MreB1 and B2 determining cell shape from *L. plantarum* 423 which were less abundant in acid-stressed cells (Heunis et al., 2014) and the transcriptional inhibition due to ethanol of other rod shape-determining proteins, such as MreB, in *O. oeni* (Olguín et al., 2015). Also, microarray analysis revealed the inhibition of three genes (OEOE_RS07265, OEOE_RS03340, and OEOE_RS07010) involved in cell-wall biogenesis, encoding one of them a capsular polysaccharide biosynthesis protein, gene which was described as well by Dimopoulou et al. (2012) as *wzd*. All these down-regulated functions would be targets of the damage caused by factors such as ethanol and low pH.

#### Translation, ribosomal structure, and biogenesis

One of the categories showing more significant changes in *O. oeni* PSU-1 transcriptome and proteome due to WLM conditions was translation related functions. It is worth noting that several 30S and 50S ribosomal genes were over-expressed like their correspondent proteins. Several ribosomal proteins were up-regulated in *Lactobacillus rhamnosus* under acidic stress (Koponen et al., 2012). Moreover, in *O. oeni* in agreement with these observations, Cecconi et al. (2009) reported that adaptation in half strength wine-like medium correlates with the up regulation of some transcription/translation proteins as elongation factor Ts and ribosomal protein 30S. A gene codifying for a ribosomal protein was differentially over expressed between 0 and 1 h after ethanol addition in *O. oeni* PSU-1 (Olguín et al., 2015). As well in samples of *O. oeni* adapted to 12% ethanol, a ribosomal protein was up-regulated (Costantini et al., 2015). Studies with *Lactococcus lactis* suggest that the regulation of translation has a major role in stress response (Dressaire et al., 2010). According to our results this would also happen in *O. oeni*.

#### Stress response

As expected, it was observed the activation of chaperon function in response to WLM stress conditions. Some genes, such as *grpE, dnaJ*, and *dnaK*, and proteins, like GroEL and GroES (Hsp10), showed up-regulation in PSU-1 after inoculation into WLM (Tables 2, 3). The latter chaperone, Hsp10, is conserved along LAB (Sugimoto et al., 2008). However, Hsp20, the most characterized stress protein in *O. oeni* (Guzzo et al., 1997, 2000), showed transcriptional inhibition in our assay and no changes in protein concentration. This is in accordance with Costantini et al. (2015), that described the transcriptional activation of *hsp20* only in mild ethanol stress (8%) but the under-expression of this gene with 12% ethanol, as found in our work. A cold shock protein (OEOE_RS06620) showed increased abundance 1 h after *O. oeni* PSU-1 inoculation, but its gene expression was inhibited along the assay. This protein could play a role in the early response to wine-related stress but not in the long term adaptation process.

Our data revealed that wine-like conditions caused an increase of proteins involved in oxidative stress protection, related to thioredoxin and glutathione systems. Two out of the three thioredoxins (*trxA*) annotated for PSU-1, OEOE_RS07835, and OEOE_RS08215, were up-regulated 6 h after inoculation. Also thioredoxin reductase (*trxB*), OEOE_RS02695, and a ferredoxin reductase gene (*fdr*: OEOE_RS00770), annotated in NCBI as *trxB* until February 2015, were activated under wine stress conditions. Glutathione reductase (GshR) was significantly more abundant in *O. oeni* PSU-1 after inoculation into WLM. However, transcriptomic data revealed that some of these genes were inhibited; indicating that translational regulation of these functions would be prevalent under the studied conditions. Our results support the relevance of the thioredoxin and glutathione systems in the adaptation of *O. oeni* to wine related stress. There are few studies regarding thioredoxin in *O. oeni* (Jobin et al., 1999a; Guzzo et al., 2000; Margalef-Català et al., 2017), thus the role of this mechanism and glutathione system against wine stress is quite unknown.

Among the genes over-expressed related to defense mechanism there were eight multidrug transport genes. ABC transporters are a major part of the efflux systems involved in the transport of harmful-compounds and cell detoxification (Leverrier et al., 2004).

### Evaluation of omic data correlation

#### Real-Time qPCR validation

In order to validate the results obtained from the microarray analysis, real-time qPCR was performed with the same RNA from the original microarray experiment. Twenty-two genes, some related to stress response, were selected, taking the RNA sample of time where maximum over- or under- expression had been observed in microarray (Table 1). There was a general accordance between microarray and real-time qPCR data for all the genes tested. Of the 22 genes, 17 were clearly correlated using both techniques. For *hsp18* gene higher values by qPCR were obtained than for microarray data. Finally the four remaining genes (diacylglycerol kinase, PTS sugar, *cfa* and *trxA2*) displayed lower numerical values by qPCR, indicating no significant changes using this technique, while with microarray they were slightly inhibited. Overall, the correlation between real-time qPCR and microarray was good, suggesting that the microarray gene expression measurements were valid. Moreover, the validation of two thioredoxins (*trxA2* and *trxA3*) was useful for the proteomic identification.

#### Integration of transcriptomic and proteomic analysis

**Figure 3:**
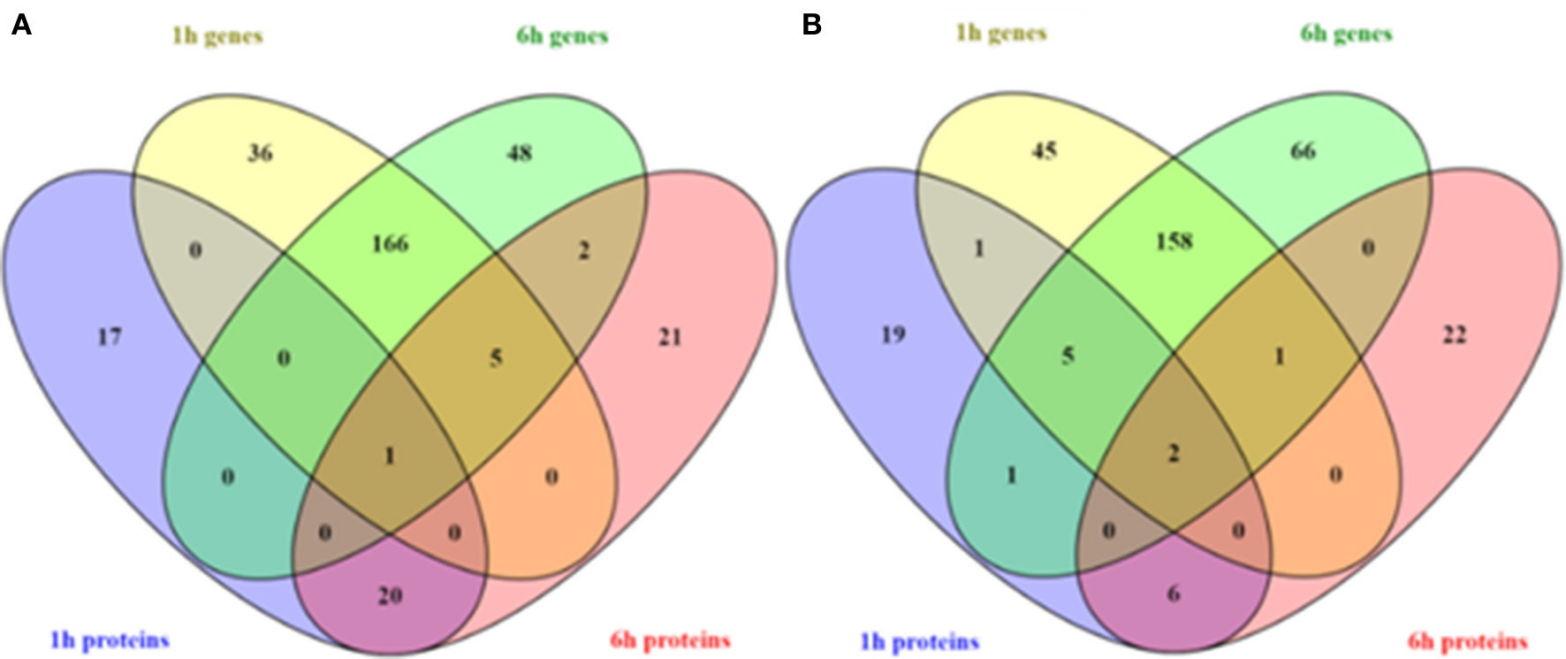
**Venn diagram of the number of proteins and genes showing significant changes in abundance and expression, respectively, according to transcriptomic, and proteomic analysis 1 and 6 h after the inoculation of ***O. oeni*** PSU-1 into WLM. (A)** over-expressed genes and up-regulated proteins, **(B)** under-expressed genes and down-regulated protein. The color of diagram petals match with the colored legends in the figure.

It has been largely reported that the correspondence of transcriptomic and proteomic data is low due to the numerous and complex regulatory mechanisms involved in gene transcription and protein synthesis (Dressaire et al., 2010; Haider and Pal, 2013). In this work, 19 genes presented a correlation with proteomic results (Tables 2, 3). The most relevant, in terms of understanding *O. oeni* stress response, have already been discussed in the text. Venn diagram shown in Figure 3 shows the number of coincident modifications of genes and proteins at different analyzed times vs. time zero. It is worth to note the highest number of coincidences was observed for genes up/down regulated (166 and 158, respectively) 1 and 6 h after inoculation in WLM. This indicates that most of the transcriptional changes were sustained along the 8 h assay, before MLF start. However, some genes were only modified at one of the analyzed times, indicating its specific role in early (1 h) or adaptive (6 h) response, respectively. Regarding protein changes, the observed pattern was different and many proteins showed modifications only at one of the two analyzed times. However, some proteins maintained the up or down regulation along the assay. Altogether, the data reported illustrates the complexity of *O. oeni* cell regulation and the difficulty of finding specific marker genes and/or proteins associated to stress response.

## Conclusions

The combined transcriptomic and proteomic study was useful to identify the metabolisms mostly altered due to wine-like conditions. The use of two complementary proteomic techniques allowed the detection of a major number of proteins influenced by stress factors. Our results revealed the relevance of translation regulation and nitrogen uptake as key metabolisms involved in the adaptation of *O. oeni* PSU-1 to wine related stress. Cell wall biosynthesis and redox maintenance mechanisms seem to play also a relevant role in the protection of *O. oeni* against cell damage. Finally, sugar metabolism is inhibited in contrast to the transcriptional activation of L-malate transport and citrate consumption before the beginning of MLF.

Most of the molecular modifications occurring during *O. oeni* adaptation to wine will depend on the strain and/or fermentation conditions. However, the *omic* analysis allows the identification of the most relevant functions affected by wine-related stress, on which should be focused future research.

## Author contributions

MM, IA: performed the experiments, participated in the acquisition, analysis and interpretation of the data, approved the final version of the paper. AB, CR, JB: supervised the laboratory work, participated in the analysis and interpretation of the data, drafted the manuscript, and approved the final version of the paper.

### Conflict of interest statement

The authors declare that the research was conducted in the absence of any commercial or financial relationships that could be construed as a potential conflict of interest. The reviewer EET and handling Editor declared their shared affiliation, and the handling Editor states that the process nevertheless met the standards of a fair and objective review.
