# Supplementary material for: Transcriptomic and Proteomic Analysis of Oenococcus oeni Adaptation to Wine Stress Conditions
Source: Front Microbiol. 2016 Sep 30;7:1554. doi: 10.3389/fmicb.2016.01554 (PMC5044463; doi:10.3389/fmicb.2016.01554)
Supplement: Supplementary file 1 [file Table1.DOCX]

**Table S1.** Relative expression of genes affected during acclimation to WLM, grouped by Clusters of Orthologous Groups (COGs). Time samples with over- or under-expression are highlighted (green and yellow, respectively).

| **COGs** | **Old locus tag** | **Gene symbol** | **Gene annotation** | **Relative expression in function of time (h)** | | | | | |
| --- | --- | --- | --- | --- | --- | --- | --- | --- | --- |
|  |  |  |  | **0.5** | **1** | **2** | **4** | **6** | **8** |
| **C: Energy production and conversion** |  |  |  |  |  |  |  |  |  |
| C | OEOE_0238 | OEOE_RS01135 | carbonic anhydrase | -1.37 | -1.39 | -1.80 | -2.07 | -2.15 | -2.08 |
| C | OEOE_0419 | OEOE_RS02015 | malate permease | 2.25 | 2.45 | 2.08 | 1.74 | 1.71 | 1.67 |
| C | OEOE_0423 | OEOE_RS02035 | citrate lyase subunit alpha | 0.73 | 1.13 | 0.93 | 0.59 | 0.54 | 0.64 |
| C | OEOE_0441 | OEOE_RS02125 | phosphosulfolactate synthase | 1.01 | 1.62 | 2.22 | 1.99 | 1.73 | 1.66 |
| C | OEOE_0516 | OEOE_RS02455 | NADH-dependent flavin oxidoreductase | -0.71 | -0.61 | -0.83 | -1.10 | -1.05 | -1.07 |
| C | OEOE_0550 | OEOE_RS02615 | ABC transporter permease | 1.57 | 1.86 | 2.17 | 2.27 | 2.08 | 2.00 |
| C | OEOE_0551 | OEOE_RS02620 | sulfonate ABC transporter ATP-binding protein | 1.70 | 1.70 | 1.71 | 1.82 | 1.78 | 1.62 |
| C | OEOE_0659 | OEOE_RS03155 | F0F1 ATP synthase subunit A | -1.03 | -1.08 | -0.42 | -0.31 | -0.35 | -0.37 |
| C | OEOE_0662 | OEOE_RS03170 | ATP synthase subunit delta | -0.92 | -1.16 | -0.92 | -0.90 | -0.91 | -0.91 |
| C | OEOE_0664 | OEOE_RS03180 | ATP synthase subunit gamma | -1.28 | -1.25 | -0.97 | -0.93 | -0.84 | -0.83 |
| C | OEOE_0666 | OEOE_RS03190 | F0F1 ATP synthase subunit epsilon | -1.35 | -1.26 | -1.45 | -1.49 | -1.56 | -1.69 |
| C | OEOE_0749 | OEOE_RS03585 | NADH-flavin reductase | -0.71 | -0.73 | -0.98 | -1.01 | -0.97 | -0.84 |
| C | OEOE_0829 | OEOE_RS03985 | energy-coupling factor transporter ATP-binding protein EcfA2 | -0.23 | -0.34 | -0.78 | -0.93 | -1.01 | -0.97 |
| C | OEOE_1046 | OEOE_RS05025 | NADH:flavin oxidoreductase | -1.09 | -1.54 | -1.76 | -1.70 | -1.70 | -1.51 |
| C | OEOE_1047 | OEOE_RS05030 | NADH:flavin oxidoreductase | -1.13 | -1.44 | -1.67 | -1.78 | -1.93 | -1.76 |
| C | OEOE_1087 | OEOE_RS05225 | energy-coupling factor transporter ATP-binding protein EcfA3 | -0.79 | -1.02 | -1.16 | -0.87 | -0.87 | -0.82 |
| C | OEOE_1110 | OEOE_RS05335 | Fe-S cluster formation protein, NifU | -0.37 | -0.25 | -0.76 | -0.95 | -1.04 | -1.01 |
| C | OEOE_1187 | OEOE_RS05720 | ABC transporter ATPase | -1.24 | -1.04 | -1.29 | -1.32 | -1.29 | -1.24 |
| C | OEOE_1290 | OEOE_RS06215 | NADPH:quinone reductase | -0.98 | -0.96 | -0.95 | -1.12 | -0.98 | -1.16 |
| C | OEOE_1446 | OEOE_RS06985 | malate transporter | 3.28 | 4.03 | 4.19 | 3.84 | 3.68 | 3.69 |
| C | OEOE_1838 | OEOE_RS08875 | pyridine nucleotide-disulfide oxidoreductase | -1.11 | -1.37 | -1.39 | -1.34 | -1.29 | -1.29 |
| **D: Cell cycle control, cell division, chromosome partitioning** |  |  |  |  |  |  |  |  |  |
| D | OEOE_0247 | OEOE_RS01180 | GTP-binding protein | -1.20 | -1.11 | -1.17 | -1.35 | -1.34 | -1.43 |
| D | OEOE_1150 | OEOE_RS05535 | cell division protein FtsL | -1.07 | -0.87 | -0.76 | -0.66 | -0.48 | -0.42 |
| D | OEOE_1261 | OEOE_RS06080 | cell division protein | 0.42 | 0.58 | 0.79 | 1.03 | 1.06 | 1.10 |
| **E: Amino acid transport and metabolism** |  |  |  |  |  |  |  |  |  |
| E | OEOE_0017 | OEOE_RS00080 | peptidase | -1.00 | -1.23 | -0.78 | -0.63 | -0.68 | -0.67 |
| E | OEOE_0149 | OEOE_RS00695 | chorismate mutase | 2.34 | 2.33 | 2.16 | 1.91 | 2.16 | 2.29 |
| E | OEOE_0239 | OEOE_RS01140 | O-acetylhomoserine aminocarboxypropyltransferase | -0.74 | -1.13 | -1.04 | -1.08 | -1.34 | -1.25 |
| E | OEOE_0267 | OEOE_RS01280 | peptide ABC transporter substrate-binding protein | 0.85 | 1.71 | 1.39 | 1.04 | 1.21 | 1.94 |
| E | OEOE_0268 | OEOE_RS01285 | branched-chain amino acid ABC transporter permease | 0.91 | 2.11 | 1.86 | 1.48 | 1.79 | 2.64 |
| E | OEOE_0269 | OEOE_RS01290 | phosphonate ABC transporter ATP-binding protein | 0.47 | 1.89 | 1.67 | 1.43 | 1.74 | 2.65 |
| E | OEOE_0270 | OEOE_RS01295 | peptide ABC transporter ATPase | 0.54 | 0.56 | 0.38 | 0.37 | 0.75 | 1.23 |
| E | OEOE_0287 | OEOE_RS01375 | D-alanine--poly(phosphoribitol) ligase | -2.24 | -2.07 | -2.01 | -1.87 | -1.93 | -1.95 |
| E | OEOE_0388 | OEOE_RS01865 | amino acid permease | 2.81 | 3.11 | 3.12 | 3.08 | 3.01 | 3.05 |
| E | OEOE_0438 | OEOE_RS02110 | peptide ABC transporter permease | 4.02 | 4.40 | 4.48 | 4.19 | 4.00 | 4.07 |
| E | OEOE_0443 | OEOE_RS02135 | branched-chain amino acid transporter II carrier protein | 1.11 | 1.39 | 1.64 | 1.64 | 1.78 | 1.95 |
| E | OEOE_0508 | OEOE_RS02415 | peptide methionine sulfoxide reductase | -1.26 | -1.25 | -0.81 | -0.56 | -0.55 | -0.54 |
| E | OEOE_0559 | OEOE_RS02660 | phosphate ABC transporter ATP-binding protein | 0.80 | 0.78 | 0.64 | 0.98 | 1.09 | 1.04 |
| E | OEOE_0570 | OEOE_RS02715 | ATP-dependent Clp protease proteolytic subunit | 0.14 | 0.41 | 0.47 | 0.05 | 0.10 | 0.02 |
| E | OEOE_0630 | OEOE_RS03010 | spermidine/putrescine import ATP-binding protein PotA | 0.69 | 0.61 | 0.99 | 1.27 | 1.21 | 1.20 |
| E | OEOE_0631 | OEOE_RS03015 | spermidine/putrescine ABC transporter permease | 0.53 | 0.65 | 0.84 | 1.14 | 1.08 | 1.03 |
| E | OEOE_0632 | OEOE_RS03020 | spermidine/purescine ABC transporter permease | 0.68 | 0.78 | 0.97 | 1.32 | 1.28 | 1.31 |
| E | OEOE_0751 | OEOE_RS03595 | amino acid ABC transporter substrate-binding protein | 1.06 | 1.25 | 0.58 | 0.41 | 0.58 | 0.58 |
| E | OEOE_0840 | OEOE_RS04040 | peptide ABC transporter substrate-binding protein | -0.57 | -0.91 | -1.16 | -1.22 | -1.15 | -1.15 |
| E | OEOE_0841 | OEOE_RS04045 | peptide ABC transporter substrate-binding protein | -1.51 | -1.62 | -1.98 | -2.12 | -2.10 | -2.04 |
| E | OEOE_0951 | OEOE_RS04560 | glutamine synthetase | 1.73 | 2.21 | 2.47 | 2.23 | 1.97 | 1.78 |
| E | OEOE_0959 | OEOE_RS04600 | Xaa-Pro aminopeptidase | 0.58 | 0.47 | 1.16 | 1.75 | 1.90 | 1.93 |
| E | OEOE_1055 | OEOE_RS05065 | ABC transporter permease | 1.25 | 0.86 | 0.62 | 0.62 | 0.58 | 0.56 |
| E | OEOE_1056 | OEOE_RS05070 | methionine import ATP-binding protein MetN 1 | 1.11 | 0.73 | 0.64 | 0.64 | 0.64 | 0.64 |
| E | OEOE_1092 | OEOE_RS05245 | oligoendopeptidase F | 0.56 | 0.66 | 0.83 | 1.17 | 1.59 | 1.90 |
| E | OEOE_1191 | OEOE_RS05740 | glutathione reductase | -0.63 | -0.78 | -0.83 | -0.96 | -1.00 | -0.98 |
| E | OEOE_1299 | OEOE_RS06260 | succinate-semialdehyde dehydrogenase | -1.07 | -1.14 | -1.40 | -1.39 | -1.24 | -1.20 |
| E | OEOE_1427 | OEOE_RS06890 | ABC transporter ATP-binding protein | -0.95 | -0.81 | -0.91 | -1.00 | -0.91 | -1.03 |
| E | OEOE_1464 | OEOE_RS07070 | spermidine/putrescine ABC transporter ATP-binding protein | 1.69 | 1.40 | 1.21 | 1.06 | 0.97 | 0.99 |
| E | OEOE_1465 | OEOE_RS07075 | spermidine/putrescine ABC transporter ATP-binding protein | 1.66 | 1.16 | 1.01 | 0.92 | 0.91 | 0.86 |
| E | OEOE_1466 | OEOE_RS07080 | spermidine/putrescine ABC transporter permease | 1.27 | 0.75 | 0.71 | 0.51 | 0.41 | 0.49 |
| E | OEOE_1538 | OEOE_RS07415 | peptide methionine sulfoxide reductase | -0.51 | -0.63 | -1.00 | -1.07 | -0.95 | -1.12 |
| E | OEOE_1562 | OEOE_RS07535 | S-ribosylhomocysteine lyase | -1.21 | -1.39 | -1.32 | -1.47 | -1.55 | -1.62 |
| E | OEOE_1615 | OEOE_RS07790 | peptide ABC transporter substrate-binding protein | -0.94 | -1.16 | -1.38 | -1.29 | -1.13 | -1.06 |
| E | OEOE_1616 | OEOE_RS07795 | peptide ABC transporter permease | -1.22 | -1.57 | -1.41 | -1.00 | -0.67 | -0.47 |
| E | OEOE_1638 | OEOE_RS07900 | amino acid permease | 2.93 | 3.16 | 3.18 | 2.98 | 2.90 | 2.81 |
| E | OEOE_1665 | OEOE_RS08035 | amino acid ABC transporter substrate-binding protein | 1.44 | 1.58 | 1.58 | 1.58 | 1.75 | 1.93 |
| E | OEOE_1667 | OEOE_RS08045 | amino acid ABC transporter permease | 1.28 | 1.24 | 1.19 | 1.28 | 1.53 | 1.51 |
| E | OEOE_1705 | OEOE_RS08230 | glyoxalase | -0.49 | -0.75 | -0.93 | -1.00 | -0.93 | -1.03 |
| E | OEOE_1717 | OEOE_RS08285 | ABC transporter substrate-binding protein | 0.83 | 0.85 | 0.93 | 1.34 | 1.56 | 1.56 |
| E | OEOE_1719 | OEOE_RS08295 | peptidase M20 | 0.89 | 1.14 | 1.08 | 1.35 | 1.49 | 1.53 |
| E | OEOE_1806 | OEOE_RS08705 | glutamine ABC transporter substrate-binding protein | 1.92 | 2.14 | 2.22 | 2.20 | 2.12 | 2.09 |
| **F. Nucleotide transport and metabolism** |  |  |  |  |  |  |  |  |  |
| F | OEOE_0259 | OEOE_RS01240 | dihydroorotase | -1.13 | -1.26 | -1.67 | -2.01 | -2.16 | -2.06 |
| F | OEOE_0262 | OEOE_RS01255 | orotidine 5'-phosphate decarboxylase | -0.62 | -0.82 | -1.07 | -1.44 | -1.63 | -1.64 |
| F | OEOE_0263 | OEOE_RS01260 | orotate phosphoribosyltransferase | -0.57 | -0.77 | -1.04 | -1.53 | -1.62 | -1.61 |
| F | OEOE_0264 | OEOE_RS01265 | dihydroorotate dehydrogenase | -0.63 | -0.70 | -1.05 | -1.42 | -1.54 | -1.51 |
| F | OEOE_0316 | OEOE_RS01510 | deoxyuridine 5'-triphosphate nucleotidohydrolase | -1.15 | -1.28 | -1.15 | -1.26 | -1.42 | -1.31 |
| F | OEOE_0320 | OEOE_RS01530 | nucleoside-triphosphate diphosphatase | 0.83 | 1.21 | 1.09 | 0.82 | 0.83 | 0.82 |
| F | OEOE_0376 | OEOE_RS01805 | uracil transporter | -0.90 | -1.09 | -0.64 | -0.21 | -0.30 | -0.45 |
| F | OEOE_0849 | OEOE_RS04085 | deoxyadenosine kinase | -1.23 | -1.25 | -1.08 | -1.07 | -1.13 | -1.24 |
| F | OEOE_0880 | OEOE_RS04215 | ribonucleoside-diphosphate reductase | -1.00 | -0.91 | -1.00 | -1.00 | -1.01 | -1.30 |
| F | OEOE_0969 | OEOE_RS04650 | nucleoside 2-deoxyribosyltransferase | 1.14 | 1.04 | 1.01 | 0.94 | 0.93 | 1.00 |
| F | OEOE_0981 | OEOE_RS04710 | nucleoside 2-deoxyribosyltransferase | 0.76 | 0.69 | 0.83 | 1.15 | 1.14 | 1.01 |
| F | OEOE_1033 | OEOE_RS04965 | uridine/cytidine kinase | -1.17 | -1.34 | -1.46 | -1.32 | -1.36 | -1.41 |
| F | OEOE_1069 | OEOE_RS05140 | adenine phosphoribosyltransferase | -1.32 | -1.32 | -1.41 | -1.42 | -1.51 | -1.43 |
| F | OEOE_1123 | OEOE_RS05400 | GMP synthase | -0.32 | -0.58 | -1.01 | -1.08 | -1.08 | -1.02 |
| F | OEOE_1413 | OEOE_RS06810 | deaminase | 1.11 | 1.55 | 1.80 | 2.09 | 1.76 | 1.56 |
| F | OEOE_1543 | OEOE_RS07440 | purine operon repressor | -1.10 | -1.30 | -1.42 | -1.55 | -1.62 | -1.50 |
| F | OEOE_1579 | OEOE_RS07615 | uracil phosphoribosyltransferase | -1.81 | -2.02 | -1.91 | -1.82 | -1.82 | -1.88 |
| **G: Carbohydrate transport and metabolism** |  |  |  |  |  |  |  |  |  |
| G | OEOE_0026 | OEOE_RS00125 | mannose-6-phosphate isomerase | -2.56 | -2.56 | -2.56 | -2.56 | -2.58 | -2.81 |
| G | OEOE_0077 | OEOE_RS00350 | diacetyl reductase [(S)-acetoin forming] | -1.31 | -1.59 | -1.80 | -1.93 | -1.90 | -1.81 |
| G | OEOE_0128 | OEOE_RS00595 | sugar phosphate isomerase | -1.71 | -1.98 | -2.05 | -1.72 | -1.58 | -1.71 |
| G | OEOE_0221 | OEOE_RS01045 | PTS sugar transporter subunit IIA | -0.63 | -0.61 | -0.79 | -0.90 | -0.96 | -1.01 |
| G | OEOE_0222 | OEOE_RS01050 | PTS cellobiose transporter subunit IIA | -0.39 | -0.70 | -0.86 | -0.97 | -1.06 | -0.86 |
| G | OEOE_0224 | OEOE_RS01060 | 6-phospho-β-glucosidase | -1.04 | -1.23 | -1.58 | -1.81 | -2.02 | -1.94 |
| G | OEOE_0233 | OEOE_RS01110 | PTS fructose transporter subunit IIA | -1.85 | -2.20 | -2.60 | -2.90 | -2.69 | -2.79 |
| G | OEOE_0234 | OEOE_RS01115 | PTS sugar transporter subunit IIA | -1.05 | -1.35 | -1.74 | -1.81 | -1.68 | -1.89 |
| G | OEOE_0235 | OEOE_RS01120 | PTS galactitol transporter subunit IIC | -0.44 | -0.23 | -0.70 | -1.05 | -1.13 | -1.02 |
| G | OEOE_0236 | OEOE_RS01125 | PTS galactitol transporter subunit IIB | -0.40 | -0.12 | -0.62 | -0.98 | -1.08 | -1.24 |
| G | OEOE_0253 | OEOE_RS01210 | sugar ABC transporter substrate-binding protein | 1.11 | 0.93 | 0.75 | 0.71 | 0.67 | 0.75 |
| G | OEOE_0254 | OEOE_RS01215 | sugar ABC transporter permease | 1.51 | 1.38 | 1.11 | 1.10 | 1.10 | 1.11 |
| G | OEOE_0255 | OEOE_RS01220 | sugar ABC transporter permease | 1.33 | 1.23 | 1.06 | 1.08 | 1.25 | 1.43 |
| G | OEOE_0303 | OEOE_RS01445 | peptidase S24 | -0.68 | -0.78 | -0.88 | -0.98 | -1.01 | -1.08 |
| G | OEOE_0324 | OEOE_RS01550 | aldehyde dehydrogenase | -0.48 | -0.72 | -1.13 | -1.35 | -1.39 | -1.43 |
| G | OEOE_0379 | OEOE_RS01820 | PTS mannose transporter subunit IID | 2.10 | 2.31 | 2.69 | 2.38 | 2.18 | 1.90 |
| G | OEOE_0380 | OEOE_RS01825 | PTS fructose transporter subunit IIC | 2.15 | 2.34 | 2.31 | 2.15 | 2.06 | 2.07 |
| G | OEOE_0381 | OEOE_RS01830 | PTS fructose transporter subunit IID | 1.84 | 2.20 | 2.22 | 1.97 | 1.79 | 1.70 |
| G | OEOE_0382 | OEOE_RS01835 | PTS sugar transporter subunit IIA | 1.38 | 1.90 | 1.90 | 1.61 | 1.49 | 1.44 |
| G | OEOE_0413 | OEOE_RS01985 | lactate dehydrogenase | -1.18 | -1.41 | -1.65 | -1.95 | -1.90 | -1.75 |
| G | OEOE_0464 | OEOE_RS02230 | PTS mannose transporter subunit IIAB | -0.73 | -1.02 | -1.31 | -1.38 | -1.06 | -1.00 |
| G | OEOE_0465 | OEOE_RS02235 | PTS alpha-glucoside transporter subunit IIBC | -0.48 | -0.83 | -0.97 | -1.12 | -0.85 | -0.81 |
| G | OEOE_0466 | OEOE_RS02240 | PTS mannose transporter subunit IID | -0.76 | -1.08 | -1.43 | -1.43 | -1.23 | -0.97 |
| G | OEOE_0643 | OEOE_RS03075 | phosphocarrier protein HPr | -2.19 | -2.16 | -2.20 | -2.33 | -2.25 | -2.32 |
| G | OEOE_0644 | OEOE_RS03080 | glucosamine-6-phosphate deaminase | -1.27 | -1.16 | -1.51 | -1.86 | -1.95 | -1.79 |
| G | OEOE_1182 | OEOE_RS05695 | lactate dehydrogenase | -1.20 | -0.93 | -1.10 | -1.22 | -1.14 | -1.37 |
| G | OEOE_1204 | OEOE_RS05805 | PTS sugar transporter | -2.37 | -2.45 | -2.68 | -2.86 | -2.87 | -2.90 |
| G | OEOE_1341 | OEOE_RS06450 | PTS β-glucoside transporter subunit IIABC | -0.94 | -1.26 | -1.40 | -1.23 | -0.89 | -0.56 |
| G | OEOE_1342 | OEOE_RS06455 | PTS sugar transporter subunit IIA | -1.31 | -1.61 | -1.41 | -0.92 | -0.50 | -0.39 |
| G | OEOE_1456 | OEOE_RS07030 | glycerol-3-phosphate ABC transporter ATP-binding protein | -1.42 | -1.92 | -2.10 | -2.34 | -2.51 | -2.54 |
| G | OEOE_1457 | OEOE_RS07035 | glycerol-3-phosphate ABC transporter permease | -0.90 | -1.36 | -1.53 | -1.65 | -1.72 | -1.76 |
| G | OEOE_1458 | OEOE_RS07040 | glycerol-3-phosphate ABC transporter permease | -1.03 | -1.54 | -1.64 | -1.81 | -2.01 | -1.93 |
| G | OEOE_1459 | OEOE_RS07045 | glycerol-3-phosphate ABC transporter substrate-binding protein | -1.16 | -1.47 | -1.86 | -2.13 | -2.20 | -2.22 |
| G | OEOE_1504 | OEOE_RS07255 | UDP-phosphate galactose phosphotransferase | -1.98 | -1.96 | -1.96 | -2.01 | -1.94 | -1.96 |
| G | OEOE_1532 | OEOE_RS07385 | fructosamine kinase | 0.31 | 0.11 | 0.48 | 0.87 | 0.83 | 1.05 |
| G | OEOE_1602 | OEOE_RS07730 | diacetyl reductase | -0.63 | -1.01 | -1.25 | -1.35 | -1.37 | -1.43 |
| G | OEOE_1609 | OEOE_RS07760 | sugar ABC transporter permease | -0.38 | -0.52 | -1.03 | -1.23 | -1.22 | -1.21 |
| G | OEOE_1612 | OEOE_RS07775 | ribokinase | -1.53 | -1.87 | -2.32 | -2.18 | -1.64 | -1.57 |
| G | OEOE_1613 | OEOE_RS07780 | D-ribose pyranase | -1.27 | -1.82 | -2.09 | -2.08 | -1.59 | -1.33 |
| G | OEOE_1614 | OEOE_RS07785 | sugar:proton symporter | -1.28 | -1.75 | -2.22 | -2.19 | -1.73 | -1.56 |
| G | OEOE_1651 | OEOE_RS07965 | sugar ABC transporter ATP-binding protein | -0.89 | -0.80 | -0.88 | -1.06 | -1.06 | -1.04 |
| G | OEOE_1669 | OEOE_RS08055 | sugar phosphate isomerase | -1.35 | -1.33 | -1.79 | -2.11 | -2.02 | -2.24 |
| G | OEOE_1708 | OEOE_RS08245 | fructokinase | -1.42 | -2.08 | -2.15 | -2.28 | -2.40 | -2.46 |
| G | OEOE_1714 | OEOE_RS08275 | glucose transporter | 0.62 | 0.81 | 0.82 | 1.17 | 1.31 | 1.30 |
| **H: Coenzyme Metabolism** |  |  |  |  |  |  |  |  |  |
| H | OEOE_0327 | OEOE_RS01565 | lipoate-protein ligase A | -1.07 | -1.21 | -1.27 | -1.53 | -1.43 | -1.24 |
| H | OEOE_0676 | OEOE_RS03240 | dephospho-CoA kinase | -0.88 | -1.23 | -1.38 | -1.43 | -1.26 | -1.23 |
| H | OEOE_0779 | OEOE_RS03730 | 5-formyltetrahydrofolate cyclo-ligase | 0.66 | 1.13 | 1.35 | 1.08 | 0.88 | 0.90 |
| H | OEOE_0791 | OEOE_RS03790 | thiamine pyrophosphokinase | -0.93 | -1.07 | -1.05 | -0.96 | -0.93 | -0.84 |
| H | OEOE_1036 | OEOE_RS04980 | pyridoxal biosynthesis protein | -0.89 | -1.10 | -1.22 | -1.19 | -1.23 | -1.15 |
| H | OEOE_1473 | OEOE_RS07110 | 6-pyruvoyl-tetrahydropterin synthase | 1.22 | 1.00 | 0.59 | 0.57 | 0.60 | 0.59 |
| H | OEOE_1597 | OEOE_RS07705 | biotin transporter | 0.41 | 0.93 | 0.94 | 1.04 | 0.94 | 0.85 |
| H | OEOE_1642 | OEOE_RS07920 | holo-ACP synthase | 2.36 | 2.55 | 2.69 | 2.94 | 2.85 | 2.58 |
| H | OEOE_1834 | OEOE_RS08855 | 1,4-dihydroxy-2-naphthoate octaprenyltransferase | 1.03 | 1.04 | 0.97 | 0.99 | 0.97 | 0.85 |
| **I: Lipid transport and metabolism** |  |  |  |  |  |  |  |  |  |
| I | OEOE_0145 | OEOE_RS00675 | phospholipid phosphatase | -1.52 | -1.50 | -1.25 | -1.04 | -1.13 | -1.27 |
| I | OEOE_0485 | OEOE_RS02315 | phospholipid phosphatase | -1.35 | -1.88 | -1.76 | -1.75 | -1.75 | -1.79 |
| I | OEOE_1015 | OEOE_RS04875 | lysophospholipase | 0.78 | 0.87 | 1.10 | 1.14 | 1.06 | 1.16 |
| I | OEOE_1049 | OEOE_RS05040 | tannase | 1.88 | 2.26 | 2.72 | 2.22 | 1.89 | 1.90 |
| I | OEOE_1176 | OEOE_RS05660 | cyclopropane-fatty-acyl-phospholipid synthase | -0.90 | -1.11 | -0.98 | -0.90 | -0.86 | -0.83 |
| I | OEOE_1292 | OEOE_RS06225 | esterase | -1.02 | -0.96 | -0.96 | -1.12 | -0.99 | -0.82 |
| I | OEOE_1343 | OEOE_RS06460 | lysophospholipase | -0.32 | -0.43 | -0.93 | -1.22 | -1.18 | -1.14 |
| I | OEOE_1366 | OEOE_RS06570 | acyl-CoA synthetase | -1.33 | -1.62 | -2.00 | -1.78 | -1.70 | -1.61 |
| I | OEOE_1460 | OEOE_RS07050 | glycerophosphoryl diester phosphodiesterase | -1.49 | -1.64 | -2.08 | -2.51 | -2.57 | -2.56 |
| I | OEOE_1675 | OEOE_RS08085 | lipid kinase | 1.42 | 1.44 | 1.51 | 1.38 | 1.29 | 1.37 |
| **J: Translation, ribosomal structure and biogenesis** |  |  |  |  |  |  |  |  |  |
| J | OEOE_0014 | OEOE_RS00065 | 50S ribosomal protein L9 | 0.01 | 0.53 | 1.04 | 1.12 | 0.79 | 0.52 |
| J | OEOE_0019 | OEOE_RS00090 | D-aminoacyl-tRNA deacylase | -1.33 | -1.53 | -1.57 | -1.47 | -1.41 | -1.26 |
| J | OEOE_0176 | OEOE_RS00835 | pseudouridylate synthase | 1.06 | 1.19 | 1.00 | 0.89 | 0.94 | 0.88 |
| J | OEOE_0321 | OEOE_RS01535 | glutamate--tRNA ligase | 0.82 | 1.33 | 1.26 | 1.06 | 1.06 | 1.10 |
| J | OEOE_0429 | OEOE_RS02065 | ribosome maturation factor RimP | 0.93 | 1.00 | 1.41 | 1.79 | 1.83 | 1.74 |
| J | OEOE_0432 | OEOE_RS02080 | 50S ribosomal protein L7ae | 1.06 | 0.95 | 1.06 | 1.18 | 1.23 | 1.28 |
| J | OEOE_0433 | OEOE_RS02085 | translation initiation factor IF-2 | 0.68 | 0.63 | 0.81 | 1.02 | 1.08 | 1.01 |
| J | OEOE_0434 | OEOE_RS02090 | ribosome-binding factor A | 0.48 | 0.54 | 0.83 | 1.07 | 1.14 | 1.02 |
| J | OEOE_0440 | OEOE_RS02120 | serine--tRNA ligase | 1.44 | 1.93 | 2.07 | 1.93 | 1.77 | 1.72 |
| J | OEOE_0517 | OEOE_RS02460 | elongation factor 3 | 1.48 | 1.97 | 2.28 | 1.95 | 1.83 | 1.82 |
| J | OEOE_0581 | OEOE_RS02770 | elongation factor 4 | 0.80 | 1.09 | 0.89 | 0.74 | 0.60 | 0.69 |
| J | OEOE_0589 | OEOE_RS02810 | histidyl-tRNA synthetase | 0.69 | 0.65 | 0.84 | 0.94 | 0.93 | 1.08 |
| J | OEOE_0594 | OEOE_RS02840 | 30S ribosomal protein S10 | 1.88 | 1.82 | 1.88 | 1.92 | 1.93 | 1.90 |
| J | OEOE_0595 | OEOE_RS02845 | 50S ribosomal protein L3 | 1.73 | 1.63 | 1.68 | 1.70 | 1.66 | 1.66 |
| J | OEOE_0596 | OEOE_RS02850 | 50S ribosomal protein L4 | 1.20 | 1.18 | 1.19 | 1.16 | 1.18 | 1.16 |
| J | OEOE_0598 | OEOE_RS02860 | 50S ribosomal protein L2 | 1.38 | 1.39 | 1.29 | 1.29 | 1.29 | 1.29 |
| J | OEOE_0599 | OEOE_RS02865 | 30S ribosomal protein S19 | 1.50 | 1.47 | 1.61 | 1.52 | 1.53 | 1.48 |
| J | OEOE_0600 | OEOE_RS02870 | 50S ribosomal protein L22 | 1.44 | 1.40 | 1.40 | 1.41 | 1.40 | 1.37 |
| J | OEOE_0601 | OEOE_RS02875 | 30S ribosomal protein S3 | 1.66 | 1.66 | 1.74 | 1.73 | 1.66 | 1.66 |
| J | OEOE_0602 | OEOE_RS02880 | 50S ribosomal protein L16 | 1.44 | 1.41 | 1.41 | 1.46 | 1.49 | 1.36 |
| J | OEOE_0603 | OEOE_RS02885 | 50S ribosomal protein L29 | 1.66 | 1.69 | 1.69 | 1.75 | 1.79 | 1.70 |
| J | OEOE_0604 | OEOE_RS02890 | 30S ribosomal protein S17 | 1.52 | 1.43 | 1.53 | 1.61 | 1.56 | 1.64 |
| J | OEOE_0605 | OEOE_RS02895 | 50S ribosomal protein L14 | 1.00 | 1.00 | 1.03 | 1.05 | 1.13 | 1.06 |
| J | OEOE_0606 | OEOE_RS02900 | 50S ribosomal protein L24 | 1.40 | 1.39 | 1.44 | 1.51 | 1.55 | 1.42 |
| J | OEOE_0607 | OEOE_RS02905 | 50S ribosomal protein L5 | 1.50 | 1.50 | 1.66 | 1.79 | 1.74 | 1.62 |
| J | OEOE_0608 | OEOE_RS02910 | 30S ribosomal protein S8 | 2.07 | 2.04 | 2.35 | 2.57 | 2.45 | 2.42 |
| J | OEOE_0609 | OEOE_RS02915 | 50S ribosomal protein L6 | 1.27 | 1.29 | 1.41 | 1.43 | 1.46 | 1.32 |
| J | OEOE_0610 | OEOE_RS02920 | 50S ribosomal protein L18 | 1.53 | 1.56 | 1.76 | 1.91 | 1.90 | 1.77 |
| J | OEOE_0611 | OEOE_RS02925 | 30S ribosomal protein S5 | 0.98 | 0.97 | 1.05 | 1.11 | 1.04 | 1.05 |
| J | OEOE_0612 | OEOE_RS02930 | 50S ribosomal protein L30 | 0.99 | 0.98 | 1.04 | 1.17 | 1.19 | 1.20 |
| J | OEOE_0613 | OEOE_RS02935 | 50S ribosomal protein L15 | 1.00 | 1.18 | 1.41 | 1.70 | 1.64 | 1.63 |
| J | OEOE_0619 | OEOE_RS02960 | 30S ribosomal protein S11 | 0.64 | 0.82 | 0.95 | 1.09 | 1.13 | 1.10 |
| J | OEOE_0621 | OEOE_RS02970 | 50S ribosomal protein L17 | 0.56 | 0.93 | 1.41 | 1.75 | 1.83 | 1.75 |
| J | OEOE_0629 | OEOE_RS03005 | 30S ribosomal protein S9 | 0.48 | 0.45 | 0.91 | 1.28 | 1.24 | 1.02 |
| J | OEOE_0739 | OEOE_RS03530 | 2'-5' RNA ligase | -1.26 | -1.35 | -1.35 | -1.47 | -1.39 | -1.69 |
| J | OEOE_0770 | OEOE_RS03680 | 50S ribosomal protein L32 | -0.14 | 0.59 | 0.78 | 0.87 | 0.88 | 1.07 |
| J | OEOE_0798 | OEOE_RS03825 | 30S ribosomal protein S16 | -1.55 | -1.20 | -1.26 | -1.24 | -1.26 | -1.37 |
| J | OEOE_0800 | OEOE_RS03835 | ribosome maturation factor RimM | -0.93 | -1.10 | -0.86 | -0.94 | -0.94 | -0.93 |
| J | OEOE_0834 | OEOE_RS04010 | acetyltransferase | 2.37 | 2.70 | 2.99 | 3.18 | 3.07 | 2.77 |
| J | OEOE_0899 | OEOE_RS04310 | arginine--tRNA ligase | 1.78 | 2.04 | 1.78 | 1.78 | 1.76 | 1.79 |
| J | OEOE_0917 | OEOE_RS04400 | phenylalanine--tRNA ligase subunit alpha | -1.23 | -1.31 | -1.03 | -0.78 | -0.70 | -0.45 |
| J | OEOE_0957 | OEOE_RS04590 | 50S ribosomal protein L21 | 0.52 | 0.76 | 1.06 | 1.20 | 1.20 | 1.18 |
| J | OEOE_0994 | OEOE_RS04775 | glycine--tRNA ligase subunit β | -1.04 | -0.79 | -0.40 | -0.16 | 0.08 | 0.05 |
| J | OEOE_1079 | OEOE_RS05185 | RNA pseudouridine synthase | 0.73 | 0.82 | 0.87 | 0.87 | 0.90 | 1.02 |
| J | OEOE_1151 | OEOE_RS05540 | ribosomal RNA small subunit methyltransferase H | -1.32 | -1.30 | -0.82 | -0.61 | -0.52 | -0.24 |
| J | OEOE_1158 | OEOE_RS05575 | pseudouridine synthase | 0.70 | 1.02 | 0.98 | 0.87 | 0.88 | 0.95 |
| J | OEOE_1228 | OEOE_RS05915 | RNA-binding protein | -1.73 | -1.31 | -1.73 | -1.81 | -1.93 | -1.98 |
| J | OEOE_1267 | OEOE_RS06100 | peptide chain release factor 3 | 1.07 | 0.84 | 0.75 | 0.75 | 0.73 | 0.71 |
| J | OEOE_1278 | OEOE_RS06155 | 50S ribosomal protein L20 | 0.73 | 0.82 | 0.92 | 1.06 | 1.18 | 1.11 |
| J | OEOE_1279 | OEOE_RS06160 | 50S ribosomal protein L35 | 0.92 | 0.97 | 1.37 | 1.65 | 1.73 | 1.62 |
| J | OEOE_1283 | OEOE_RS06180 | 30S ribosomal protein S15 | 0.32 | 1.02 | 1.14 | 1.33 | 1.28 | 1.00 |
| J | OEOE_1284 | OEOE_RS06185 | 30S ribosomal protein S20 | 0.29 | 0.84 | 1.07 | 1.29 | 1.32 | 0.83 |
| J | OEOE_1289 | OEOE_RS06210 | queuine tRNA-ribosyltransferase | 1.50 | 1.74 | 1.92 | 1.86 | 1.84 | 1.83 |
| J | OEOE_1360 | OEOE_RS06545 | raiA ribosome-associated inhibitor A | -1.35 | -1.40 | -1.06 | -1.11 | -1.27 | -1.47 |
| J | OEOE_1414 | OEOE_RS06815 | 16S RNA G1207 methylase RsmC | 0.61 | 1.05 | 1.24 | 1.40 | 1.16 | 1.05 |
| J | OEOE_1416 | OEOE_RS06825 | 50S ribosomal protein L7/L12 | 0.63 | 1.16 | 1.71 | 2.00 | 1.74 | 1.46 |
| J | OEOE_1417 | OEOE_RS06830 | 50S ribosomal protein L10 | 0.87 | 1.20 | 1.27 | 1.56 | 1.48 | 1.30 |
| J | OEOE_1418 | OEOE_RS06835 | 50S ribosomal protein L1 | 0.58 | 1.23 | 1.31 | 1.52 | 1.37 | 1.23 |
| J | OEOE_1419 | OEOE_RS06840 | 50S ribosomal protein L11 | 0.59 | 0.98 | 1.05 | 1.14 | 1.14 | 0.94 |
| J | OEOE_1549 | OEOE_RS07470 | ribosomal RNA small subunit methyltransferase A | 0.11 | 0.57 | 0.97 | 1.00 | 1.07 | 1.14 |
| J | OEOE_1559 | OEOE_RS07520 | cysteine--tRNA ligase | -0.32 | -0.54 | -0.81 | -0.98 | -1.03 | -1.04 |
| J | OEOE_1674 | OEOE_RS08080 | RNA methyltransferase | 0.99 | 1.09 | 1.02 | 0.95 | 0.88 | 0.90 |
| J | OEOE_1813 | OEOE_RS08745 | prolyl-tRNA synthetase | -1.03 | -1.03 | -1.08 | -1.03 | -1.07 | -1.11 |
| J | OEOE_1863 | OEOE_RS08995 | ribonuclease P protein component | 0.38 | 0.90 | 1.14 | 0.85 | 0.82 | 0.64 |
| **K: Transcription** |  |  |  |  |  |  |  |  |  |
| K | OEOE_0047 | OEOE_RS00215 | XRE family transcriptional regulator | 1.15 | 1.12 | 1.21 | 1.04 | 0.95 | 0.95 |
| K | OEOE_0099 | OEOE_RS00455 | XRE family transcriptional regulator | -1.17 | -1.24 | -1.42 | -1.38 | -1.39 | -1.55 |
| K | OEOE_0179 | OEOE_RS00850 | TetR family transcriptional regulator | 0.80 | 1.16 | 1.30 | 0.90 | 0.69 | 0.70 |
| K | OEOE_0195 | OEOE_RS00930 | XRE family transcriptional regulator | -2.45 | -2.43 | -2.04 | -2.05 | -2.24 | -2.05 |
| K | OEOE_0209 | OEOE_RS00995 | AraC family transcriptional regulator | -1.87 | -2.17 | -1.91 | -1.87 | -1.86 | -1.85 |
| K | OEOE_0215 | OEOE_RS01020 | TetR family transcriptional regulator | -1.50 | -1.49 | -1.45 | -1.49 | -1.57 | -1.63 |
| K | OEOE_0225 | OEOE_RS01065 | transcriptional regulator | -2.34 | -2.52 | -2.45 | -2.34 | -2.47 | -2.28 |
| K | OEOE_0232 | OEOE_RS01105 | transcriptional antiterminator | -2.81 | -3.13 | -2.90 | -2.68 | -2.43 | -2.84 |
| K | OEOE_0245 | OEOE_RS01170 | TetR family transcriptional regulator | -2.55 | -2.84 | -2.68 | -2.59 | -2.50 | -2.55 |
| K | OEOE_0302 | OEOE_RS01440 | LacI family transcriptional regulator | -0.82 | -0.72 | -0.86 | -0.89 | -1.03 | -0.97 |
| K | OEOE_0417 | OEOE_RS02005 | citrate lyase | 0.82 | 1.01 | 0.76 | 0.63 | 0.55 | 0.49 |
| K | OEOE_0430 | OEOE_RS02070 | transcription termination factor NusA | 1.27 | 1.20 | 1.41 | 1.59 | 1.59 | 1.69 |
| K | OEOE_0435 | OEOE_RS02095 | transcriptional regulator | 1.13 | 1.40 | 1.36 | 1.35 | 1.36 | 1.41 |
| K | OEOE_0540 | OEOE_RS02565 | MarR family transcriptional regulator | -0.28 | -0.45 | -1.01 | -1.02 | -0.84 | -1.19 |
| K | OEOE_0569 | OEOE_RS02710 | sporulation protein | 0.89 | 1.23 | 1.15 | 0.97 | 0.97 | 0.97 |
| K | OEOE_0704 | OEOE_RS03375 | ArsR family transcriptional regulator | 0.97 | 1.32 | 1.91 | 1.76 | 1.47 | 1.35 |
| K | OEOE_0718 | OEOE_RS03430 | transcriptional regulator | 2.09 | 2.68 | 2.49 | 2.01 | 1.78 | 1.61 |
| K | OEOE_0724 | OEOE_RS03455 | transcriptional regulator | 0.66 | 0.70 | 0.94 | 1.08 | 0.89 | 0.89 |
| K | OEOE_0728 | OEOE_RS03475 | MarR family transcriptional regulator | 1.07 | 0.37 | 0.58 | 0.57 | 0.60 | 0.61 |
| K | OEOE_0733 | OEOE_RS03500 | TetR family transcriptional regulator | -1.12 | -0.68 | -0.17 | -0.41 | -0.47 | -0.65 |
| K | OEOE_0793 | OEOE_RS03800 | XRE family transcriptional regulator | 1.87 | 2.01 | 2.13 | 2.04 | 2.09 | 2.13 |
| K | OEOE_0808 | OEOE_RS03880 | LacI family transcriptional regulator | -0.76 | -1.12 | -0.96 | -0.93 | -0.93 | -1.08 |
| K | OEOE_0926 | OEOE_RS04440 | ArsR family transcriptional regulator | 0.64 | 0.66 | 1.34 | 1.62 | 1.42 | 1.55 |
| K | OEOE_0996 | OEOE_RS04785 | RNA polymerase sigma factor RpoD | 0.58 | 0.83 | 1.03 | 1.23 | 1.18 | 0.98 |
| K | OEOE_1010 | OEOE_RS04850 | transcriptional regulator | -0.96 | -1.03 | -1.12 | -1.17 | -1.17 | -1.27 |
| K | OEOE_1045 | OEOE_RS05020 | LysR family transcriptional regulator | -1.19 | -1.51 | -1.85 | -1.75 | -1.52 | -1.50 |
| K | OEOE_1152 | OEOE_RS05545 | transcriptional regulator MraZ | -1.71 | -1.57 | -1.27 | -1.00 | -0.88 | -0.66 |
| K | OEOE_1238 | OEOE_RS05965 | transcription termination factor NusB | -1.18 | -1.18 | -0.56 | -0.43 | -0.53 | -0.11 |
| K | OEOE_1297 | OEOE_RS06250 | MarR family transcriptional regulator | -0.48 | -0.38 | -0.31 | -0.70 | -0.52 | -1.04 |
| K | OEOE_1346 | OEOE_RS06470 | ArsR family transcriptional regulator | -1.04 | -1.52 | -1.52 | -1.82 | -1.56 | -1.89 |
| K | OEOE_1376 | OEOE_RS06620 | cold-shock protein | -1.75 | -1.14 | -1.30 | -1.49 | -1.73 | -1.81 |
| K | OEOE_1450 | OEOE_RS07005 | cell filamentation protein Fic | -0.52 | -0.86 | -1.03 | -1.07 | -0.88 | -0.94 |
| K | OEOE_1455 | OEOE_RS07025 | AraC family transcriptional regulator | -1.48 | -1.52 | -1.59 | -1.62 | -1.71 | -1.60 |
| K | OEOE_1467 | OEOE_RS07085 | GntR family transcriptional regulator | -1.01 | -1.11 | -1.16 | -1.21 | -0.92 | -0.89 |
| K | OEOE_1572 | OEOE_RS07585 | AraC family transcriptional regulator | -1.53 | -1.68 | -1.79 | -1.69 | -1.60 | -1.60 |
| K | OEOE_1575 | OEOE_RS07600 | AraC family transcriptional regulator | -1.12 | -1.31 | -1.50 | -1.40 | -1.30 | -1.28 |
| K | OEOE_1685 | OEOE_RS08130 | TetR family transcriptional regulator | 0.77 | 0.96 | 1.18 | 1.32 | 1.25 | 1.21 |
| K | OEOE_1733 | OEOE_RS08360 | RNA polymerase I and III, subunit | 0.35 | 0.22 | 0.30 | 0.65 | 0.98 | 1.00 |
| K | OEOE_1765 | OEOE_RS08510 | MarR family transcriptional regulator | -2.63 | -2.44 | -2.26 | -2.39 | -2.46 | -2.36 |
| **L: Replication, recombination and repair** |  |  |  |  |  |  |  |  |  |
| L | OEOE_0308 | OEOE_RS01470 | ATP-dependent helicase/deoxyribonuclease subunit B | 0.35 | 0.95 | 1.02 | 0.52 | 0.37 | 0.35 |
| L | OEOE_0309 | OEOE_RS01475 | ATP-dependent helicase/nuclease subunit A | 0.43 | 0.75 | 1.23 | 1.07 | 0.87 | 0.77 |
| L | OEOE_0318 | OEOE_RS01520 | DNA repair protein RadA | 1.02 | 1.38 | 1.19 | 1.01 | 0.87 | 0.93 |
| L | OEOE_0795 | OEOE_RS03810 | DNA polymerase III subunit alpha | 0.44 | 0.73 | 0.89 | 1.03 | 1.09 | 1.25 |
| L | OEOE_0799 | OEOE_RS03830 | methylated DNA-protein cysteine methyltransferase | -1.29 | -1.54 | -0.92 | -1.16 | -1.23 | -1.11 |
| L | OEOE_0990 | OEOE_RS04755 | DNA repair protein RecO | -1.40 | -1.40 | -1.31 | -1.18 | -1.31 | -1.44 |
| L | OEOE_0995 | OEOE_RS04780 | DNA primase | 0.82 | 1.17 | 1.31 | 1.63 | 1.53 | 1.47 |
| L | OEOE_1020 | OEOE_RS04900 | DNA-binding protein | 0.83 | 1.01 | 1.00 | 0.96 | 0.99 | 1.05 |
| L | OEOE_1021 | OEOE_RS04905 | DNA topoisomerase I | 0.97 | 0.88 | 0.77 | 1.35 | 1.58 | 1.64 |
| L | OEOE_1023 | OEOE_RS04915 | tyrosine recombinase XerC | -1.19 | -1.26 | -1.03 | -0.68 | -0.51 | -0.40 |
| L | OEOE_1094 | OEOE_RS05255 | Holliday junction DNA helicase RecU | -1.09 | -1.22 | -0.91 | -0.77 | -0.55 | -0.68 |
| L | OEOE_1096 | OEOE_RS05265 | DNA replication protein DnaD | -1.10 | -1.20 | -1.20 | -1.12 | -1.11 | -1.12 |
| L | OEOE_1099 | OEOE_RS05280 | DNA helicase | -0.92 | -1.12 | -0.97 | -0.81 | -0.88 | -0.78 |
| L | OEOE_1106 | OEOE_RS05315 | ATPase AAA | 0.84 | 0.56 | 0.90 | 1.03 | 0.99 | 0.98 |
| L | OEOE_1382 | OEOE_RS06650 | protein RecA | 0.92 | 0.84 | 0.95 | 1.18 | 1.21 | 1.28 |
| L | OEOE_1440 | OEOE_RS06955 | ATP-dependent DNA helicase | 1.22 | 1.52 | 1.52 | 1.63 | 1.47 | 1.31 |
| L | OEOE_1525 | OEOE_RS07355 | DNA replication initiation protein | 0.65 | 0.81 | 1.18 | 1.05 | 0.81 | 0.88 |
| **M: Cell wall/membrane/envelope biogenesis** |  |  |  |  |  |  |  |  |  |
| M | OEOE_0121 | OEOE_RS00560 | D-alanyl-D-alanine carboxypeptidase | -1.41 | -1.41 | -1.53 | -1.41 | -1.41 | -1.45 |
| M | OEOE_0197 | OEOE_RS00940 | hemolysin D | 2.23 | 1.79 | 1.60 | 1.55 | 1.53 | 1.47 |
| M | OEOE_0218 | OEOE_RS01030 | glycosyltransferase | -1.17 | -1.38 | -1.31 | -1.39 | -1.49 | -1.51 |
| M | OEOE_0284 | OEOE_RS01360 | D-Ala-teichoic acid biosynthesis protein | -1.03 | -1.36 | -1.30 | -1.30 | -1.40 | -1.59 |
| M | OEOE_0286 | OEOE_RS01370 | D-alanyl-lipoteichoic acid biosynthesis protein DltB | -1.93 | -1.81 | -1.81 | -1.81 | -1.92 | -1.82 |
| M | OEOE_0288 | OEOE_RS01380 | D-alanyl-lipoteichoic acid biosynthesis protein DltD | -1.60 | -1.55 | -1.61 | -1.66 | -1.66 | -1.71 |
| M | OEOE_0696 | OEOE_RS03340 | aggregation promoting factor surface protein | -2.57 | -2.92 | -2.81 | -2.61 | -2.48 | -2.29 |
| M | OEOE_0719 | OEOE_RS03435 | D-alanyl-D-alanine carboxypeptidase | 5.53 | 5.83 | 6.01 | 5.82 | 5.61 | 5.64 |
| M | OEOE_0850 | OEOE_RS04090 | large-conductance mechanosensitive channel | -1.73 | -1.73 | -1.79 | -1.78 | -1.69 | -1.99 |
| M | OEOE_0882 | OEOE_RS04225 | peptidoglycan interpeptide bridge formation protein | -1.09 | -1.22 | -1.09 | -1.09 | -1.12 | -1.09 |
| M | OEOE_0988 | OEOE_RS04745 | diacylglycerol kinase | -0.87 | -1.05 | -0.08 | 0.46 | 0.22 | 0.22 |
| M | OEOE_1199 | OEOE_RS05780 | peptidoglycan-binding protein | -1.16 | -1.56 | -1.79 | -1.80 | -1.59 | -1.43 |
| M | OEOE_1332 | OEOE_RS06410 | 2-dehydro-3-deoxyphosphooctonate aldolase | 0.39 | 0.82 | 1.05 | 0.53 | 0.30 | 0.23 |
| M | OEOE_1430 | OEOE_RS06905 | peptidoglycan-binding protein | -0.67 | -1.02 | -1.31 | -1.42 | -1.27 | -1.31 |
| M | OEOE_1442 | OEOE_RS06965 | peptidoglycan interpeptide bridge formation protein | 0.95 | 1.87 | 2.52 | 2.39 | 1.85 | 1.70 |
| M | OEOE_1443 | OEOE_RS06970 | sortase | 1.46 | 2.00 | 2.48 | 2.27 | 1.94 | 1.91 |
| M | OEOE_1444 | OEOE_RS06975 | peptidoglycan interpeptide bridge formation protein | 1.55 | 2.27 | 2.77 | 2.52 | 2.24 | 2.19 |
| M | OEOE_1451 | OEOE_RS07010 | glycosyl transferase | -2.14 | -2.39 | -2.08 | -2.03 | -2.02 | -1.73 |
| M | OEOE_1452 | OEOE_RS07015 | glycosyl transferase | -0.98 | -1.08 | -1.07 | -0.84 | -0.69 | -0.47 |
| M | OEOE_1502 | OEOE_RS07245 | glycosyl transferase family 1 | -1.15 | -1.24 | -1.25 | -1.26 | -1.38 | -1.36 |
| M | OEOE_1503 | OEOE_RS07250 | glycosyl transferase | -1.78 | -1.78 | -1.78 | -1.83 | -1.80 | -1.83 |
| M | OEOE_1506 | OEOE_RS07265 | capsular polysaccharide biosynthesis protein | -2.39 | -2.18 | -1.99 | -1.88 | -1.89 | -1.71 |
| M | OEOE_1561 | OEOE_RS07530 | D-alanyl-D-alanine carboxypeptidase | -0.96 | -1.45 | -1.49 | -1.60 | -1.75 | -1.70 |
| M | OEOE_1568 | OEOE_RS07565 | capsular polysaccharide biosynthesis protein | -1.35 | -1.37 | -1.42 | -1.35 | -1.38 | -1.35 |
| M | OEOE_1621 | OEOE_RS07820 | glycosyl transferase | 1.78 | 1.90 | 2.86 | 3.39 | 3.35 | 3.08 |
| M | OEOE_1666 | OEOE_RS08040 | polar amino acid ABC transporter ATPase | 1.04 | 1.07 | 1.02 | 1.07 | 1.31 | 1.45 |
| M | OEOE_1689 | OEOE_RS08150 | macrolide ABC transporter ATP-binding protein | 1.11 | 1.45 | 1.84 | 1.80 | 1.47 | 1.35 |
| M | OEOE_1839 | OEOE_RS08880 | peptidoglycan-binding protein | -1.45 | -1.48 | -1.90 | -1.66 | -1.11 | -0.85 |
| **O: Post-translational modification, protein turnover, and chaperones** |  |  |  |  |  |  |  |  |  |
| O | OEOE_0139 | OEOE_RS00645 | glutaredoxin | -1.04 | -0.44 | -0.27 | -0.64 | -0.82 | -0.86 |
| O | OEOE_0189 | OEOE_RS00900 | disulfide bond formation protein | 0.34 | 0.64 | 0.80 | 0.94 | 0.98 | 1.00 |
| O | OEOE_0289 | OEOE_RS01385 | heat-shock protein Hsp20 | -0.92 | -0.43 | -0.55 | -0.89 | -0.88 | -1.13 |
| O | OEOE_1115 | OEOE_RS05360 | anaerobic ribonucleoside-triphosphate reductase activating protein | -0.42 | -0.83 | -0.84 | -0.91 | -1.03 | -1.02 |
| O | OEOE_1308 | OEOE_RS06305 | molecular chaperone DnaJ | 1.56 | 1.76 | 1.61 | 1.36 | 1.32 | 1.10 |
| O | OEOE_1309 | OEOE_RS06310 | molecular chaperone DnaK | 1.03 | 1.35 | 1.38 | 1.04 | 1.03 | 0.95 |
| O | OEOE_1310 | OEOE_RS06315 | protein GrpE | 0.93 | 1.41 | 1.59 | 1.11 | 1.02 | 0.94 |
| O | OEOE_1431 | OEOE_RS06910 | trypsin | 0.95 | 1.32 | 1.45 | 1.06 | 0.79 | 0.74 |
| O | OEOE_1554 | OEOE_RS07495 | methionine sulfoxide reductase B | 1.01 | 0.96 | 0.98 | 0.90 | 0.89 | 0.83 |
| O | OEOE_1625 | OEOE_RS07835 | thiol-disulfide isomerase | -1.17 | -1.32 | -1.18 | -1.03 | -1.01 | -1.07 |
| O | OEOE_1630 | OEOE_RS07860 | peptidase M13 | -0.66 | -0.88 | -1.04 | -1.06 | -1.09 | -0.93 |
| O | OEOE_1639 | OEOE_RS07905 | peptidylprolyl isomerase | 2.23 | 2.74 | 2.62 | 2.30 | 2.10 | 1.97 |
| O | OEOE_1702 | OEOE_RS08215 | thioredoxin | -1.10 | -0.71 | -0.71 | -0.70 | -0.68 | -0.81 |
| O | OEOE_1852 | OEOE_RS08940 | osmotically inducible protein C | 1.27 | 0.95 | 0.89 | 0.90 | 0.94 | 1.03 |
| **P: Inorganic ion transport and metabolism** |  |  |  |  |  |  |  |  |  |
| P | OEOE_0172 | OEOE_RS00815 | cobalt ABC transporter permease | 1.03 | 0.95 | 0.66 | 0.76 | 0.76 | 0.74 |
| P | OEOE_0173 | OEOE_RS00820 | cobalt ABC transporter | 1.21 | 1.14 | 0.84 | 0.89 | 0.89 | 0.93 |
| P | OEOE_0246 | OEOE_RS01175 | manganese transporter | -0.61 | -1.12 | -1.29 | -1.37 | -1.37 | -1.30 |
| P | OEOE_0305 | OEOE_RS01455 | sodium:solute symporter | -1.27 | -1.33 | -1.21 | -1.41 | -1.41 | -1.51 |
| P | OEOE_0462 | OEOE_RS02225 | potassium transporter Kef | -2.19 | -2.17 | -1.29 | -0.77 | -0.67 | -0.71 |
| P | OEOE_0623 | OEOE_RS02975 | cobalt ABC transporter | 0.27 | 0.26 | 0.56 | 1.08 | 1.16 | 1.16 |
| P | OEOE_0624 | OEOE_RS02980 | cobalt ABC transporter | 0.35 | 0.42 | 0.64 | 1.10 | 1.23 | 1.24 |
| P | OEOE_0625 | OEOE_RS02985 | cobalt ABC transporter permease | 0.75 | 0.84 | 1.03 | 1.30 | 1.52 | 1.51 |
| P | OEOE_0706 | OEOE_RS03385 | chloride channel protein | 0.64 | 0.56 | 0.88 | 1.07 | 0.93 | 0.97 |
| P | OEOE_0819 | OEOE_RS03935 | MFS transporter | 1.44 | 1.67 | 2.02 | 2.34 | 2.27 | 2.13 |
| P | OEOE_1188 | OEOE_RS05725 | metal ABC transporter substrate-binding protein | -1.11 | -1.33 | -1.48 | -1.72 | -1.64 | -1.70 |
| P | OEOE_1363 | OEOE_RS06560 | ATPase | 1.66 | 1.66 | 1.92 | 1.98 | 2.02 | 2.05 |
| P | OEOE_1533 | OEOE_RS07390 | sodium ABC transporter permease | 1.28 | 1.06 | 0.93 | 1.08 | 1.14 | 1.23 |
| P | OEOE_1534 | OEOE_RS07395 | sodium ABC transporter ATP-binding protein | 1.22 | 0.85 | 0.99 | 1.10 | 1.20 | 1.30 |
| P | OEOE_1540 | OEOE_RS07425 | cobalt ABC transporter ATPase | -0.28 | -0.35 | -0.84 | -1.10 | -1.12 | -1.12 |
| P | OEOE_1679 | OEOE_RS08100 | ammonia permease | 1.28 | 1.61 | 1.57 | 1.46 | 1.35 | 1.33 |
| **Q: Secondary metabolites biosynthesis, transport, and catabolism** |  |  |  |  |  |  |  |  |  |
| Q | OEOE_0064 | OEOE_RS00295 | phytoene synthase | 1.25 | 0.82 | 0.84 | 0.98 | 0.84 | 0.83 |
| Q | OEOE_0804 | OEOE_RS03860 | 4-oxalocrotonate tautomerase | 1.56 | 1.14 | 1.38 | 2.01 | 2.16 | 2.19 |
| **R: General function prediction only** |  |  |  |  |  |  |  |  |  |
| R | OEOE_0025 | OEOE_RS00120 | 2-hydroxyacid dehydrogenase | -0.55 | -0.85 | -1.07 | -1.12 | -1.24 | -1.20 |
| R | OEOE_0036 | OEOE_RS00165 | oxidoreductase ion channel protein IolS | -1.10 | -1.36 | -1.56 | -1.65 | -1.65 | -1.72 |
| R | OEOE_0043 | OEOE_RS00200 | ketosteroid isomerase | -1.05 | -0.92 | -1.13 | -1.21 | -1.05 | -1.10 |
| R | OEOE_0044 | OEOE_RS00205 | oxidoreductase | -0.99 | -0.58 | -0.99 | -1.08 | -1.15 | -1.09 |
| R | OEOE_0070 | OEOE_RS00320 | 2,5-diketo-D-gluconic acid reductase | -0.94 | -1.09 | -1.09 | -1.20 | -1.14 | -1.16 |
| R | OEOE_0071 | OEOE_RS00325 | general stress protein | -2.18 | -2.10 | -2.46 | -2.54 | -2.60 | -2.58 |
| R | OEOE_0076 | OEOE_RS00345 | MFS transporter | 1.04 | 0.72 | 0.67 | 0.69 | 0.69 | 0.50 |
| R | OEOE_0097 | OEOE_RS00445 | MFS transporter | 1.10 | 1.03 | 0.75 | 0.71 | 0.70 | 0.75 |
| R | OEOE_0103 | OEOE_RS00475 | phosphoesterase | 1.56 | 1.30 | 1.15 | 1.28 | 1.26 | 1.30 |
| R | OEOE_0129 | OEOE_RS00600 | gluconate:proton symporter | -1.16 | -1.09 | -1.43 | -1.59 | -1.64 | -1.35 |
| R | OEOE_0137 | OEOE_RS00635 | hemolysin | 1.40 | 1.55 | 1.63 | 1.33 | 1.27 | 1.17 |
| R | OEOE_0148 | OEOE_RS00690 | MFS transporter | 1.91 | 1.98 | 2.18 | 1.96 | 2.00 | 1.96 |
| R | OEOE_0168 | OEOE_RS00795 | acylphosphatase | -1.31 | -1.18 | -1.04 | -0.90 | -0.88 | -0.97 |
| R | OEOE_0174 | OEOE_RS00825 | transporter | 1.01 | 0.79 | 0.70 | 0.75 | 0.76 | 0.81 |
| R | OEOE_0175 | OEOE_RS00830 | transporter | 1.44 | 1.28 | 1.27 | 1.17 | 1.09 | 1.03 |
| R | OEOE_0196 | OEOE_RS00935 | transporter | 1.54 | 1.05 | 1.05 | 1.05 | 1.05 | 1.05 |
| R | OEOE_0241 | OEOE_RS01150 | alpha/β hydrolase | -0.72 | -0.53 | -0.76 | -0.93 | -1.07 | -1.10 |
| R | OEOE_0242 | OEOE_RS01155 | 3-β-hydroxysteroid dehydrogenase | -0.84 | -1.07 | -1.38 | -1.48 | -1.50 | -1.52 |
| R | OEOE_0243 | OEOE_RS01160 | oxidoreductase ion channel protein IolS | -1.16 | -1.03 | -1.07 | -1.21 | -1.07 | -1.09 |
| R | OEOE_0244 | OEOE_RS01165 | DNA-binding protein | -1.71 | -1.67 | -1.96 | -2.01 | -1.99 | -2.00 |
| R | OEOE_0265 | OEOE_RS01270 | MFS transporter | -0.83 | -1.11 | -1.17 | -1.28 | -1.49 | -1.41 |
| R | OEOE_0290 | OEOE_RS01390 | MFS transporter permease | 1.56 | 1.51 | 1.53 | 1.59 | 1.53 | 1.79 |
| R | OEOE_0345 | OEOE_RS01655 | acetyltransferase | -1.27 | -1.02 | -1.29 | -1.40 | -1.27 | -1.27 |
| R | OEOE_0384 | OEOE_RS01845 | MFS transporter permease | 0.76 | 1.11 | 1.24 | 1.33 | 1.27 | 1.34 |
| R | OEOE_0396 | OEOE_RS01905 | carbonyl reductase | 0.72 | 0.86 | 0.75 | 0.89 | 1.05 | 1.20 |
| R | OEOE_0408 | OEOE_RS01960 | oxidoreductase | -1.05 | -1.06 | -1.21 | -1.25 | -1.14 | -1.18 |
| R | OEOE_0416 | OEOE_RS02000 | glucosaminidase | 1.09 | 1.07 | 0.90 | 0.79 | 0.70 | 0.80 |
| R | OEOE_0490 | OEOE_RS02340 | FMN-binding protein | -0.74 | -0.67 | -0.66 | -0.90 | -0.91 | -1.02 |
| R | OEOE_0503 | OEOE_RS02400 | MFS transporter | 1.46 | 1.20 | 1.06 | 1.22 | 1.21 | 1.23 |
| R | OEOE_0693 | OEOE_RS03325 | acetoin reductase | -0.96 | -1.32 | -1.53 | -1.80 | -1.82 | -2.01 |
| R | OEOE_0705 | OEOE_RS03380 | MFS transporter | 1.30 | 1.42 | 1.76 | 1.73 | 1.63 | 1.63 |
| R | OEOE_0757 | OEOE_RS03625 | short-chain dehydrogenase | -0.57 | -0.67 | -0.95 | -1.12 | -1.11 | -1.16 |
| R | OEOE_0784 | OEOE_RS03755 | metallophosphatase | -1.07 | -1.14 | -1.18 | -1.16 | -1.13 | -1.13 |
| R | OEOE_0863 | OEOE_RS04145 | acyltransferase | 1.03 | 1.90 | 2.28 | 1.87 | 1.73 | 1.70 |
| R | OEOE_0868 | OEOE_RS04165 | acetyltransferase | 1.32 | 1.29 | 1.57 | 1.48 | 1.43 | 1.37 |
| R | OEOE_0869 | OEOE_RS04170 | MFS transporter | 1.24 | 1.14 | 1.24 | 1.47 | 1.31 | 1.39 |
| R | OEOE_0881 | OEOE_RS04220 | ACP phosphodiesterase | 0.58 | 1.35 | 1.51 | 0.58 | 0.31 | 0.01 |
| R | OEOE_0886 | OEOE_RS04245 | oxalate:formate antiporter | -1.39 | -1.93 | -2.06 | -2.23 | -2.27 | -2.33 |
| R | OEOE_0896 | OEOE_RS04295 | adenylyltransferase | -0.44 | -0.66 | -0.81 | -1.06 | -0.94 | -0.90 |
| R | OEOE_0927 | OEOE_RS04445 | acetyltransferase | 1.20 | 1.07 | 1.56 | 1.88 | 1.77 | 1.83 |
| R | OEOE_0938 | OEOE_RS04495 | short-chain dehydrogenase | -0.99 | -1.48 | -1.51 | -1.38 | -1.40 | -1.45 |
| R | OEOE_0989 | OEOE_RS04750 | GTPase Era | -1.23 | -1.50 | -1.19 | -0.95 | -1.10 | -1.11 |
| R | OEOE_0997 | OEOE_RS04790 | SAM-dependent methyltransferase | 0.99 | 1.05 | 1.31 | 1.56 | 1.73 | 1.55 |
| R | OEOE_1018 | OEOE_RS04890 | GTPase | 0.50 | 0.84 | 1.07 | 0.95 | 0.90 | 0.88 |
| R | OEOE_1080 | OEOE_RS05190 | pore-forming protein | 0.77 | 0.95 | 1.13 | 1.21 | 1.39 | 1.45 |
| R | OEOE_1202 | OEOE_RS05795 | general stress protein | -0.74 | -1.00 | -1.03 | -0.91 | -0.82 | -0.86 |
| R | OEOE_1260 | OEOE_RS06075 | SAM-dependent methyltransferase | 0.88 | 1.05 | 1.16 | 1.29 | 1.40 | 1.37 |
| R | OEOE_1322 | OEOE_RS06370 | amidophosphoribosyltransferase | 1.14 | 0.71 | 0.78 | 0.84 | 0.96 | 0.81 |
| R | OEOE_1347 | OEOE_RS06475 | acetyltransferase | -1.08 | -1.22 | -1.14 | -1.23 | -1.19 | -1.12 |
| R | OEOE_1364 | OEOE_RS06565 | acyltransferase | -1.53 | -1.90 | -1.91 | -1.57 | -1.42 | -1.18 |
| R | OEOE_1412 | OEOE_RS06805 | haloacid dehalogenase | -0.89 | -0.91 | -1.03 | -1.00 | -1.20 | -1.35 |
| R | OEOE_1424 | OEOE_RS06865 | competence protein ComX | 1.67 | 1.81 | 1.76 | 1.62 | 1.64 | 1.52 |
| R | OEOE_1474 | OEOE_RS07115 | glycosyl transferase | 1.19 | 0.77 | 0.60 | 0.62 | 0.62 | 0.59 |
| R | OEOE_1514 | OEOE_RS07295 | glycosyl transferase | 0.99 | 0.87 | 0.77 | 0.94 | 1.01 | 1.13 |
| R | OEOE_1536 | OEOE_RS07405 | MFS transporter | 0.76 | 0.62 | 0.74 | 1.12 | 1.31 | 1.44 |
| R | OEOE_1560 | OEOE_RS07525 | 17-β-hydroxysteroid dehydrogenase | -0.45 | -0.85 | -1.01 | -1.12 | -1.21 | -1.13 |
| R | OEOE_1607 | OEOE_RS07750 | heme ABC transporter ATP-binding protein | -0.42 | -0.85 | -1.14 | -1.36 | -1.37 | -1.37 |
| R | OEOE_1608 | OEOE_RS07755 | ABC transporter permease | -0.29 | -0.52 | -1.00 | -1.25 | -1.23 | -1.24 |
| R | OEOE_1748 | OEOE_RS08435 | HD family phosphohydrolase | -1.22 | -0.86 | -1.05 | -0.90 | -0.78 | -0.88 |
| R | OEOE_1782 | OEOE_RS08590 | aldo/keto reductase | -1.16 | -1.31 | -1.60 | -1.87 | -1.89 | -1.88 |
| R | OEOE_1820 | OEOE_RS08780 | acetyltransferase | 0.87 | 0.81 | 0.89 | 0.98 | 1.12 | 1.03 |
| R | OEOE_1841 | OEOE_RS08890 | CAAX amino protease | 1.02 | 0.81 | 0.73 | 0.72 | 0.68 | 0.65 |
| R | OEOE_1843 | OEOE_RS08900 | permease | 1.57 | 1.39 | 1.39 | 1.48 | 1.44 | 1.63 |
| R | OEOE_1851 | OEOE_RS08935 | O-acetyltransferase | 1.04 | 0.81 | 0.73 | 0.81 | 0.84 | 0.81 |
| R | OEOE_1858 | OEOE_RS08970 | 2,5-diketo-D-gluconic acid reductase | -0.81 | -0.61 | -0.97 | -1.22 | -1.28 | -1.20 |
| R | OEOE_1859 | OEOE_RS08975 | oxidoreductase | 1.15 | 1.47 | 1.37 | 1.14 | 1.15 | 1.04 |
| **S: Function unknown** |  |  |  |  |  |  |  |  |  |
| S | OEOE_0069 | OEOE_RS00315 | membrane protein | -1.66 | -1.64 | -1.84 | -1.95 | -2.01 | -2.19 |
| S | OEOE_0406 | OEOE_RS01950 | membrane protein | 1.70 | 1.87 | 1.62 | 1.48 | 1.54 | 1.50 |
| S | OEOE_0483 | OEOE_RS02305 | membrane protein | -2.20 | -2.54 | -2.49 | -2.61 | -2.59 | -2.99 |
| S | OEOE_0484 | OEOE_RS02310 | cytochrome O ubiquinol oxidase | -2.05 | -2.42 | -2.40 | -2.59 | -2.40 | -2.69 |
| S | OEOE_0547 | OEOE_RS02595 | amidase | 1.16 | 1.10 | 1.23 | 1.08 | 1.15 | 1.03 |
| S | OEOE_0634 | OEOE_RS03030 | membrane protein | 1.44 | 1.24 | 1.37 | 1.84 | 1.75 | 1.83 |
| S | OEOE_0639 | OEOE_RS03055 | membrane protein | -1.49 | -1.56 | -1.63 | -1.57 | -1.51 | -1.51 |
| S | OEOE_0650 | OEOE_RS03110 | membrane protein | 1.59 | 1.71 | 1.72 | 1.67 | 1.72 | 1.67 |
| S | OEOE_0713 | OEOE_RS03410 | membrane protein | 2.72 | 3.21 | 3.72 | 3.61 | 3.47 | 3.40 |
| S | OEOE_0753 | OEOE_RS03605 | membrane protein | 1.82 | 2.06 | 2.07 | 2.14 | 2.18 | 2.15 |
| S | OEOE_0835 | OEOE_RS04015 | membrane protein | 1.72 | 1.42 | 1.72 | 2.02 | 1.83 | 1.81 |
| S | OEOE_0930 | OEOE_RS04465 | membrane protein | 2.28 | 2.77 | 3.32 | 3.10 | 2.70 | 2.56 |
| S | OEOE_0933 | OEOE_RS04470 | 5'-3'-deoxyribonucleotidase | 1.24 | 1.52 | 1.81 | 2.38 | 2.94 | 3.25 |
| S | OEOE_1035 | OEOE_RS04975 | membrane protein | -1.30 | -1.71 | -1.80 | -1.76 | -1.73 | -1.88 |
| S | OEOE_1109 | OEOE_RS05330 | membrane protein | -1.23 | -0.96 | -0.66 | -0.46 | -0.49 | -0.52 |
| S | OEOE_1229 | OEOE_RS05920 | cytochrome O ubiquinol oxidase | 0.46 | 0.45 | 0.67 | 0.90 | 0.94 | 1.06 |
| S | OEOE_1354 | OEOE_RS06515 | membrane protein | -1.33 | -1.55 | -1.28 | -1.07 | -0.89 | -1.14 |
| S | OEOE_1367 | OEOE_RS06575 | membrane protein | -0.40 | -0.92 | -1.20 | -0.99 | -0.92 | -1.08 |
| S | OEOE_1472 | OEOE_RS07105 | membrane protein | 0.96 | 1.07 | 0.62 | 0.55 | 0.60 | 0.70 |
| S | OEOE_1567 | OEOE_RS07560 | membrane protein | 1.49 | 1.31 | 1.08 | 1.04 | 0.98 | 1.07 |
| S | OEOE_1606 | OEOE_RS07745 | membrane protein | -0.98 | -1.08 | -1.46 | -1.74 | -1.69 | -1.74 |
| S | OEOE_1620 | OEOE_RS07815 | membrane protein | -0.36 | 0.06 | 0.54 | 0.97 | 1.00 | 0.54 |
| S | OEOE_1829 | OEOE_RS08830 | glycosyl transferase | 1.38 | 1.07 | 0.96 | 1.07 | 1.07 | 1.12 |
| **T: Signal transduction mechanisms** |  |  |  |  |  |  |  |  |  |
| T | OEOE_0120 | OEOE_RS00555 | histidine kinase | -1.07 | -1.18 | -1.28 | -1.14 | -1.17 | -1.14 |
| T | OEOE_0143 | OEOE_RS00665 | histidine kinase | 0.82 | 0.93 | 1.02 | 1.13 | 1.01 | 0.96 |
| T | OEOE_0420 | OEOE_RS02020 | [citrate [pro-3S]-lyase] ligase | 1.85 | 2.22 | 1.77 | 1.23 | 1.20 | 1.17 |
| T | OEOE_0421 | OEOE_RS02025 | citrate lyase ACP | 1.68 | 1.96 | 1.88 | 1.44 | 1.24 | 1.45 |
| T | OEOE_0422 | OEOE_RS02030 | citrate lyase | 1.25 | 1.50 | 1.25 | 0.95 | 0.91 | 1.07 |
| T | OEOE_0489 | OEOE_RS02335 | histidine kinase | -0.75 | -0.84 | -1.22 | -1.32 | -1.24 | -1.23 |
| **U: Intracellular trafficking, secretion, and vesicular transport** |  |  |  |  |  |  |  |  |  |
| U | OEOE_0614 | OEOE_RS02940 | preprotein translocase subunit SecY | 1.20 | 1.05 | 1.32 | 1.62 | 1.56 | 1.50 |
| U | OEOE_0794 | OEOE_RS03805 | type VI secretion protein ImpB | 0.63 | 0.98 | 1.01 | 1.06 | 1.13 | 1.31 |
| U | OEOE_1254 | OEOE_RS06045 | type II secretion protein | 1.21 | 0.82 | 0.61 | 0.60 | 0.61 | 0.49 |
| U | OEOE_1255 | OEOE_RS06050 | competence protein ComGC | 1.08 | 0.67 | 0.45 | 0.44 | 0.46 | 0.17 |
| U | OEOE_1256 | OEOE_RS06055 | type II secretion system protein F | 1.35 | 0.80 | 0.66 | 0.56 | 0.58 | 0.47 |
| U | OEOE_1862 | OEOE_RS08990 | membrane protein | 1.18 | 1.52 | 1.70 | 1.68 | 1.59 | 1.53 |
| **V: Defense mechanisms** |  |  |  |  |  |  |  |  |  |
| V | OEOE_0439 | OEOE_RS02115 | multidrug ABC transporter ATP-binding protein | 3.69 | 4.43 | 4.75 | 4.45 | 4.26 | 4.08 |
| V | OEOE_0512 | OEOE_RS02435 | diguanylate cyclase | 1.23 | 1.22 | 0.99 | 1.08 | 1.04 | 1.15 |
| V | OEOE_0588 | OEOE_RS02805 | N-acetylmuramoyl-L-alanine amidase | 1.85 | 2.06 | 2.14 | 2.01 | 1.79 | 1.71 |
| V | OEOE_0720 | OEOE_RS03440 | acetyl esterase | 3.23 | 3.72 | 4.21 | 4.03 | 3.69 | 3.61 |
| V | OEOE_0722 | OEOE_RS03445 | multidrug ABC transporter ATPase | 1.72 | 1.69 | 2.02 | 2.16 | 1.93 | 2.02 |
| V | OEOE_0723 | OEOE_RS03450 | multidrug ABC transporter permease | 2.15 | 2.01 | 2.34 | 2.48 | 2.35 | 2.18 |
| V | OEOE_0737 | OEOE_RS03520 | multidrug ABC transporter permease | -0.79 | -0.88 | -0.96 | -0.98 | -1.06 | -1.01 |
| V | OEOE_0761 | OEOE_RS03640 | multidrug ABC transporter permease | 0.88 | 1.23 | 1.10 | 1.03 | 1.06 | 1.17 |
| V | OEOE_0877 | OEOE_RS04200 | multidrug MFS transporter | 2.39 | 2.77 | 3.14 | 3.51 | 3.73 | 3.75 |
| V | OEOE_1345 | OEOE_RS06465 | multidrug ABC transporter permease | -0.50 | -0.52 | -0.99 | -1.21 | -1.29 | -0.94 |
| V | OEOE_1636 | OEOE_RS07890 | multidrug ABC transporter ATP-binding protein | 1.07 | 0.93 | 0.72 | 0.69 | 0.59 | 0.65 |
| V | OEOE_1711 | OEOE_RS08260 | multidrug ABC transporter ATP-binding protein | 1.48 | 1.54 | 1.68 | 1.80 | 1.47 | 1.05 |
| V | OEOE_1712 | OEOE_RS08265 | multidrug ABC transporter ATP-binding protein | 1.01 | 1.04 | 1.25 | 1.40 | 1.10 | 0.76 |
| **Multi COGs** |  |  |  |  |  |  |  |  |  |
| A J K F | OEOE_1643 | OEOE_RS07925 | DEAD/DEAH box helicase | 2.15 | 2.46 | 2.37 | 2.47 | 2.47 | 2.37 |
| C H | OEOE_0769 | OEOE_RS03675 | formate--tetrahydrofolate ligase | 1.02 | 1.45 | 1.27 | 1.25 | 1.30 | 1.32 |
| C O | OEOE_0414 | OEOE_RS01990 | cysteine ABC transporter ATP-binding protein | -0.62 | -0.86 | -1.01 | -1.16 | -1.23 | -1.25 |
| C R | OEOE_1358 | OEOE_RS06535 | NADPH:quinone reductase | -0.74 | -1.14 | -1.03 | -0.99 | -0.94 | -0.99 |
| E F | OEOE_0260 | OEOE_RS01245 | carbamoyl phosphate synthase small subunit | -1.08 | -1.62 | -1.82 | -2.07 | -2.28 | -2.24 |
| E C G T | OEOE_0952 | OEOE_RS04565 | glutamine synthetase | 1.88 | 2.08 | 2.20 | 2.01 | 1.83 | 1.81 |
| E F | OEOE_0261 | OEOE_RS01250 | carbamoyl-phosphate synthase large chain | -0.66 | -0.89 | -1.18 | -1.54 | -1.69 | -1.78 |
| E G | OEOE_0387 | OEOE_RS01860 | 4-aminobutyrate aminotransferase | 2.98 | 3.30 | 3.27 | 3.20 | 3.21 | 3.30 |
| E G | OEOE_0638 | OEOE_RS03050 | phosphoglycerate kinase | -1.12 | -1.25 | -1.39 | -1.29 | -1.22 | -1.22 |
| E G V | OEOE_0635 | OEOE_RS03035 | glucosamine--fructose-6-phosphate aminotransferase | 2.94 | 3.14 | 3.65 | 3.92 | 3.99 | 3.97 |
| E H Q V | OEOE_1296 | OEOE_RS06245 | branched-chain amino acid aminotransferase | 0.95 | 1.42 | 2.04 | 2.25 | 2.61 | 3.02 |
| E M S | OEOE_1644 | OEOE_RS07930 | UDP-N-acetylmuramoyl-tripeptide--D-alanyl-D-alanine ligase | 1.30 | 1.50 | 1.29 | 1.29 | 1.29 | 1.29 |
| E Q | OEOE_0766 | OEOE_RS03660 | cystathionine β-lyase | 0.82 | 1.15 | 0.81 | 0.69 | 0.74 | 0.77 |
| E Q | OEOE_0895 | OEOE_RS04290 | aspartate aminotransferase | 1.42 | 1.89 | 1.99 | 2.06 | 1.85 | 2.02 |
| E Q | OEOE_1048 | OEOE_RS05035 | aspartate racemase | -0.61 | -0.81 | -1.08 | -1.10 | -1.13 | -0.99 |
| E R | OEOE_0394 | OEOE_RS01895 | threonine dehydrogenase | -0.99 | -1.29 | -1.15 | -0.99 | -0.96 | -0.87 |
| E R | OEOE_1707 | OEOE_RS08240 | alcohol dehydrogenase | -0.41 | -0.77 | -1.02 | -1.14 | -1.27 | -1.30 |
| E R | OEOE_1780 | OEOE_RS08580 | alcohol dehydrogenase | -0.66 | -1.31 | -1.40 | -1.50 | -1.64 | -1.68 |
| E V C G | OEOE_0122 | OEOE_RS00565 | phosphoglyceromutase | -1.37 | -1.60 | -1.51 | -1.40 | -1.37 | -1.42 |
| E V Q | OEOE_0152 | OEOE_RS00715 | 3-phosphoshikimate 1-carboxyvinyltransferase | 1.71 | 1.92 | 1.62 | 1.49 | 1.64 | 1.95 |
| E V Q | OEOE_0153 | OEOE_RS00720 | shikimate kinase | 2.12 | 2.16 | 2.08 | 1.92 | 2.08 | 2.26 |
| E V Q | OEOE_0939 | OEOE_RS04500 | argininosuccinate synthase | -0.20 | -0.58 | -0.82 | -0.94 | -1.10 | -1.01 |
| E V Q | OEOE_1546 | OEOE_RS07455 | homoserine dehydrogenase | 1.40 | 1.52 | 1.58 | 1.65 | 1.59 | 1.74 |
| E V Q C G | OEOE_0310 | OEOE_RS01480 | phosphoglycerate mutase | 0.51 | 0.73 | 1.22 | 1.11 | 0.77 | 0.82 |
| E V Q C G | OEOE_1611 | OEOE_RS07770 | ribose 5-phosphate isomerase | -0.87 | -0.94 | -1.09 | -1.37 | -1.27 | -1.16 |
| E V Q C G | OEOE_1652 | OEOE_RS07970 | triosephosphate isomerase | -1.34 | -1.12 | -1.25 | -1.55 | -1.51 | -1.67 |
| E V Q G C O | OEOE_1650 | OEOE_RS07960 | enolase | -0.76 | -0.76 | -0.75 | -1.06 | -1.07 | -1.12 |
| F E | OEOE_0163 | OEOE_RS00770 | ferredoxin--NADP reductase | 0.93 | 1.14 | 1.24 | 1.35 | 1.39 | 1.35 |
| F E | OEOE_0258 | OEOE_RS01235 | aspartate carbamoyltransferase | -1.14 | -1.75 | -2.00 | -2.27 | -2.56 | -2.50 |
| F E | OEOE_0566 | OEOE_RS02695 | thioredoxin reductase | 1.06 | 1.66 | 1.60 | 1.23 | 1.20 | 1.23 |
| F E V Q | OEOE_1125 | OEOE_RS05410 | adenylosuccinate lyase | -0.42 | -0.88 | -0.98 | -1.16 | -1.06 | -0.94 |
| F G R | OEOE_0929 | OEOE_RS04460 | HIT family hydrolases | 2.67 | 3.22 | 3.98 | 3.81 | 3.44 | 3.28 |
| F H | OEOE_1469 | OEOE_RS07095 | allantoin permease | -0.95 | -0.85 | -1.12 | -1.24 | -1.14 | -1.01 |
| F Q | OEOE_0537 | OEOE_RS02555 | xanthine phosphoribosyltransferase | -1.08 | -1.09 | -0.82 | -0.28 | -0.07 | -0.03 |
| F V Q | OEOE_0615 | OEOE_RS02945 | adenylate kinase | 1.21 | 1.08 | 1.22 | 1.36 | 1.44 | 1.22 |
| G C T | OEOE_0418 | OEOE_RS02010 | malate dehydrogenase | 2.20 | 2.51 | 2.25 | 1.77 | 1.68 | 1.61 |
| G I | OEOE_1781 | OEOE_RS08585 | alpha-galactosidase | -1.21 | -1.24 | -1.58 | -1.92 | -2.08 | -2.06 |
| G Q | OEOE_0249 | OEOE_RS01190 | mannose-6-phosphate isomerase | -1.06 | -1.41 | -1.55 | -1.41 | -1.24 | -1.26 |
| G T | OEOE_0544 | OEOE_RS02580 | hydrolase | 1.73 | 1.89 | 1.49 | 1.32 | 1.22 | 1.29 |
| G V | OEOE_1542 | OEOE_RS07435 | bifunctional protein GlmU | -0.61 | -1.02 | -1.03 | -1.15 | -1.16 | -1.10 |
| G V Q C | OEOE_0920 | OEOE_RS04415 | glucokinase | -0.95 | -1.18 | -1.20 | -0.95 | -0.80 | -0.57 |
| H R | OEOE_0700 | OEOE_RS03355 | alpha/β hydrolase | -1.62 | -2.02 | -1.82 | -1.54 | -1.30 | -1.34 |
| H R | OEOE_0836 | OEOE_RS04020 | haloacid dehalogenase | 1.37 | 1.20 | 1.27 | 1.42 | 1.43 | 1.34 |
| H R | OEOE_1196 | OEOE_RS05765 | haloacid dehalogenase | 0.54 | 0.65 | 0.78 | 0.88 | 0.77 | 1.05 |
| H R | OEOE_1845 | OEOE_RS08910 | haloacid dehalogenase | 1.31 | 1.34 | 1.03 | 0.98 | 1.04 | 1.04 |
| I H | OEOE_1586 | OEOE_RS07650 | 3-hydroxyacyl-ACP dehydratase | 0.33 | 0.28 | 0.70 | 0.91 | 0.94 | 1.03 |
| J O | OEOE_0717 | OEOE_RS03425 | spermidine N1-acetyltransferase | 0.36 | 1.00 | 1.28 | 1.22 | 1.00 | 1.00 |
| L F | OEOE_1663 | OEOE_RS08025 | DNA polymerase III subunit alpha | 1.18 | 1.21 | 0.89 | 0.80 | 0.89 | 0.89 |
| L O | OEOE_1192 | OEOE_RS05745 | ATPase AAA | -1.08 | -1.15 | -1.21 | -1.20 | -1.20 | -1.08 |
| M G | OEOE_1649 | OEOE_RS07955 | saccharopine dehydrogenase | -0.71 | -0.75 | -0.96 | -1.03 | -1.12 | -1.17 |
| M G I | OEOE_1044 | OEOE_RS05015 | β-galactosidase | -0.96 | -1.45 | -1.54 | -1.45 | -1.33 | -1.27 |
| M V | OEOE_0900 | OEOE_RS04315 | carboxypeptidase | 1.16 | 1.45 | 1.15 | 1.15 | 1.15 | 1.15 |
| Q F | OEOE_1127 | OEOE_RS05420 | inosine-5-monophosphate dehydrogenase | -0.74 | -1.14 | -1.14 | -1.27 | -1.27 | -1.31 |
| Q G I V | OEOE_1248 | OEOE_RS06015 | alcohol dehydrogenase | -0.51 | -0.76 | -1.09 | -1.46 | -1.61 | -1.58 |
| Q G I V | OEOE_1330 | OEOE_RS06400 | alcohol dehydrogenase | -0.64 | -1.02 | -1.06 | -1.10 | -1.24 | -1.30 |
| Q H | OEOE_0279 | OEOE_RS01335 | dihydroxynaphthoic acid synthetase | -0.60 | -0.91 | -0.93 | -0.92 | -0.98 | -1.15 |
| Q H | OEOE_0280 | OEOE_RS01340 | acyl-CoA synthetase | -0.85 | -0.98 | -1.12 | -1.24 | -1.25 | -1.34 |
| Q V | OEOE_1100 | OEOE_RS05285 | kinase | -1.13 | -1.09 | -0.88 | -0.83 | -0.84 | -0.84 |
| Q V | OEOE_1445 | OEOE_RS06980 | dTDP-4-dehydrorhamnose reductase | 2.21 | 2.97 | 3.26 | 3.05 | 2.78 | 2.71 |
| Q V | OEOE_1447 | OEOE_RS06990 | dTDP-glucose 4,6-dehydratase | -0.72 | -0.89 | -1.06 | -1.35 | -1.38 | -1.23 |
| Q V | OEOE_1448 | OEOE_RS06995 | dTDP-4-dehydrorhamnose 3,5-epimerase | -0.55 | -0.72 | -0.88 | -1.09 | -1.18 | -1.12 |
| Q V | OEOE_1449 | OEOE_RS07000 | glucose-1-phosphate thymidylyltransferase | -0.62 | -0.95 | -1.09 | -1.36 | -1.32 | -1.25 |
| T K | OEOE_0142 | OEOE_RS00660 | transcriptional regulator | 1.18 | 1.67 | 1.99 | 2.17 | 1.93 | 1.67 |
| T K | OEOE_0488 | OEOE_RS02330 | PhoB family transcriptional regulator | -0.77 | -1.03 | -1.18 | -1.21 | -1.14 | -1.23 |
| T S | OEOE_0214 | OEOE_RS01015 | D-alanyl-D-alanine dipeptidase | -0.75 | -0.46 | -0.81 | -1.02 | -1.33 | -0.99 |
| V Q C E G | OEOE_0135 | OEOE_RS00625 | glucose-6-phosphate 1-dehydrogenase | -0.53 | -0.86 | -1.02 | -1.17 | -1.26 | -1.31 |
| V Q C E G | OEOE_0892 | OEOE_RS04275 | 6-phosphogluconate dehydrogenase | -0.60 | -0.73 | -1.07 | -1.38 | -1.43 | -1.44 |
| V Q C E G | OEOE_1523 | OEOE_RS07345 | 6-phosphogluconate dehydrogenase | -0.79 | -1.25 | -1.35 | -1.57 | -1.76 | -1.73 |
| V Q C G | OEOE_0328 | OEOE_RS01570 | pyruvate dehydrogenase E1 subunit alpha | -0.58 | -0.90 | -0.93 | -1.22 | -1.20 | -0.90 |
| V Q C G | OEOE_0329 | OEOE_RS01575 | 2-oxoisovalerate dehydrogenase subunit β | -0.41 | -0.59 | -0.70 | -1.08 | -1.03 | -0.75 |
| V Q C G | OEOE_0330 | OEOE_RS01580 | dihydrolipoamide acetyltransferase | -0.61 | -0.60 | -0.85 | -1.25 | -1.15 | -0.97 |
| V Q C G E | OEOE_0331 | OEOE_RS01585 | dihydrolipoamide dehydrogenase | -0.66 | -0.61 | -0.84 | -1.16 | -1.11 | -0.90 |
| V Q F | OEOE_1136 | OEOE_RS05465 | phosphoribosylaminoimidazole-succinocarboxamide synthase | -0.19 | -0.68 | -0.96 | -1.09 | -1.25 | -1.44 |
| V Q F | OEOE_1137 | OEOE_RS05470 | phosphoribosylaminoimidazole carboxylase | -0.13 | -0.60 | -0.67 | -0.89 | -1.02 | -0.97 |
| V Q F | OEOE_1138 | OEOE_RS05475 | N5-carboxyaminoimidazole ribonucleotide mutase | -1.03 | -1.33 | -1.42 | -1.61 | -1.64 | -1.66 |
| V Q G | OEOE_0301 | OEOE_RS01435 | aldose 1-epimerase | -1.52 | -1.02 | -1.55 | -1.77 | -1.76 | -1.85 |
| V Q H F | OEOE_1129 | OEOE_RS05430 | bifunctional purine biosynthesis protein PurH | -0.16 | -0.37 | -0.70 | -1.03 | -1.10 | -1.07 |
| V Q H F | OEOE_1130 | OEOE_RS05435 | phosphoribosylglycinamide formyltransferase | -0.11 | -0.26 | -0.62 | -0.95 | -1.05 | -1.05 |
| V T | OEOE_0164 | OEOE_RS00775 | diguanylate cyclase | 0.96 | 1.20 | 1.44 | 1.51 | 1.60 | 1.56 |
