# Supplementary material for: Transcriptomic and Proteomic Analysis of Oenococcus oeni Adaptation to Wine Stress Conditions
Source: Front Microbiol. 2016 Sep 30;7:1554. doi: 10.3389/fmicb.2016.01554 (PMC5044463; doi:10.3389/fmicb.2016.01554)
Supplement: Supplementary file 2 [file Table2.docx]

**Table S2.** Over-expressed and under-expressed genes annotated as hypothetic proteins, during acclimation to WLM. Time samples with over- or under-expression are grey highlighted.

|  | **Relative expression in function of time (h)** | | | |  |  |
| --- | --- | --- | --- | --- | --- | --- |
|  | **0.5** | **1** | **2** | **4** | **6** | **8** |
| **Over-expressed genes** |  |  |  |  |  |  |
| OEOE_0013 | 1.26 | 1.39 | 1.60 | 1.60 | 1.42 | 1.39 |
| OEOE_0063 | 1.31 | 1.03 | 1.23 | 1.38 | 1.23 | 1.25 |
| OEOE_0085 | 0.91 | 1.04 | 0.91 | 0.89 | 1.00 | 0.91 |
| OEOE_0086 | 1.10 | 0.89 | 0.89 | 0.89 | 0.89 | 0.88 |
| OEOE_0151 | 1.38 | 1.69 | 1.63 | 1.57 | 1.69 | 2.11 |
| OEOE_0177 | 0.95 | 1.41 | 1.17 | 0.89 | 0.71 | 0.77 |
| OEOE_0178 | 1.44 | 1.78 | 2.09 | 1.86 | 1.56 | 1.42 |
| OEOE_0180 | 0.60 | 1.16 | 0.75 | 0.40 | 0.25 | 0.21 |
| OEOE_0250 | 1.41 | 0.83 | 0.70 | 0.82 | 0.86 | 0.84 |
| OEOE_0275 | 1.25 | 1.19 | 1.42 | 1.44 | 1.77 | 2.41 |
| OEOE_0386 | 2.71 | 3.31 | 3.34 | 3.39 | 3.39 | 3.43 |
| OEOE_0389 | 1.93 | 1.93 | 2.01 | 2.06 | 1.76 | 2.04 |
| OEOE_0392 | 1.88 | 1.76 | 1.95 | 1.76 | 1.76 | 1.76 |
| OEOE_0399 | 1.17 | 0.94 | 0.78 | 0.83 | 0.79 | 0.83 |
| OEOE_0402 | 0.74 | 1.22 | 1.36 | 1.23 | 1.10 | 1.10 |
| OEOE_0431 | 1.43 | 1.32 | 1.43 | 1.49 | 1.50 | 1.64 |
| OEOE_0502 | 1.16 | 0.81 | 0.79 | 0.81 | 0.81 | 0.81 |
| OEOE_0509 | 1.66 | 2.15 | 2.48 | 2.74 | 2.49 | 2.66 |
| OEOE_0546 | 1.29 | 1.14 | 1.31 | 1.20 | 1.20 | 1.20 |
| OEOE_0568 | 0.84 | 1.08 | 1.04 | 0.97 | 0.88 | 0.98 |
| OEOE_0651 | 0.81 | 1.09 | 1.14 | 1.24 | 1.28 | 1.15 |
| OEOE_0691 | 0.74 | 1.59 | 2.50 | 1.91 | 1.36 | 1.12 |
| OEOE_0692 | 0.05 | 0.76 | 1.42 | 0.98 | 0.58 | 0.35 |
| OEOE_0715 | 2.12 | 2.50 | 2.79 | 2.68 | 2.57 | 2.50 |
| OEOE_0760 | 1.23 | 1.78 | 1.68 | 1.49 | 1.51 | 1.65 |
| OEOE_0817 | 1.74 | 1.99 | 2.59 | 2.70 | 2.69 | 2.71 |
| OEOE_0818 | 0.16 | 0.11 | 0.81 | 1.18 | 1.23 | 1.19 |
| OEOE_0837 | 1.39 | 1.32 | 1.21 | 1.37 | 1.36 | 1.29 |
| OEOE_0874 | 1.19 | 1.21 | 2.00 | 2.26 | 2.48 | 2.17 |
| OEOE_0876 | 2.73 | 3.10 | 3.33 | 3.69 | 3.82 | 3.78 |
| OEOE_0925 | 0.49 | 0.50 | 1.04 | 1.44 | 1.38 | 1.22 |
| OEOE_0944 | 1.04 | 1.00 | 1.04 | 0.98 | 0.98 | 0.97 |
| OEOE_0945 | 0.95 | 1.02 | 0.81 | 0.70 | 0.59 | 0.57 |
| OEOE_0961 | 2.85 | 2.59 | 2.37 | 2.57 | 2.75 | 2.79 |
| OEOE_0971 | 1.11 | 0.98 | 0.91 | 0.91 | 0.91 | 0.78 |
| OEOE_1057 | 1.16 | 0.41 | 0.69 | 0.76 | 0.83 | 0.85 |
| OEOE_1161 | 1.58 | 1.36 | 1.09 | 0.95 | 0.99 | 0.94 |
| OEOE_1362 | 0.30 | 0.23 | 0.68 | 0.87 | 1.04 | 1.02 |
| OEOE_1490 | -0.28 | -0.28 | -0.29 | 0.48 | 0.84 | 1.07 |
| OEOE_1521 | 1.28 | 1.09 | 1.08 | 1.24 | 1.29 | 1.39 |
| OEOE_1522 | 1.14 | 0.83 | 0.75 | 0.88 | 0.91 | 0.93 |
| OEOE_1526 | 0.62 | 1.14 | 1.02 | 0.90 | 0.83 | 0.76 |
| OEOE_1548 | 0.71 | 1.24 | 1.23 | 1.20 | 1.22 | 0.98 |
| OEOE_1683 | 0.49 | 0.12 | 0.40 | 1.20 | 1.95 | 2.42 |
| OEOE_1684 | 1.12 | 1.41 | 1.73 | 1.71 | 1.77 | 1.66 |
| OEOE_1718 | 1.51 | 1.44 | 1.41 | 1.67 | 1.79 | 1.82 |
| OEOE_1789 | 0.96 | 0.96 | 0.96 | 1.06 | 1.08 | 0.94 |
| OEOE_1810 | 1.21 | 1.33 | 1.54 | 2.00 | 2.07 | 2.09 |
| OEOE_1817 | 1.02 | 0.75 | 0.61 | 0.58 | 0.63 | 0.63 |
| OEOE_1844 | 1.40 | 1.33 | 1.37 | 1.38 | 1.38 | 1.47 |
| OEOE_1850 | 1.29 | 0.90 | 0.90 | 0.90 | 0.82 | 0.92 |
| OEOE_1856 | 1.26 | 1.37 | 1.39 | 1.23 | 1.30 | 1.16 |
|  |  |  |  |  |  |  |
| **Under-expressed genes** |  |  |  |  |  |  |
| OEOE_0053 | -1.54 | -1.47 | -1.26 | -0.94 | -0.79 | -1.03 |
| OEOE_0068 | -1.54 | -1.72 | -1.91 | -1.98 | -1.70 | -2.21 |
| OEOE_0074 | -1.53 | -1.46 | -1.28 | -0.95 | -0.78 | -1.02 |
| OEOE_0098 | -2.12 | -2.24 | -2.17 | -2.12 | -2.12 | -2.12 |
| OEOE_0118 | -1.03 | -1.26 | -1.07 | -1.02 | -1.02 | -1.01 |
| OEOE_0127 | -2.05 | -2.16 | -2.40 | -1.92 | -1.64 | -1.74 |
| OEOE_0210 | -2.51 | -2.69 | -2.27 | -2.18 | -1.96 | -2.25 |
| OEOE_0240 | -2.04 | -2.27 | -2.53 | -2.74 | -2.77 | -2.92 |
| OEOE_0315 | -2.15 | -2.00 | -2.52 | -2.49 | -2.42 | -2.28 |
| OEOE_0352 | -1.36 | -1.41 | -1.30 | -1.30 | -1.32 | -1.33 |
| OEOE_0359 | -0.82 | -1.00 | -1.07 | -0.92 | -0.92 | -0.92 |
| OEOE_0520 | -1.14 | -1.08 | -0.76 | -0.64 | -0.63 | -0.67 |
| OEOE_0527 | -0.66 | -0.96 | -1.26 | -1.45 | -1.51 | -1.56 |
| OEOE_0637 | -1.73 | -1.62 | -1.14 | -0.74 | -0.67 | -0.69 |
| OEOE_0708 | -3.71 | -3.42 | -3.53 | -3.19 | -3.21 | -3.35 |
| OEOE_0709 | -1.25 | -1.03 | -0.93 | -0.89 | -0.82 | -0.87 |
| OEOE_0729 | -0.72 | -1.03 | -0.98 | -0.97 | -0.90 | -0.90 |
| OEOE_0822 | -1.12 | -1.24 | -1.08 | -0.94 | -1.01 | -1.27 |
| OEOE_0823 | -1.08 | -1.12 | -0.80 | -0.74 | -0.82 | -0.83 |
| OEOE_0832 | -0.60 | -0.94 | -0.97 | -0.92 | -1.01 | -0.96 |
| OEOE_0855 | -1.75 | -1.53 | -1.54 | -1.64 | -1.79 | -1.71 |
| OEOE_0856 | -1.79 | -1.75 | -1.40 | -1.49 | -1.50 | -1.51 |
| OEOE_0858 | -0.90 | -0.82 | -0.81 | -1.04 | -0.95 | -1.19 |
| OEOE_0862 | -1.68 | -1.81 | -1.77 | -1.68 | -1.79 | -1.68 |
| OEOE_0871 | -1.08 | -0.21 | 0.21 | -0.02 | -0.21 | -0.34 |
| OEOE_0872 | -1.22 | -1.17 | -1.07 | -1.07 | -1.11 | -1.11 |
| OEOE_1022 | -1.79 | -1.74 | -1.47 | -0.92 | -0.62 | -0.54 |
| OEOE_1107 | -1.53 | -1.41 | -1.33 | -1.10 | -0.99 | -1.03 |
| OEOE_1122 | -0.76 | -0.82 | -0.97 | -1.03 | -1.04 | -1.06 |
| OEOE_1153 | -1.35 | -1.35 | -1.42 | -1.27 | -1.38 | -1.35 |
| OEOE_1198 | -0.38 | -0.57 | -1.02 | -0.66 | -0.50 | -0.28 |
| OEOE_1200 | -1.85 | -2.16 | -2.08 | -1.98 | -1.75 | -1.58 |
| OEOE_1203 | -1.72 | -1.42 | -1.20 | -1.10 | -1.05 | -1.29 |
| OEOE_1291 | -1.07 | -0.98 | -0.89 | -0.90 | -0.89 | -0.85 |
| OEOE_1302 | -1.35 | -1.45 | -1.44 | -1.48 | -1.44 | -1.49 |
| OEOE_1304 | -0.77 | -1.00 | -0.87 | -0.90 | -0.95 | -0.87 |
| OEOE_1305 | -1.34 | -1.31 | -1.43 | -1.61 | -1.48 | -1.60 |
| OEOE_1320 | -1.10 | -1.17 | -0.84 | -0.35 | -0.30 | -0.29 |
| OEOE_1329 | -1.15 | -1.43 | -1.43 | -1.44 | -1.49 | -1.67 |
| OEOE_1349 | -0.74 | -1.00 | -0.99 | -1.02 | -1.04 | -1.24 |
| OEOE_1350 | -1.47 | -1.82 | -1.82 | -1.99 | -2.12 | -1.99 |
| OEOE_1351 | -1.77 | -2.11 | -2.24 | -2.31 | -2.30 | -2.37 |
| OEOE_1356 | -2.56 | -2.45 | -2.81 | -2.63 | -2.59 | -2.56 |
| OEOE_1406 | -1.70 | -1.61 | -1.61 | -1.65 | -1.68 | -1.58 |
| OEOE_1453 | -1.32 | -1.05 | -0.88 | -1.13 | -1.10 | -1.20 |
| OEOE_1511 | -0.60 | -0.89 | -1.23 | -0.92 | -0.84 | -0.89 |
| OEOE_1535 | -1.74 | -1.54 | -1.29 | -1.02 | -0.95 | -0.73 |
| OEOE_1626 | -2.15 | -2.17 | -1.52 | -1.37 | -1.52 | -1.50 |
| OEOE_1654 | -0.78 | -1.02 | -1.09 | -1.06 | -1.04 | -0.99 |
| OEOE_1706 | -0.69 | -1.17 | -1.49 | -1.66 | -1.74 | -1.79 |
| OEOE_1751 | -0.77 | -0.72 | -0.95 | -1.05 | -1.04 | -0.92 |
