# Supplementary material for: Transcriptomic and Proteomic Analysis of Oenococcus oeni Adaptation to Wine Stress Conditions
Source: Front Microbiol. 2016 Sep 30;7:1554. doi: 10.3389/fmicb.2016.01554 (PMC5044463; doi:10.3389/fmicb.2016.01554)
Supplement: Supplementary file 3 [file Table3.DOCX]

**Table S3.** Proteomic identification and COG classification from 2D-DIGE and iTRAQ analysis.

| **COGs classification** | | |  | |  | | **Fold change** | | | | | **Theoretical Mr (KDa)** | **Pi** | | |
| --- | --- | --- | --- | --- | --- | --- | --- | --- | --- | --- | --- | --- | --- | --- | --- |
| **Old locus tag** | | **Gene symbol** | **Short name** | | **Protein annotation** | | **DIGE** | | | **iTRAQ** | | **DIGE/iTRAQ** | |  | |
| **RNA processing and modification ( A )** | | | | | |  | **1h** | **6h** | **1h** | | **6h** |  |  | |  |
| OEOE_1287 | OEOE_RS06200 | | |  | | metal-dependent hydrolase | 1.3 | 1.4 | - | | - | 25.2 | 4.9 | |  |
| **Energy production and conversion ( C )** | | | | | |  |  |  |  | |  |  |  | |  |
| OEOE_0663 | OEOE_RS03175 | | | atpA | | F0F1 ATP synthase subunit alpha | - | - | -1.3 | | 0.7 | 56.7 |  | |  |
| **Amino acid transport and metabolism ( E )** | | | | | |  |  |  |  | |  |  |  | |  |
| OEOE_0461 | OEOE_RS02220 | | | pepC | | aminopeptidase C | ND | -1.1 | - | | - | 50.5 | 5.2 | |  |
| OEOE_0574 | OEOE_RS02735 | | |  | | dipeptidase | - | - | 1.1 | | -0.8 | 41.2 |  | |  |
| OEOE_0991 | OEOE_RS04760 | | |  | | peptidase M20 | 1.3 | 1.4 | 1.3 | | 0.7 | 44.1 / 42.1 | 4.4 | |  |
| OEOE_1031 | OEOE_RS04955 | | |  | | glutamine amidotransferase | - | - | -1 | | 0.7 | 27.4 |  | |  |
| OEOE_1058 | OEOE_RS05080 | | | pepN | | aminopeptidase N | ND | -2.1 | 0.9 | | -1.1 | 95.1 | 5.1 | |  |
| OEOE_1191 | OEOE_RS05740 | | |  | | glutathione reductase | - | - | 2 | | 1 | 48.6 |  | |  |
| OEOE_1299 | OEOE_RS06260 | | |  | | succinate-semialdehyde dehdyrogenase | - | - | 1.6 | | 1.1 | 51.5 |  | |  |
| OEOE_1562 | OEOE_RS07535 | | | luxS | | S-ribosylhomocysteine lyase | 1.5 (3) | 1.6 (2) | 1.8 | | 1.5 | 17.7 | 5.3 | |  |
| OEOE_1783 | OEOE_RS08595 | | |  | | peptidase C69 | - | - | 1.9 | | 0.9 | 53.5 |  | |  |
| **Nucleotide transport and metabolism ( F )** | | | | | |  |  |  |  | |  |  |  | |  |
| OEOE_0519 | OEOE_RS02470 | | |  | | ADP-ribose pyrophosphatase | - | - | 0.8 | | 1.2 | 19.1 |  | |  |
| OEOE_0849 | OEOE_RS04085 | | |  | | deoxynucleoside kinase | -2.4 | -2.7 | - | | - | 25.8 | 5.6 | |  |
|  |  | | |  | |  | -1.5 | -1.6 | - | | - | 24.7 | 4.9 | |  |
| OEOE_1116 | OEOE_RS05365 | | |  | | ribonucleoside triphosphate reductase | - | - | 1 | | -1.7 | 82.8 |  | |  |
| OEOE_1598 | OEOE_RS07710 | | | tdk | | thymidine kinase | - | - | 0.7 | | -1.1 | 21.9 |  | |  |
| OEOE_1786 | OEOE_RS08610 | | | pyrG | | CTP synthetase | - | - | 1.6 | | -0.8 | 60.1 |  | |  |
| **Carbohydrate transport and metabolism ( G )** | | | | | |  |  |  |  | |  |  |  | |  |
| OEOE_0324 | OEOE_RS01550 | | |  | | aldehyde dehydrogenase | 1.2 (3) | 1.6 (2) | - | | - | 52.5 | 4.9 | |  |
| OEOE_0413 | OEOE_RS01985 | | |  | | lactate dehydrogenase | - | - | 1.5 | | 1.1 | 36.5 |  | |  |
| OEOE_0464 | OEOE_RS02230 | | |  | | PTS mannose transporter subunit EIIAB | - | - | -2.4 | | -0.7 | 35.6 |  | |  |
| OEOE_0563 | OEOE_RS02680 | | | hprK | | HPr kinase/phosphorylase | 1.6 | 1.9 | - | | - | 35.4 | 5.3 | |  |
| OEOE_0643 | OEOE_RS03075 | | | HPr | | phosphocarrier protein | - | - | -0.7 | | 2.3 | 9.0 |  | |  |
| OEOE_0647 | OEOE_RS03095 | | |  | | phosphoenol pyruvate-protein phosphotransferase | -1.2 | ND | - | | - | 63.2 | 5 | |  |
| OEOE_1024 | OEOE_RS04920 | | |  | | galactose mutarotase | - | - | 0.7 | | -1.1 | 34.1 |  | |  |
| OEOE_1182 | OEOE_RS05695 | | | ldhD | | D-lactate dehydrogenase | -1.3 | ND | - | | - | 36.5 | 5.7 | |  |
| OEOE_1402 | OEOE_RS06755 | | |  | | UDP-glucose 4-epimerase | - | - | 1.1 | | 0.7 | 36.9 |  | |  |
| OEOE_1602 | OEOE_RS07730 | | |  | | diacetyl reductase | ND | 1.2 | - | | - | 27.4 | 5.3 | |  |
|  |  | | |  | |  | ND | 1.6 | - | | - | 27.4 | 5 | |  |
| OEOE_1708 | OEOE_RS08245 | | |  | | fructokinase | -1.2 | ND | - | | - | 32.1 | 6.4 | |  |
| **Coenzyme transport and metabolism ( H )** | | | | | |  |  |  |  | |  |  |  | |  |
| OEOE_0791 | OEOE_RS03790 | | |  | | thiamine pyrophosphokinase | -2.1 | ND | - | | - | 25.4 | 4.7 | |  |
| OEOE_1036 | OEOE_RS04980 | | | pdxS | | pyridoxal biosynthesis lyase | - | - | -2.5 | | 1 | 31.4 |  | |  |
| **Lipid transport and metabolism ( I )** | | | | | |  |  |  |  | |  |  |  | |  |
| OEOE_1590 | OEOE_RS07670 | | |  | | ACP S-malonyltransferase | - | - | -0.8 | | 1.1 | 33.6 |  | |  |
| OEOE_1591 | OEOE_RS07675 | | |  | | 2-nitropropane dioxygenase | - | - | -1.5 | | 0.7 | 33.5 |  | |  |
| **Translation, ribosomal structure and biogenesis ( J )** | | | | | | |  |  |  | |  |  |  | |  |
| ND | OEOE_RS03845 | | | rplS | | 50S ribosomal protein L19 | - | - | -1.3 | | -0.7 | 14.6 | 4.7 | |  |
| ND | OEOE_RS06715 | | | rpsD | | 30S ribosomal protein S4 | - | - | -0.8 | | -2.5 | 23.3 | 5.4 | |  |
| OEOE_0010 | OEOE_RS00045 | | | rpsF | | 30S ribosomal protein S6 | - | - | -1 | | 0.8 | 16.3 | 5.8 | |  |
| OEOE_0433 | OEOE_RS02085 | | | infB | | translation initiation factor IF-2 | - | - | -1.1 | | -1.1 | 90.5 | 4.2 | |  |
| OEOE_0460 | OEOE_RS02215 | | | thrS | | threonyl-tRNA synthase | -2.0 | -3.3 | -0.7 | | -1.1 | 76.3 |  | |  |
| OEOE_0596 | OEOE_RS02850 | | | rplD | | 50S ribosomal protein L4 | - | - | -0.7 | | -1.4 | 22.4 |  | |  |
| OEOE_0600 | OEOE_RS02870 | | | rplV | | 50S ribosomal protein L22 | - | - | -1 | | 0.7 | 13.1 |  | |  |
| OEOE_0601 | OEOE_RS02875 | | | rpsC | | 30S ribosomal protein S3 | - | - | -1.3 | | -0.7 | 29.9 |  | |  |
| OEOE_0607 | OEOE_RS02905 | | | rplE | | 50S ribosomal protein L5 | - | - | -1.5 | | -0.8 | 20.0 |  | |  |
| OEOE_0608 | OEOE_RS02910 | | | rpsH | | 30S ribosomal protein S8 | - | - | -1.5 | | 1 | 14.6 |  | |  |
| OEOE_0613 | OEOE_RS02935 | | | rplO | | 50S ribosomal protein L15 | - | - | 0.7 | | 2.3 | 16.5 |  | |  |
| OEOE_0621 | OEOE_RS02970 | | | rplQ | | 50S ribosomal protein L17 | - | - | -0.7 | | 1.2 | 14.9 |  | |  |
| OEOE_0770 | OEOE_RS03680 | | | rpmF | | 50S ribosomal protein L32 | - | - | 0.7 | | 1.2 | 6.7 |  | |  |
| OEOE_0792 | OEOE_RS03795 | | | tuf | | elongation factor Tu | -1.3 | -1.6 | -1.6 | | -0.9 | 43.6 | 4.9 | |  |
| OEOE_0976 | OEOE_RS04685 | | | tsf | | elongation factor Ts | - | - | -2.3 | | -0.9 | 31.8 |  | |  |
| OEOE_0978 | OEOE_RS04695 | | | frr | | ribosome recycling factor | -1.6 | ND | - | | - | 20.4 |  | |  |
| OEOE_1030 | OEOE_RS04950 | | | rpsN | | 30S ribosomal protein S14 | - | - | -1.7 | | -0.7 | 10.0 |  | |  |
| OEOE_1284 | OEOE_RS06185 | | | rpsT | | 30S ribosomal protein S20 | - | - | 0.7 | | 1 | 9.8 |  | |  |
| OEOE_1314 | OEOE_RS06335 | | | fusA | | elongation factor G | -1.7 | 1 | - | | - | 77.9 |  | |  |
| OEOE_1392 | OEOE_RS06700 | | | valS | | valyl-tRNA synthase | - | - | 0.8 | | -1.9 | 104.6 |  | |  |
| OEOE_1416 | OEOE_RS06825 | | | rplL | | 50S ribosomal protein L7/L12 | ND | 2.0 | - | | - | 12.2 | 4.2 | |  |
| OEOE_1746 | OEOE_RS08425 | | | metG | | methionyl-tRNA synthetase | - | - | -0.7 | | -1.1 | 77.0 |  | |  |
| OEOE_1784 | OEOE_RS08600 | | | rpmE2 | | 50S ribosomal protein L31 type B, partial | - | - | -0.9 | | 2.1 | 6.5 |  | |  |
| **Transcription ( K )** | | | | | |  |  |  |  | |  |  |  | |  |
| OEOE_0561 | OEOE_RS02670 | | |  | | PhoU family transcriptional regulator | - | - | 0.7 | | -1.1 | 25.9 |  | |  |
| OEOE_1376 | OEOE_RS06620 | | |  | | cold-shock protein | 1.3 | ND | - | | - | 7.4 | 4.7 | |  |
| **Replication, recombination and repair ( L )** | | | | | |  |  |  |  | |  |  |  | |  |
| OEOE_0011 | OEOE_RS00050 | | |  | | single-stranded DNA-binding protein | ND | 1.4 | - | | - | 20 | 4.6 | |  |
| OEOE_0372 | OEOE_RS01785 | | | uvrB | | excinuclease ABC subunit B | - | - | 0.7 | | -1.8 | 76.6 |  | |  |
| OEOE_0426 | OEOE_RS02050 | | | pcrA | | ATP-dependent DNA helicase | - | - | 0.7 | | -1 | 85.0 |  | |  |
| OEOE_1072 | OEOE_RS05155 | | | obg | | GTPase CgtA | - | - | 0.7 | | -1.1 | 48.2 |  | |  |
| OEOE_1382 | OEOE_RS06650 | | | recA | | protein RecA | - | - | -0.7 | | -1.1 | 43.5 |  | |  |
| **Cell wall/membrane/envelope biogenesis ( M )** | | | | | | |  |  |  | |  |  |  | |  |
| OEOE_0668 | OEOE_RS03200 | | | mreB | | rod shape-determining protein | - | - | -1.2 | | -1.1 | 40.1 |  | |  |
| OEOE_1444 | OEOE_RS06975 | | |  | | peptidoglycan interpeptide bridge formation protein | - | - | -1.1 | | -1 | 39.4 |  | |  |
| OEOE_1561 | OEOE_RS07530 | | |  | | D-alanyl-D-alanine carboxypeptidase | - | - | -0.8 | | -1.5 | 31.0 |  | |  |
| **Post-translational modification, protein turnover, and chaperones ( O )** | | | | | | |  |  |  | |  |  |  | |  |
| OEOE_1114 | OEOE_RS05355 | | | sufC | | Fe-S cluster assembly ABC-type transport system,. ATPase component | -1.9 | -2.1 | - | | - | 28.2 | 5.7 | |  |
| OEOE_1396 | OEOE_RS06725 | | | groL | | molecular chaperone GroEL | 1.1 | 1.4 (2) | - | | - | 57.5 | 5.0 | |  |
| OEOE_1397 | OEOE_RS06730 | | | groS | | Co-chaperonin GroES (HSP10) | 1.7 | 1.8 (2) | - | | - | 9.7 | 4.7 | |  |
| OEOE_1625 | OEOE_RS07835 | | |  | | thiol reductase thioredoxin | - | - | -0.6 | | 1.4 | 12.6 |  | |  |
| OEOE_1702 | OEOE_RS08215 | | |  | | thiol reductase thioredoxin | - | - | -0.7 | | 1.3 | 11.5 |  | |  |
| **Inorganic ion transport and metabolism ( P )** | | | | | |  |  |  |  | |  |  |  | |  |
| OEOE_1111 | OEOE_RS05340 | | |  | | Fe-S cluster assembly protein SufB | - | - | 0.7 | | -1.1 | 51.7 |  | |  |
| OEOE_1705 | OEOE_RS08230 | | |  | | glyoxalase | 1.3 | 1.4 | - | | - | 18.5 | 6.1 | |  |
| OEOE_1750 | OEOE_RS08440 | | |  | | DNA-binding ferritin-like protein (oxidative damage protectant) | 1.6 | 1.8 | - | | - | 18.3 | 4.4 | |  |
| **General function prediction only ( R )** | | | | | |  |  |  |  | |  |  |  | |  |
| OEOE_0070 | OEOE_RS00320 | | | ara1 | | 2,5-diketo-D-gluconic acid reductase | 1.3 | ND | 1.3 | | 1.1 | 31.7 | 5.5 | |  |
| OEOE_0156 | OEOE_RS00735 | | | nnrE | | NAD(P)H-hydrate epimerase | - | - | -0.7 | | -1.1 | 22.5 |  | |  |
| OEOE_0518 | OEOE_RS02465 | | |  | | 2,5-diketo-D-gluconic acid reductase | ND | 1.4 | - | | - | 32 | 5.3 | |  |
| OEOE_0693 | OEOE_RS03325 | | |  | | acetoin reductase | - | - | -1.7 | | -0.9 | 27.4 |  | |  |
| OEOE_0904 | OEOE_RS04335 | | |  | | 3'-5' exoribonuclease | - | - | 1.1 | | -0.7 | 36.3 |  | |  |
| OEOE_0936 | OEOE_RS04485 | | | pox | | pyruvate oxidase | - | - | 1.7 | | -0.8 | 64.0 |  | |  |
| OEOE_1336 | OEOE_RS06430 | | |  | | 1,3-propanediol dehydrogenase | ND | 1.4 | -0.7 | | 2.4 | 42.1 / 37.6 | 5.2 | |  |
| OEOE_1782 | OEOE_RS08590 | | |  | | aldo/keto reductase | 1.3 | 1.5 (2) | 1.3 | | 0.7 | 35.9 | 5.2 | |  |
| **Function unknown ( S )** | | | | | |  |  |  |  | |  |  |  | |  |
| OEOE_0683 | OEOE_RS03275 | | |  | | hypothetical protein | 1.3 | ND | - | | - | 18 | 4.6 | |  |
| **Signal transduction mechanisms ( T )** | | | | | |  |  |  |  | |  |  |  | |  |
| OEOE_0807 | OEOE_RS03875 | | |  | | GTP-binding protein | ND | 2.6 | -0.8 | | -1.1 | 68.2 | 5.1 | |  |
| **Multifunctional** | | | | | |  |  |  |  | |  |  |  | |  |
| OEOE_0002 | OEOE_RS00010 | | |  | | DNA polymerase III subunit beta | ND | 1.3 | 1.5 | | 0.9 | 40.7 | 5.15 | |  |
| OEOE_0122 | OEOE_RS00565 | | |  | | phosphoglycerate mutase | -1.6 | -1.5 | - | | - | 27.1 | 5.4 | |  |
| OEOE_0159 | OEOE_RS00750 | | |  | | ribose-5-phosphate isomerase A | 1.1 | 1.4 | - | | - | 25.2 | 5 | |  |
| OEOE_0160 | OEOE_RS00755 | | | prs | | ribose-phosphate pyrophosphokinase | - | - | -1.5 | | -0.9 | 36.9 |  | |  |
| OEOE_0258 | OEOE_RS01235 | | | pyrB | | aspartate carbamoyltransferase | -1.3 | 1.5 (2) | - | | - | 35 / 38.9 | 6.11 | |  |
| OEOE_0404 | OEOE_RS01940 | | | gapA | | glyceraldehyde-3-phosphate dehydrogenase | 1.2 | ND | 1.1 | | 0.8 | 37.1 | 5.85 | |  |
| OEOE_0418 | OEOE_RS02010 | | | mae | | malate dehydrogenase | - | - | -1 | | -0.8 | 41.4 |  | |  |
| OEOE_0527 | OEOE_RS02510 | | |  | | zinc-dependent alcohol dehydrogenase | 1.5 (2) | 1.5 (4) | 1.5 | | 1 | 37.5 | 4.9 | |  |
| " | " | | |  | | " " | 1.2 | 1.3 |  | |  | 37.5 | 5.24 | |  |
| OEOE_0564 | OEOE_RS02685 | | | gpsA | | glycerol-3-phosphate dehydrogenase | - | - | 1.8 | | 0.7 | 36.5 |  | |  |
| OEOE_0615 | OEOE_RS02945 | | |  | | adenylate kinase | - | - | -0.9 | | 1.1 | 20.7 |  | |  |
| OEOE_0620 | OEOE_RS02965 | | | rpoA | | DNA-directed RNA polymerase subunit alpha | - | - | 0.9 | | 1.3 | 34.5 |  | |  |
| OEOE_0635 | OEOE_RS03035 | | | glmS | | glucosamine--fructose-6-phosphate aminotransferase | - | - | 1.7 | | 1.1 | 66.2 |  | |  |
| OEOE_0636 | OEOE_RS03040 | | | pgi | | glucose-6-phosphate isomerase | - | - | 1.9 | | -0.8 | 48.6 |  | |  |
| OEOE_0774 | OEOE_RS03705 | | | dapA | | 4-hydroxy-tetrahydrodipicolinate synthase | ND | 1.3 | - | | - | 31.9 | 5.7 | |  |
| OEOE_0790 | OEOE_RS03785 | | |  | | ribulose-phosphate 3-epimerase | ND | 1.5 | - | | - | 24 | 5.1 | |  |
| OEOE_0892 | OEOE_RS04275 | | |  | | phosphogluconate dehydrogenase (NADP(+)-dependent. decarboxylating) | 1.2 (2) | 1.3 | 1.8 | | 1 | 53 | 5.3 | |  |
| OEOE_0920 | OEOE_RS04415 | | | glk | | glucokinase | - | - | 1.6 | | 0.8 | 34.3 |  | |  |
| OEOE_0952 | OEOE_RS04565 | | | glnA | | glutamine synthetase | - | - | 0.6 | | 1 | 49.9 |  | |  |
| OEOE_1002 | OEOE_RS04810 | | | pyk | | pyruvate kinase | ND | -1.4 | - | | - | 52.2 | 6.3 | |  |
| OEOE_1249 | OEOE_RS06020 | | | ackA | | acetate kinase | - | - | 1.9 | | 0.7 | 43.9 |  | |  |
| OEOE_1269 | OEOE_RS06110 | | | murC | | UDP-N-acetylmuramate--L-alanine ligase | - | - | 0.6 | | -1.6 | 48.1 |  | |  |
| OEOE_1285 | OEOE_RS06190 | | |  | | DEAD/DEAH box helicase | - | - | -0.7 | | -1 | 50.4 |  | |  |
| OEOE_1374 | OEOE_RS06610 | | | rpoC | | DNA-directed RNA polymerase subunit beta' | - | - | -0.8 | | -1.5 | 137.0 |  | |  |
| OEOE_1375 | OEOE_RS06615 | | | rpoB | | DNA-directed RNA polymerase subunit beta | - | - | -0.6 | | -1.5 | 133.0 |  | |  |
| OEOE_1421 | OEOE_RS06850 | | |  | | gluconokinase | - | - | 1 | | -0.8 | 57.3 |  | |  |
| OEOE_1435 | OEOE_RS06930 | | | pta | | phosphate acetyltransferase | - | - | -1.3 | | -0.6 | 35.3 |  | |  |
| OEOE_1523 | OEOE_RS07345 | | | gnd | | 6-phosphogluconate dehydrogenase | 1.12 | ND | - | | - | 32.8 | 4.94 | |  |
| OEOE_1641 | OEOE_RS07915 | | |  | | alanine racemase | - | - | 1.4 | | -0.6 | 43.0 |  | |  |
| OEOE_1643 | OEOE_RS07925 | | |  | | DEAD/DEAH box helicase | - | - | -1.8 | | -0.8 | 56.9 |  | |  |
| OEOE_1650 | OEOE_RS07960 | | | eno | | enolase | 1.5 | 1.5 | 0.7 | | 1.8 | 48.4 / 47.3 | 4.6 | |  |
| " | " | | |  | | " |  | 1.3 |  | |  | 48.4 | 4.8 | |  |
| OEOE_1707 | OEOE_RS08240 | | |  | | Threonine dehydrogenase-like Zn-dependent dehydrogenase | 1.5 | 1.6 | - | | - | 36.1 | 5.6 | |  |
| OEOE_1780 | OEOE_RS08580 | | |  | | alcohol dehydrogenase | ND | 1.6 | - | | - | 36.4 | 4.9 | |  |
| " | " | | |  | | " |  | 1.6 |  | |  | 37.6 | 5 | |  |
| OEOE_1785 | OEOE_RS08605 | | | murA | | UDP-N-acetylglucosamine 1-carboxyvinyltransferase | - | - | 0.7 | | 1 | 45.7 |  | |  |
| OEOE_1788 | OEOE_RS08615 | | | rpoE | | DNA-directed RNA polymerase subunit delta | - | - | -1 | | 1.3 | 21.9 |  | |  |
